# LIGHT (TNFSF14) promotes the differentiation of human bone marrow-derived mesenchymal stem cells into functional hepatocyte-like cells

**DOI:** 10.1371/journal.pone.0289798

**Published:** 2023-08-08

**Authors:** Sook-Kyoung Heo, Ho-Min Yu, Do Kyoung Kim, Hye Jin Seo, Yerang Shin, Sung Ah Kim, Minhui Kim, Youjin Kim, Yoo Jin Lee, Eui-Kyu Noh, Jae-Cheol Jo

**Affiliations:** 1 Biomedical Research Center, Ulsan University Hospital, University of Ulsan College of Medicine, Ulsan, Republic of Korea; 2 Department of Hematology and Oncology, Ulsan University Hospital, University of Ulsan College of Medicine, Ulsan, Republic of Korea; National Research Centre, EGYPT

## Abstract

Liver transplantation is the most effective treatment option for patients with acute or chronic liver failure. However, the applicability and effectiveness of this modality are often limited by a shortage of donors, surgical complications, high medical costs, and the need for continuing immunosuppressive therapy. An alternative approach is liver cell transplantation. LIGHT (a member of the tumor necrosis factor superfamily) could be a promising candidate for promoting the differentiation of human bone marrow-derived mesenchymal stem cells (hBM-MSCs) into hepatocyte-like cells. In this study, we investigated the effect of LIGHT on hBM-MSC differentiation into hepatocyte-like cells. Our previous results showed that LIGHT receptor lymphotoxin-β receptor (LTβR) is constitutively expressed on the surface of hBM-MSCs. Upon treatment with recombinant human LIGHT (rhLIGHT), the phenotype of hBM-MSCs changed to round or polygonal cells. In addition, the cells exhibited high levels of hepatocyte-specific markers, including albumin, cytokeratin-18 (CK-18), CK-19, cytochrome P450 family 1 subfamily A member 1 (CYP1A1), CYP1A2, CYP3A4, SRY-box transcription factor 17 (SOX17), and forkhead box A2 (FOXA2). These results indicate that rhLIGHT enhances the differentiation of hBM-MSCs into functional hepatocyte-like cells. Furthermore, rhLIGHT-induced hepatocyte-like cells showed a higher ability to store glycogen and uptake indocyanine green compared with control cells, indicating functional progression. Additionally, treatment with rhLIGHT increased the number, viability, and proliferation of cells by inducing the S/G2/M phase and upregulating the expression of various cyclin and cyclin dependent kinase (CDK) proteins. We also found that the hepatogenic differentiation of hBM-MSCs induced by rhLIGHT was mediated by the activation of signal transducer and activator of transcription 3 (STAT3) and STAT5 pathways. Overall, our findings suggest that LIGHT plays an essential role in promoting the hepatogenic differentiation of hBM-MSCs. Hence, LIGHT may be a valuable factor for stem cell therapy.

## Introduction

The liver is the largest internal organ, playing essential roles in metabolism and survival [1]. It typically performs several functions, such as detoxification, protein synthesis, nutrient storage, and the production of gallbladder, urea, and bile [2]. Specifically, it stores glycogen, decomposes red blood cells, synthesizes serum proteins, and produces hormones.

Acute-chronic liver failure, including liver fibrosis and cirrhosis, is a major health problem worldwide, with a prevalence of 20%–35% in the at-risk population [3]. Liver transplantation is the best treatment option for patients with end-stage liver disease [4]. However, the applicability and effectiveness of this modality is limited by a shortage of donors, surgical complications, high medical costs, and the burden of continuous use of immunosuppressants. Hepatocyte transplantation is an alternative treatment option that can used to overcome these limitations [5], offering several advantages compared with liver transplantation. First, hepatocytes obtained from a single donor can be supplied to multiple recipients. Second, the surgical burden associated with hepatocyte transplantation is low. Finally, this approach can serve as a temporary solution until liver recovery. However, hepatocytes cannot sufficiently proliferate for a long period of time *in vitro* and *in vivo*, thereby posing a problem to hepatocyte transplantation [5]. In contrast, hepatocyte transplantation of xenogeneic hepatocytes using pig liver can provide an unlimited number of hepatocytes; nevertheless, this approach has also been linked to problems, such as immune rejection and viral infection [6]. For this reason, recently, the use of stem cells to repair damaged tissues and organs has attracted considerable attention. Moreover, research studies are currently ongoing to regenerate damaged livers and improve their functions through the transplantation of stem cells isolated from embryos, umbilical cord blood, or adults [7, 8].

Stem cells have tremendous therapeutic potential, particularly in regenerative medicine. Among them, mesenchymal stem cells (MSCs) are multipotent cells with great potential as novel therapeutic agents [9, 10]. MSCs are fibroblast-like plastic adherent cells that expand rapidly *in vitro* under standard culture conditions. They are present in various tissues and organs, including the bone marrow-derived MSCs (BM-MSCs) [11], umbilical cord blood [12], synovium [13], adipose tissues [14, 15], placenta [16], dental pulp [17], Whartons’ jelly [18], fetal lungs and blood [19], etc. These cells can differentiate into various types, including osteogenic, chondrogenic, and adipogenic lineages [20, 21], as well as osteoblasts, chondrocytes, adipocytes, keratinocytes, neural cells, muscle cells, and hepatocytes [22]. Therefore, MSCs can be used in various repair processes [23], such as bone regeneration, cartilage repair, regeneration of other musculoskeletal tissues, myocardium restoration, peripheral nervous system reconstruction, liver regeneration, corneal reconstruction, etc. Moreover, MSCs have low immunogenicity and immunomodulatory properties, thereby evading host immune surveillance [24, 25].

LIGHT (tumor necrosis factor [TNF] superfamily member 14 [TNFSF14], CD258, herpes virus entry mediator-ligand [HVEM-L]) is a cytokine primarily expressed in activated T cells, monocytes, granulocytes, and spleen cells [26]. It has three receptors, namely lymphotoxin-β receptor (LTβR; TNFRSF3), HVEM (TNFRSF14, CD270), and decoy receptor-3 (DcR3; TNFRSF6B) [27]. LTβR is expressed in fibroblasts, endothelial cells, epithelial cells, and stromal cells [28–30]. Moreover, it is also constitutively expressed in human BM-MSCs (hBM-MSCs) [20, 31]. LTβR, one of the membrane-bound receptors of LIGHT, controls organogenesis and plays a critical role in immune system development and immune responses during embryonic development [32].

Previously, we showed that interaction between LIGHT and LTβR increases the survival and proliferation of hBM-MSCs [20]. However, there is limited knowledge regarding the role of this interaction in the hepatogenic differentiation of hBM-MSCs. Therefore, in this study, we investigated the importance of LIGHT in the differentiation of hBM-MSCs into functional hepatocyte-like cells.

## Materials and methods

### Reagents

Recombinant human LIGHT (rhLIGHT) was purchased from R&D Systems (Minneapolis, MN, USA). Anti-human CD90-fluorescein isothiocyanate (CD90-FITC), anti-human CD44-FITC, anti-human CD105-FITC, anti-human CD34-FITC, anti-human CD45-FITC, anti-human CD19-FITC, anti-LTβR-PE, and FITC Mouse immunoglobulin G (IgG)-isotype control antibodies were purchased from BD Biosciences (San Diego, CA, USA). The Mesenchymal Stem Cell Growth Medium BulletKit™ (MSCGM™) was obtained from Lonza (Basel, Switzerland). For western blotting, antibodies against albumin, SRY-box transcription factor 17 (SOX17), forkhead box A2 (FOXA2), cyclin dependent kinase 1 (CDK1), CDK6, cyclin B1, cyclin D3, B-cell lymphoma-extra large (Bcl-xl), inhibitor of κB-α (IκB-α), protein kinase B (AKT), phospho-AKT, signal transducer and activator of transcription 3 (STAT3), phospho-STAT3, STAT5, phospho-STAT5, Rabbit IgG-horseradish peroxidase (IgG-HRP), and Mouse IgG-HRP were purchased from Cell Signaling Technology (Danvers, MA, USA). Antibodies against cytokeratin-18 (CK-18), CK-19, cytochrome P450 family 1 subfamily A member 1 (CYP1A1), CYP1A2, CYP3A4, CDK2, CDK4, and β-actin were purchased from Santa Cruz Biotechnology (Santa Cruz, Dallas, TX, USA). All reagents were obtained from Sigma–Aldrich (St. Louis, MO, USA).

### BM-MSC isolation and culture

The isolation and culture of hBM-MSC were performed as previously described [20, 31]. The hBM-MSCs used in this study were passaged 3–8 times and maintained in MSC basal medium MSCGM™ (Lonza) in a humidified environment with 5% CO_2_ at 37°C.

### Ethics approval

All human-related methods were conducted in accordance with the relevant guidelines and regulations. Donors provided written informed consent prior to the commencement of the study. BM samples of donors were collected once from healthy volunteers participating in this study at the Ulsan University Hospital, Ulsan, South Korea. Participants were recruited for a period of two years starting from November 1, 2016, and the study was conducted over a span of four years beginning on December 1, 2018. The study protocol and consent form and information were approved by the Ulsan University Hospital Ethics Committee and Institutional Review Board (Approval number: UUH-IRB-2016-07-026).

### HepG2 cell culture

In this study, human hepatocellular carcinoma HepG2 cells were cultured and maintained in high glucose Dulbecco’s modified Eagle’s medium supplemented with 10% fetal bovine serum and a 1% penicillin-streptomycin solution (final concentration: 100 units/ml and 100 μg/ml, respectively) in a humidified atmosphere with 5% CO_2_ at 37°C. HepG2 cells were utilized as a positive control in this study.

### Marker staining by flow cytometry analysis

The hBM-MSCs were harvested and washed twice with fluorescence-activated cell sorter (FACS) buffer (phosphate-buffered saline [PBS] containing 0.2% bovine serum albumin and 0.1% NaN_3_). Cells were incubated with antibodies against cell surface antigens, positive markers (e.g., CD44, CD90, and CD105), and negative markers (e.g., CD19, CD34, and CD45) on ice for 30 min. In addition, staining for the LTβR antigen was performed in hBM-MSCs. Next, cells were washed twice with FACS buffer and analyzed using the FACS Calibur flow cytometer and CellQuest Pro software (BD Biosciences).

### Hepatogenic differentiation protocol

**Fig 1:**
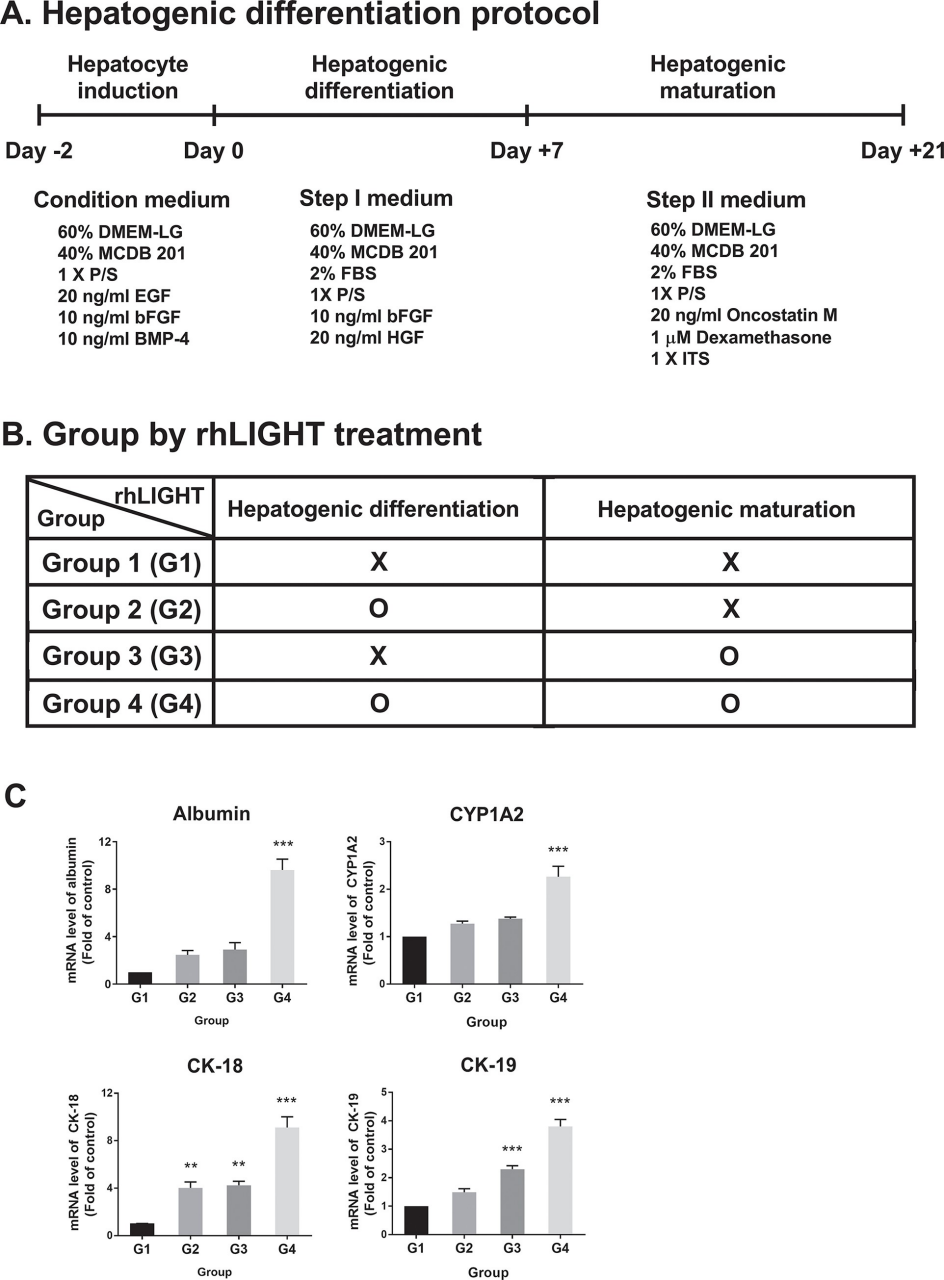
Effect of rhLIGHT on the induction of differentiation from human bone marrow-derived mesenchymal stem cells (hBM-MSCs) to hepatocyte-like cells. (A) Protocol for inducing the differentiation of hBM-MSCs into hepatocyte-like cells by treatment with rhLIGHT. (B) Group settings according to the treatment with rhLIGHT treatment. (C) RNA expression levels of albumin, CYP1A2, CK-18, and CK-19, as determined by qRT-PCR analysis. Data represent the mean ± SEM. *Significantly different from control cells; **, *P* < 0.01; ***, *P* < 0.001.

The hepatogenic differentiation protocol can be divided into three major steps: hepatocyte induction, hepatogenic differentiation, and hepatogenic maturation. In addition, each culture medium is required for each stage. The hepatogenic differentiation protocol used is shown in Fig 1A. First, 3×10^5^ cells were seeded in 60-mm culture dishes in conditioned medium. It is composed of 60% DMEM-LG, 40% MCDB 201, 1 × P/S, 20 ng/ml EGF, 10 ng/ml bFGF, 10 ng/ml BMP-4. After 2 days, the medium was replaced with Step I medium for hepatogenic differentiation. It supplemented with 60% DMEM-LG, 40% MCDB 201, 2% FBS, 1× P/S, 10 ng/ml bFGF, 20 ng/ml HGF. After 7 days, the medium was replaced with Step II medium for hepatogenic maturation. Step II medium included 60% DMEM-LG, 40% MCDB 201, 2% FBS, 1× P/S, 20 ng/ml oncostatin M, 1 μM Dexamethasone, 1 × ITS. The composition of the hepatocyte differentiation medium is detailed in S1 Fig in S1 File. All assays were performed with undifferentiated BM-MSCs as negative controls and HepG2 cells as positive controls.

### Real-time quantitative reverse transcription-polymerase chain reaction (qRT-PCR)

BM-MSCs were cultured with various conditions of rhLIGHT for 21 days, as shown in Fig 1B (G1, G2, G3, G4). Total RNA was isolated from cells using the TRIzol Reagent (Sigma–Aldrich), and cDNA was synthesized using Superscript III Reverse Transcriptase according to the instructions provided by the manufacturer (Invitrogen, Grand Island, NY, USA). The cDNA was analyzed with the CFX96 real-time PCR system using iQ SYBR Green supermix (BioRad, Hercules, CA, USA). All primers used in this experiment were synthesized by Bioneer Corporation (Daejeon, Korea). The primer sequences are shown in Table 1. The PCR conditions were as follows: an initial denaturation step at 95°C for 3 min and 40 cycles at 95°C for 10 s, 63°C for 10 s, and 72°C for 30 s. During the 72°C extension step, optical detection was performed to determine SYBR green fluorescence. The expression of the target gene relative to that of the endogenous control gene glyceraldehyde-3-phosphate dehydrogenase was calculated using the difference in the threshold cycle method (ΔC_t_ = C_t’_ target − C_t’_ control), as described in our previous report [31, 33], in which the relative expression equaled 2^−ΔΔCt^ (ΔΔC_t_ = ΔC_t’_ target − ΔC_t’_ untreated). All assays were performed in triplicate.

**Table 1:** Sequences of primers used for quantitative RT-PCR.

Target gene	Primer sequences (5’ →3’)	
**Albumin**	**Forward**	**CCAAGTGTCAACTCCAACTCT**
	**Reverse**	**TCTGCACAGGGCATTCTTT**
**CYP1A2**	**Forward**	**CTTCCCAAAGTCCTGGGATTAC**
	**Reverse**	**CTGGTGATGGTTGCACAATTC**
**CK-18**	**Forward**	**GACCTGGACTCCATGAGAAATC**
	**Reverse**	**GTTGAGCTGCTCCATCTGTA**
**CK-19**	**Forward**	**GTGACATGCGAAGCCAATATG**
	**Reverse**	**GACCTCCCGGTTCAATTCTT**
**LTβR**	**Forward**	**GGCACCTATGTCTCAGCTAAAT**
	**Reverse**	**GTAGTTCCAGTGCTCGTTGTAG**
**GAPDH**	**Forward**	**GATCATCAGCAATGCCTCCT**
	**Reverse**	**GTCATGAGTCCTTCCACGATAC**

CYP1A2, Cytochrome P450 1A2; LTβR, Lymphotoxin beta receptor; CK-18, Cytokeratin-18; CK-19, Cytokeratin-19; GAPDH, Glyceraldehyde 3-phosphate dehydrogenase.

### Western blotting analysis

The samples were washed thrice with ice-cold PBS and harvested. Western blotting was performed as previously described [34, 35]. MSCs and HepG2 cells were considered as negative and positive control, respectively. In some experiments, the STAT inhibitor WP1066 was used to block STAT3 and STAT5 signals.

### Indocyanine green uptake assay

The culture media were replaced with 1 mg/ml of indocyanine green prepared in culture media. After incubation at 37°C for 30 min, cells were rinsed thrice with PBS, and ICG uptake was measured using an inverted microscope. HepG2 cells were utilized as a positive control. The relative intensity of indocyanine green uptake was measured using the ImageJ program.

### Periodic acid–Schiff staining (PAS)

Glycogen storage by hepatocyte-like cells was evaluated through PAS staining. Differentiated cells were fixed with 10% paraformaldehyde. After washing, the specimens were incubated with 0.5% periodic acid for 5 min, rinsed thrice with distilled water, treated with Schiff’s reagent for 15 min in the dark, and rinsed again with distilled water for 5 min. The levels of glycogen in the cells were subsequently visualized by incubating the cells with Schiff’s reagent. A light microscope was used for the visualization. HepG2 cells were utilized as a positive control. The relative intensity of glycogen by PAS staining was measured using the ImageJ program.

### Trypan blue exclusion assay

Trypan blue exclusion assay was performed as previously described [20]. Cells excluding trypan blue (i.e., viable cells) were counted under a microscope with a hemocytometer. Each test was performed at least four times. In the cytotoxicity assay of N-Nitrosodiethylamine (NDEA), the initial number of cells used is 8 × 10^4^ cells per well. After treatment with 0 mM and 1 mM NDEA, the number of live cells per well was counted after 24 hours.

### Cell viability assay (MTS assay)

Cell viability assay was performed as previously described [20, 36]. According to the hepatocyte differentiation protocol, cell viability analysis was performed using the cells in which hepatocyte maturation was completed. In particular, the experiment focused on the relative cell viability of the G1 and G4 groups. Cell viability was measured with the CellTiter 96 AQueous One Solution Cell Proliferation Assay (Promega, Madison, WI, USA) according to the assay manufacturer’s protocol. The assay was performed by adding CellTiter 96 solution to each well and the plate was incubated for an additional 4 hours at 37° C. in a humidified 5% CO2 atmosphere. Finally, absorbance was measured at 490 nm using a SpectraMax iD3 microplate reader (Molecular Devices, San Jose, CA, USA)

### Cell proliferation assay (BrdU assay)

BM-MSCs were examined under the same conditions as above. Cell proliferation was measured by BrdU (5′-bromo-2-deoxyuridine) enzyme-linked immunosorbent assay (Cell Proliferation ELISA, BrdU; Roche Diagnostics), according to the manufacturer’s instructions. The results are expressed as percentage changes from the basal condition using three to five culture wells for each experimental condition.

### Cell cycle analysis by flow cytometry

Cell cycle analysis were performed as previously described [20]. Briefly, BM-MSCs were examined under the same conditions as above. They were washed twice with PBS and fixed with 70% ethanol overnight at -20°C, followed by washing again with PBS and incubation with 0.5 ml PI/RNase stain buffer for 15 min at room temperature. The samples were then analyzed using a FACSCalibur flow cytometer and CellQuest Pro software (BD Biosciences).

### Statistical analysis

Data are presented as means ± the standard error of the mean based on at least three independent experiments. All values were evaluated through one-way analysis of variance, followed by Tukey’s range test. The analyses were performed using the GraphPad Prism 7.0 software (GraphPad Software, Inc., La Jolla, CA, USA). A *p* < 0.05 denoted statistically significant differences. Each treatment was assayed in triplicate.

## Results

### LIGHT promoted hepatocyte-specific marker proteins in hBM-MSCs

In our previous study, to confirm the quality of MSCs, we examined fat formation (adipogenesis) with Oil Red O staining, cartilage formation (chondrogenesis) by Alcian blue staining, and bone formation (osteogenesis) through Alizarin red staining [20]. We assessed positive and negative differentiation markers of hBM-MSCs using flow cytometry analysis, as shown in S1A and S1B Fig in S1 File. The MSCs used in our experiments were consistently positive for CD90, CD44, and CD105 and negative for CD34, CD45, and CD19. In addition, LTβR was constitutively and highly expressed on the surface of hBM-MSCs (S1C Fig in S1 File).

Before initiating this study, we monitored whether hBM-MSCs could differentiate into hepatocyte-like cells *in vitro*. Hepatocyte-like cells were observed after 21 days of culture in liver differentiation medium containing hepatocyte growth factor, fibroblast growth factor-2 (FGF-2), oncostatin M (OSM), and ITS Liquid Media Supplement. The hepatocyte differentiation protocol used in this experiment is shown in Fig 1A. Based on this, we evaluated the role of LIGHT in the differentiation of hBM-MSCs into functional hepatocyte-like cells. In addition, the cells were divided into G1, G2, G3, and G4 groups according to the treatment schedule of LIGHT, as shown in Fig 1B. Initially, to investigate the effect of LIGHT on the differentiation of hBM-MSCs into hepatocyte-like cells, we observed the expression of hepatocyte-specific marker genes (e.g., albumin, CYP1A2, CK-18, and CK-19) using qRT-PCR. As shown in Fig 1C, there was a difference in the expression levels of hepatocyte marker genes between groups. Among them, the greatest difference was noted between the G4 group (i.e., hBM-MSCs incubated with 50 ng/ml rhLIGHT for 21 days) and G1 group (i.e., the control group, hBM-MSCs incubated with 0 ng/ml rhLIGHT for 21 days). These results showed that LIGHT plays an important role in hepatogenic differentiation and maturation. Therefore, only the G1 and G4 groups were compared in subsequent analyses.

**Fig 2:**
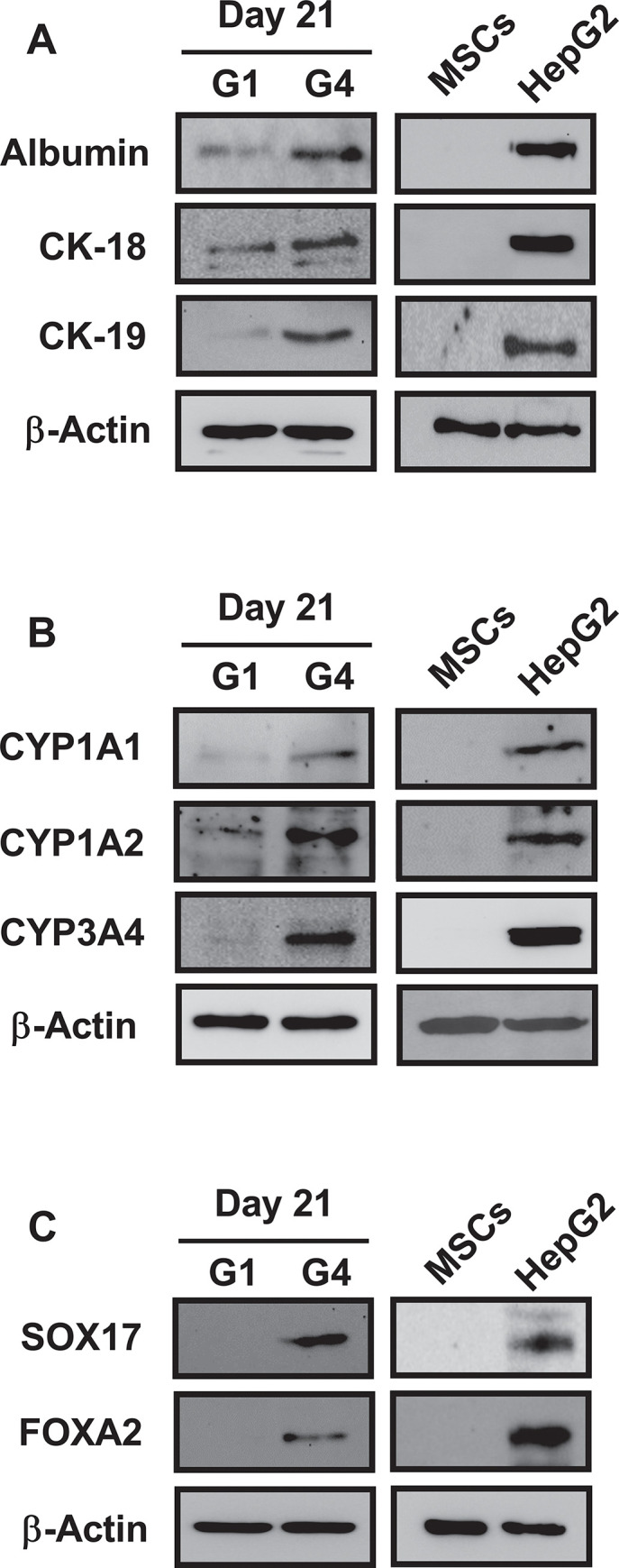
RhLIGHT enhanced the expression of hepatocyte-specific marker proteins in hBM-MSCs. Cells were stimulated with 0 ng/ml and 50 ng/ml rhLIGHT for 21 days. G1 group (control group): hBM-MSCs were incubated with 0 ng/ml rhLIGHT for 21 days. G4 group: hBM-MSCs were incubated with 50 ng/ml rhLIGHT for 21 days. All assays were performed with undifferentiated BM-MSCs as negative controls and HepG2 cells as positive controls. As shown by the immunoblotting analysis, the protein expression of the hepatocyte-specific marker proteins (A) albumin, CK-18, CK-19, (B) CYP1A1, CYP1A2, CYP3A4, (C) SOX17, and FOXA2 was strongly increased in the G4 group compared with the G1 group. The membrane was stripped and reprobed with an anti-β-actin mAb to confirm equal loading.

We sought to evaluate the functional status of hBM-MSC-derived hepatocyte-like cells. For this purpose, hepatocyte-specific marker proteins (e.g., CK-18 and CK-19), which are cytoskeletal proteins expressed in mature hepatocytes, and the expression of their metabolic capacity-related protein CYP450 were investigated using western blotting. As shown in Fig 2A and 2B, the expression of hepatocyte-specific marker proteins (e.g., albumin, CK-18, CK-19, CYP1A1, CYP1A2, and CYP3A4), was significantly increased in the G4 group versus the G1 group. In addition, the levels of the final endoderm expression marker proteins SOX17 and FOXA2 were significantly increased under the same conditions (Fig 2C). These results demonstrate that rhLIGHT enhances the expression of hepatocyte-specific marker proteins (albumin, CK-18, CK-19, CYP1A1, CYP1A2, CYP3A4) and that of definitive endoderm-specific marker proteins (SOX17, FOXA2) to promote the differentiation of hBM-MSCs into hepatocyte-like cells. As shown in Fig 2, our results indicated that the differentiated cells expressed hepatocyte-specific marker proteins and might possess biological activity similar to that of human hepatocyte cell lines, namely HepG2 cells.

### LIGHT enhanced the differentiation of hBM-MSCs into functional hepatocyte-like cells

To further characterize the hBM-MSC-derived hepatocyte-like cells, we assessed indocyanine green uptake and glycogen storage by these cells.

**Fig 3:**
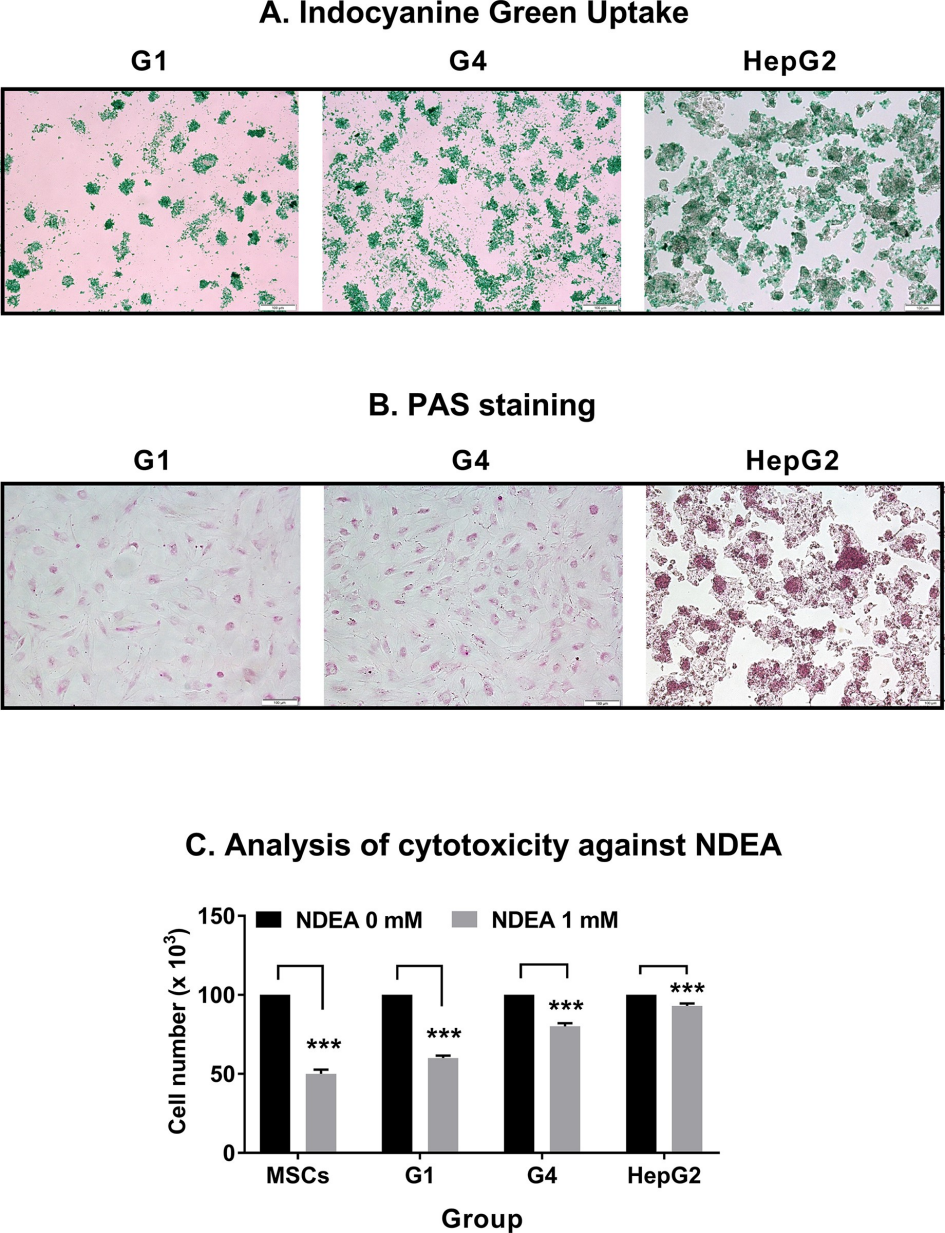
Functional analysis of hepatocytes-like cells derived from rhLIGHT-induced hBM-MSCs *in vitro*. RhLIGHT enhanced the differentiation of hBM-MSCs into functional hepatocyte-like cells. Cells were stimulated with 0 ng/ml and 50 ng/ml rhLIGHT for 21 days (G1 and G4 groups, respectively). (A) Differentiated hBM-MSCs by rhLIGHT exhibited positivity for ICG after incubation in ICG solution for 30 min. Microscope magnification: ×100, Scale bar: 100 μm. (B) PAS staining for glycogen showed that differentiated hBM-MSCs by rhLIGHT could store glycogen after hepatogenic induction for 21 days. Microscope magnification: ×100, Scale bar: 100 μm. (C) Analysis of the cytotoxicity of N-Nitrosodiethylamine (NDEA), a potent hepatocarcinogenic dialkylnitrosoamine, as described in the Materials and Methods. Undifferentiated BM-MSCs and HepG2 were used as negative and positive control, respectively. Data represent the mean ± SEM. *Significantly different from control cells; ***, *P* < 0.001.

The indocyanine green uptake assay, which indicates the mature hepatocytes, was also used to examine the hBM-MSC-derived hepatocyte-like cells generated *in vitro*. In this study, the purpose of the indocyanine green uptake assay was to evaluate the hepatocyte functional activity of hBM-MSC-derived hepatocyte-like cells. The most important function of hepatocytes is detoxification. Indocyanine green is a type of fluorescent dye that is known to be metabolized in microsomes in hepatocytes and then released. Indocyanine green toxicity is low and metabolized only in the liver, so it is widely used clinically for liver function evaluation. In the indocyanine green uptake test, when hepatocytes are functionally active, cellular uptake of indocyanine green occurs, and green fluorescent dye is confirmed when observed under a microscope, and hepatocyte function can be evaluated by the degree of release after metabolism. Our interest is that the bone marrow-derived hepatocyte-like cells have a liver detoxification function and functional activity was observed. In particular, as shown in Fig 3A, intracellular uptake of indocyanine green occurred, and when observed under a microscope, it was confirmed that the green fluorescent dye in which cellular uptake occurred. As shown in Fig 3A, most differentiated cells in both G1 and G4 groups were positive for indocyanine green after incubation for 30 min. We showed relative intensity of indocyanine green uptake of Fig 3A in S3A Fig in S1 File. The relative intensity of indocyanine green uptake in the G4 group is 16.5 times higher than that in the G1 group.

To further examine glycogen storage by hBM-MSC-derived hepatocyte-like cells, the presence of stored glycogen was determined using the PAS staining. As shown in Fig 3B, most differentiated cells in both G1 and G4 groups were positive for PAS staining. We summarized relative intensity glycogen by PAS staining of Fig 3B in S3B Fig in S1 File. The relative intensity of glycogen by PAS staining in the G4 group is 3.25 times higher than that in the G1 group. In the negative control, undifferentiated BM-MSCs, indocyanine green uptake and glycogen production was not observed (data not shown).

To further characterize the hBM-MSC-derived hepatocyte-like cells, we conducted an analysis of cytotoxicity against N-Nitrosodiethylamine (NDEA), a potent hepatocarcinogenic dialkyl-nitrosoamine. NDEA is mainly present in tobacco smoke, water, cheddar cheese, cured meats, fried meals, and many alcoholic beverages. When hepatocellular cells (HepG2) or BM-MSCs bone marrow-derived mesenchymal cells are differentiated into hepatocyte-like cells, they can excrete hepatocarcinogenic agents. Consequently, this process reduces the relative toxicity of such compounds. We used these characteristics to evaluate the differentiation level of hBM-MSC-derived hepatocyte-like cells. As expected, the toxicity of NDEA was lower in the G4 group versus the G1 group. The groups in which NDEA induced the lowest and highest toxicity were HepG2 and MSC groups, respectively. Therefore, it was found that the differentiation into hBM-MSC-derived hepatocyte-like cells was promoted in the G4 group treated with rhLIGHT than in the G1 group.

### Interaction between LIGHT and LTβR increased survival and proliferation in hBM-MSC-derived hepatocyte-like cells

In our previous study, we demonstrated that the interaction between rhLIGHT and LTβR increased the number, viability, proliferation of BM-MSCs, as well as the levels of diverse survival proteins in these cells [20]. Therefore, we sought to confirm the effect of rhLIGHT on the number, survival, proliferation, and cell cycle distribution of hBM-MSC-derived hepatocyte-like cells.

**Fig 4:**
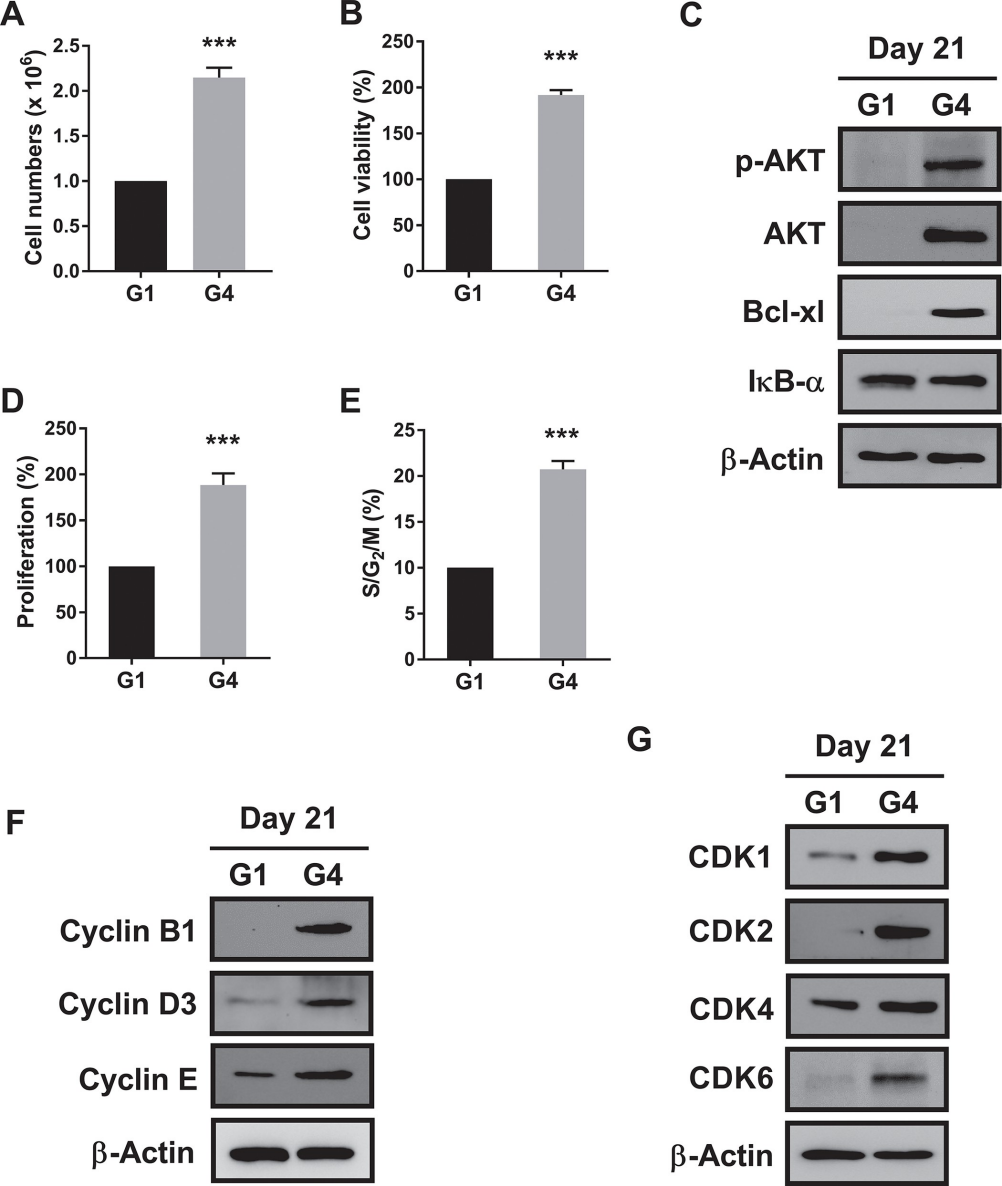
Characterization of hepatocyte-like cells derived from hBM-MSCs after treatment with rhLIGHT *in vitro*. Cells were stimulated with 0 ng/ml and 50 ng/ml rhLIGHT for 21 days (G1 and G4 groups, respectively). Subsequently, we investigated the properties of hepatocyte-like cells derived from hBM-MSCs after treatment with rhLIGHT *in vitro*. Our results showed that rhLIGHT enhanced the differentiation of hBM-MSCs into functional hepatocyte-like cells. (A) Viable cells were counted through trypan blue exclusion assay in the G1 and G4 groups. (B) Cell viability in the G1 and G4 groups, as determined by the MTS assay. (C) Expression of survival proteins and anti-apoptotic proteins in the G1 and G4 groups, as determined by western blotting analysis. (D) Cell proliferation in the G1 and G4 groups, as determined by BrdU assay. (E) Cell cycle distribution in the G1 and G4 groups, as determined by the PI/RNase assay. (F, G) Expression of cell cycle-related proteins in the G1 and G4 groups, as determined by western blotting analysis. The membrane was stripped and reprobed with an anti-β-actin mAb to confirm equal loading. Data represent the mean ± SEM. *Significantly different from control cells; ***, *P* < 0.001.

Initially, as shown in Fig 4A, the results of the trypan blue exclusion analysis revealed that the number of cells in the G4 group had approximately doubled compared with that counted in the G1 group. The cell viability test was performed under the same conditions as above, yielding similar results (Fig 4B). Next, we tested the effects of rhLIGHT on diverse survival proteins, such as phospho-AKT, AKT, Bcl-xl, and nuclear factor-κB (NF-κB) in rhLIGHT-induced hepatocyte-like cells. Briefly, cell viability and the expression of phospho-AKT, AKT, and Bcl-xl was significantly increased after treatment of hBM-MSC-derived hepatocyte-like cells with rhLIGHT (Fig 4C, G4 group). Moreover, rhLIGHT-induced IκB-α degradation activated NF-κB signaling in these cells (Fig 4C).

Additionally, proliferation and cell cycle distribution assays were performed under the same conditions as above, producing consistent results (Fig 4D and 4E). Thus, rhLIGHT increased cell proliferation by increasing the S/G2/M phase in hBM-MSC-derived hepatocyte-like cells (Fig 4D and 4E, G4 group). The levels of cell cycle-related proteins (e.g., cyclin B1, cyclin D3, and cyclin E, and CDK1, CDK2, CDK4, and CDK6) were enhanced by rhLIGHT in hBM-MSC-derived hepatocyte-like cells (Fig 4G, G4 group). Consequently, these results indicated that LIGHT enhances cell survival and proliferation in rhLIGHT-induced hepatocyte-like cells via LTβR (Fig 4, G4 group).

### LIGHT activated the STAT3 and STAT5 pathways in hBM-MSC-derived hepatocyte-like cells

**Fig 5:**
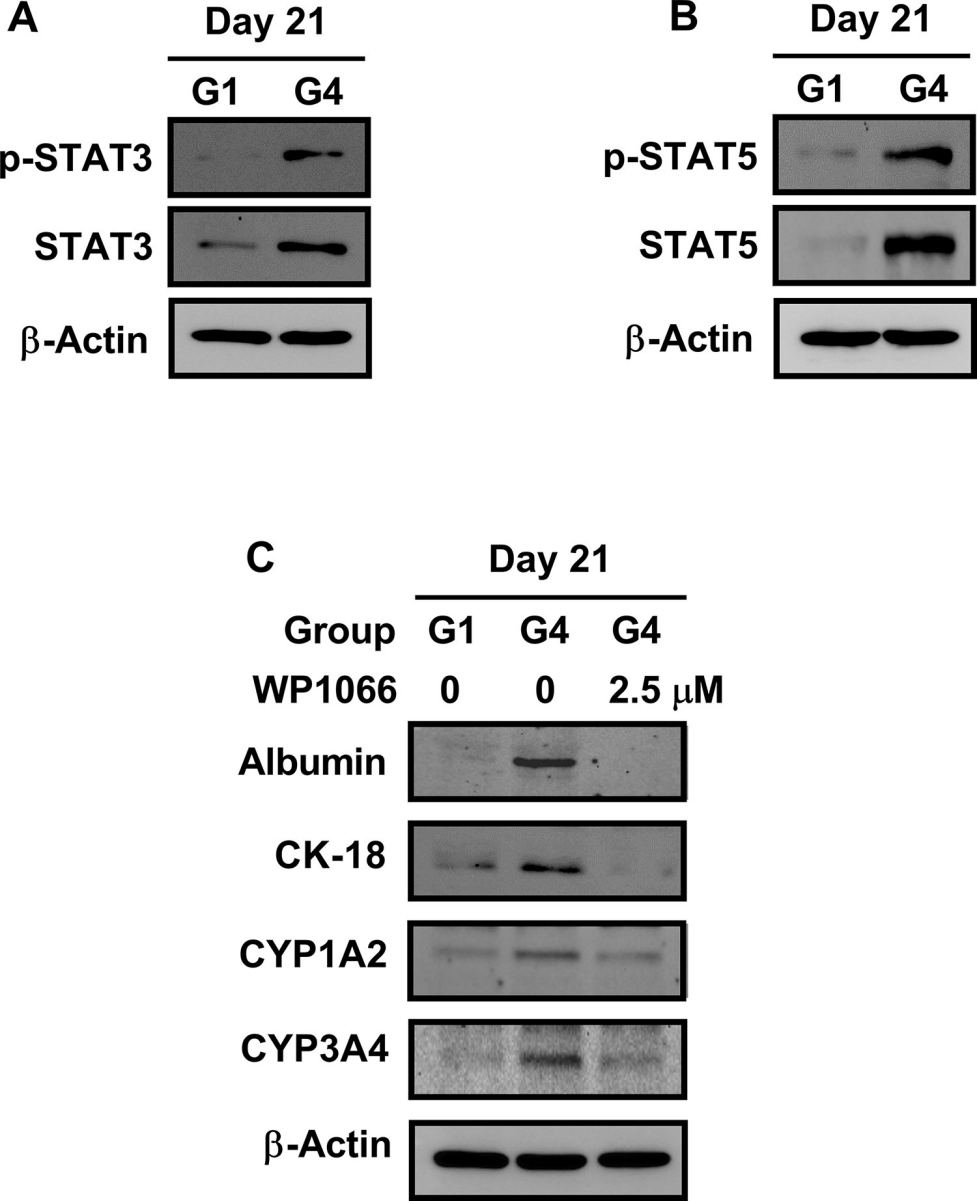
RhLIGHT significantly enhanced the differentiation of hBM-MSCs into functional hepatocyte-like cells by stimulating the activity of STAT3 and STAT5 signaling. Cells were stimulated with 0 ng/ml and 50 ng/ml rhLIGHT for 21 days (G1 and G4 groups, respectively). Thereafter, we investigated the intracellular signaling of hepatocyte-like cells derived from hBM-MSCs after treatment with rhLIGHT *in vitro*. (A, B) Expression of phosphor-STAT3 (p-STAT3), STAT3, p-STAT5, and STAT5 in the G1 and G4 groups, as determined by western blotting analysis. (C) hBM-MSCs were incubated with 0 ng/ml and 50 ng/ml rhLIGHT in the presence or absence of WP1066 (2.5 μM) for 21 days and were harvested. Subsequently, the expression of hepatocyte-specific marker proteins (albumin, CK-18, CYP1A2, and CYP3A4) in cells was analyzed through immunoblotting analysis. The membrane was stripped and reprobed with an anti-β-actin mAb to confirm equal loading.

The diverse role of STAT3 in liver regeneration (i.e., survival, proliferation, DNA synthesis, inflammatory response, and liver mass recovery) has been demonstrated [37]. Okumura et al. showed that STAT5 activation is required for the differentiation of human-induced pluripotent stem cells (PSCs) into hepatocytes. Additionally, these findings suggest that sustained activation of STAT5 is important for the maturation of hepatocytes [38]. Based on these results, we investigated whether STAT3 and STAT5 signaling pathways are involved in the differentiation and division of hBM-MSC-derived hepatocyte-like cells after treatment with rhLIGHT. The findings revealed that STAT3 and STAT5 were strongly activated in the differentiation and division of hepatocyte-like cells following stimulation by LIGHT (Fig 5A and 5B).

To confirm these signaling pathways, hBM-MSC-derived hepatocyte-like cells were pretreated with STAT3 and STAT5 inhibitor WP1066 for 1 h and stimulated with rhLIGHT. Subsequently, the expression of albumin, CK-18, CYP1A2, and CYP3A4 proteins was analyzed by western blotting. In hBM-MSC-derived hepatocyte-like cells pretreated with STAT3 and STAT5 inhibitor WP1066, the expression of these proteins induced by rhLIGHT, including albumin, was significantly reduced (Fig 5C). These results suggest that STAT3 and STAT5 signaling play an important role in the differentiation and division of hBM-MSC-derived hepatocyte-like cells. Therefore, according to the findings, the rhLIGHT-induced hepatogenic differentiation of hBM-MSCs was mediated by activation of the STAT3 and STAT5 pathways (Fig 5).

### LIGHT enhanced the expression of LTβR in hBM-MSC-derived hepatocyte-like cells

**Fig 6:**
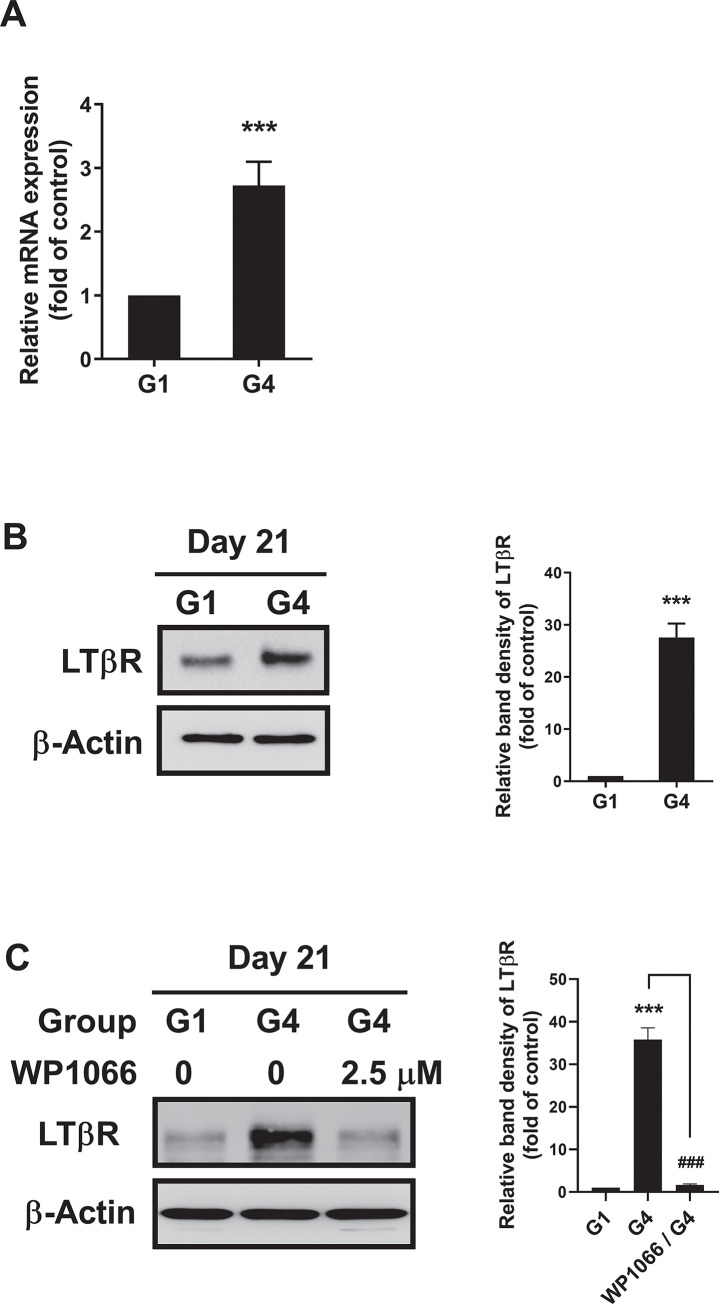
Treatment with rhLIGHT strongly enhanced the differentiation of hBM-MSCs into functional hepatocyte-like cells by inducing the expression of LIGHT receptor LTβR. (A) As revealed by qRT-PCR analysis, after stimulation of cells with 0 ng/ml and 50 ng/ml rhLIGHT for 21 days (G1 and G4 groups, respectively), the mRNA expression of LTβR was increased in the latter group versus the former group. (B) Cells were also collected and treated under the same conditions described in (A). According to the results of the immunoblotting analysis, the protein expression of LTβR was increased after 21 days in a dose-dependent manner. (C) hBM-MSCs were incubated with 0 ng/ml and 50 ng/ml rhLIGHT in the presence or absence of WP1066 (2.5 μM) for 21 days and were harvested. Subsequently, the expression of LTβR in cells was analyzed through immunoblotting analysis. The membrane was stripped and reprobed with an anti-β-actin mAb to confirm equal loading. Data represent the mean ± SEM. Significantly different from control (*) or G4 group (#); ***, ###: *P* < 0.001.

We investigated the effects of rhLIGHT on the expression of its receptor LTβR in hBM-MSCs. Our results showed that LTβR was constitutively expressed on the membrane of hBM-MSCs, as demonstrated in S1C Fig in S1 File. Additionally, the expression of LTβR was altered in hBM-MSC-derived hepatocyte-like cells after treatment with rhLIGHT. Specifically, the expression of LTβR mRNA and protein was 2.7- and 23-fold higher, respectively, in the G4 group treated with rhLIGHT versus the control group (G1 group), as determined by qRT-PCR and western blotting analyses (Fig 6A and 6B, respectively). These findings suggested that LIGHT enhances the expression of its receptor LTβR in hBM-MSC-derived hepatocyte-like cells.

Moreover, experiments were performed to investigate the relevance of LIGHT-induced LTβR expression in the differentiation process. After treatment with STAT3 and STAT5 inhibitor WP1066, the level of induction of LTβR by rhLIGHT was investigated. As a result, the LTβR expression induced by rhLIGHT was significantly reduced (Fig 6C). These results suggest that STAT3 and STAT5 signaling play an essential role in the LTβR expression, and differentiation of hBM-MSC-derived hepatocyte-like cells.

## Discussion

The prevalence of liver disease is increasing globally, leading to acute and chronic liver damage, which negatively impacts human health. Liver transplantation is currently the most effective treatment for patients with liver disease [39]. As part of ongoing efforts to improve treatment options, a clinical trial involving allogeneic human hepatocyte transplantation for patients with liver cirrhosis is currently underway in China (Clinical Study of Liver Cell Transplantation for Cirrhosis [National Clinical Trials number: NCT04806581; www.clinicaltrials.gov/ct2/show/NCT04806581]). The primary objectives of this study were to observe and determine the safety and tolerability of allogeneic hepatocyte transplantation in patients with liver cirrhosis, establish the maximum tolerated dose, and evaluate dose-limiting toxicity. A secondary objective was to evaluate the therapeutic efficacy of allogeneic hepatocyte transplantation for liver cirrhosis. However, the identification of a suitable donor for liver transplantation within a limited timeframe can be difficult. In such challenging treatment situations, transplantation of hepatocyte-like cells could be a viable option. Therefore, practical studies on the differentiation of hepatocyte-like cells using various types of stem cells are necessary.

Hepatocyte-like cells can be generated through differentiation from various human PSCs or multipotent stem cells [40]. These include induced PSCs, embryonic stem cells, and multipotent stem cells, such as cord blood stem cells or MSCs obtained from adults. Among these, MSCs are typically isolated from bone marrow (BM-MSCs) [11]. Thus, BM-MSCs were used in this study. Furthermore, MSCs can differentiate into tissues of mesodermal origin, including bone, cartilage, and fat, as well as generate neurons and hepatocytes.

LIGHT (TNFSF14) has three receptors, namely LTβR (TNFRSF3), herpesvirus entry mediator (TNFRSF14, CD270, HVEM), and decoy receptor-3 (DcR3, TNFRSF6B) [26, 27]. Among these, HVEM, another membrane-bound receptor for LIGHT, is expressed in endothelial and immune cells, including dendritic, natural killer, T, and B cells [30, 41]. The interaction between LIGHT and HVEM plays a vital role in immune cell defense against foreign antigens [26]. Upon interaction, LIGHT and HVEM costimulate T cell proliferation and cytokine production [42], induce dendritic cell activation and maturation [43], stimulate immunoglobulin secretion in B cells [44], activate natural killer cells [45], and enhance the bactericidal activity of monocytes and neutrophils [41, 46]. In contrast, LTβR is expressed in fibroblasts, endothelial cells, and stromal cells [28–30]. Moreover, it is also constitutively expressed in hBM-MSCs [20, 31]. However, there is limited research on the interaction between LIGHT and LTβR.

In our previous study, we demonstrated that the interaction between LIGHT and LTβR enhances the survival and proliferation of hBM-MSCs [20]. However, the role of this interaction in the hepatogenic differentiation of hBM-MSCs is poorly understood. Therefore, in this study, we aimed to investigate the effect of LIGHT on the differentiation of hBM-MSCs into functional hepatocyte-like cells.

In this study, as depicted in Figs 1 and 2, we demonstrated that LIGHT promotes the expression of hepatocyte-specific marker proteins in hBM-MSCs. Moreover, the interaction between LIGHT and LTβR enhances the differentiation of hBM-MSCs into functional hepatocyte-like cells (Fig 3). We evaluated the functional role of these cells as hepatocyte-like cells by assessing their ICG uptake and glycogen storage functions (Fig 3A and 3B). In the hepatogenic differentiation stage, there is no significant change from the conventional BM-MSCs shape, but the shape changes dramatically when entering the hepatogenic maturation stage. During the two stages, the shape changes to a more rounded shape. Finally, it looks like Fig 3B. As evidenced by the upregulation of several proteins (including various cyclins and CDK proteins), the interaction between LIGHT and LTβR increased the survival and proliferation of hBM-MSC-derived hepatocyte-like cells (Fig 4). Our findings further revealed that LIGHT induces the differentiation of hBM-MSC-derived hepatocyte-like cells through the activation of the STAT3 and STAT5 pathways. Notably, this effect was completely inhibited by treatment with the STAT3 and STAT5 inhibitor WP1066 (Fig 5). Furthermore, we observed that LIGHT accelerated the differentiation process by enhancing the expression of LTβR in hBM-MSC-derived hepatocyte-like cells (Fig 6). Moreover, LIGHT-induced LTβR expression and differentiation of hBM-MSC-derived hepatocyte-like cells are potentiated through STAT3 and STAT5 signaling activation as shown in Fig 6C. In summary, the results of the present study highlight the crucial role of the interaction between LIGHT and LTβR in promoting the differentiation of hBM-MSCs into functional hepatocyte-like cells. According to these findings, LIGHT may have significant implications for stem cell therapy involving the differentiation of MSCs. We found that the differentiation of hBM-MSCs into hepatocyte-like cells occurred as a result of the interaction between LIGHT and LTβR, one of the LIGHT receptors on the cell surface of hBM-MSCs. However, further studies may be needed to determine whether LTβRs are essential for the differentiation of hBM-MSCs into hepatocyte-like cells. Then, experiments with knockdown techniques such as RNA interference via lentivirus would be a good option.

MSCs hold great potential for the treatment of liver fibrosis due to their unique properties and promising results from preclinical and clinical studies. However, in recent years, concerns have arisen regarding the long-term effectiveness and potential tumorigenic risk associated with MSC-based therapies. Additionally, currently, there is no standardized protocol for MSC transplantation [47]. Therefore, further research studies are warranted to assess the long-term safety and efficacy of MSC-based treatments.

The present study demonstrated that the interaction between LIGHT and LTβR promotes the hepatogenic differentiation of hBM-MSCs by activating the STAT3 and STAT5 pathways. This process accelerates the differentiation of hBM-MSCs into functional hepatocyte-like cells. Furthermore, we confirmed that interaction between LIGHT and LTβR contributes to the induction of hBM-MSC-derived liver formation, while increasing the expression of LTβR; these effects enhance the rate of cell differentiation. This evidence may provide practical information and highlight the potential of LIGHT in stem cell therapy, particularly in the context of liver disorders. To our knowledge, this is the first study to investigate the effect of interaction between LIGHT and LTβR on the hepatogenic differentiation of hBM-MSCs. The data indicated that LIGHT is a potent inducing molecule for the differentiation of hBM-MSCs toward hepatocyte-like cells. In summary, according to our results, LIGHT may be a promising target for therapies against liver disorders. However, further studies are required to evaluate the long-term safety and efficacy of MSC-based treatments in clinical settings.

## Supporting information

S1 File (DOCX)

S1 Raw images (PPTX)

S2 Raw images (PPTX)

## Abbreviations

MSCs: mesenchymal stem cells
BMCs: bone marrow cells
BM-MSCs: bone marrow-derived mesenchymal stem cells
LTβR: LT-β receptor
HVEM: herpes virus entry mediator
FGF: fibroblast growth factor
EGF: epidermal growth factor; HGF, hepatocyte growth factor
DMEM-LG: Dulbecco’s Modified Eagle Medium-low glucose
TNF: tumor necrosis factor
CK: cytokeratin
CYP1A1: cytochrome P450 Family 1 Subfamily A Member 1
SOX17: SRY-Box Transcription Factor 17
FOXA2: Forkhead box protein A2
BSA: bovine serum albumin. BrdU, bromo-deoxyuridine
hBM-MSCs: human bone marrow-derived mesenchymal stem cells
mAb: monoclonal antibody
PI: propidium iodide
rhLIGHT: recombinant human LIGHT
ICG: indocyanine green
PAS: periodic acid–Schiff
STAT3: signal transducer and activator of transcription 3
