# Supplementary material for: LIGHT (TNFSF14) promotes the differentiation of human bone marrow-derived mesenchymal stem cells into functional hepatocyte-like cells
Source: PLoS One. 2023 Aug 8;18(8):e0289798. doi: 10.1371/journal.pone.0289798 (PMC10411951; doi:10.1371/journal.pone.0289798)
Supplement: S1 File — (DOCX) [file pone.0289798.s001.docx]

**Supplementary Materials**

**LIGHT (TNFSF14) promotes the differentiation of human bone marrow-derived mesenchymal stem cells into functional hepatocyte-like cells**

**Running title**: Role of LIGHT in the differentiation of hBM-MSCs into hepatocyte-like cells

Sook-Kyoung Heo, Ho-Min Yu, Do Kyoung Kim, Hye Jin Seo, Yerang Shin, Sung Ah Kim, Minhui Kim, Youjin Kim, Yoo Jin Lee, Eui-Kyu Noh, Jae-Cheol Jo

**Composition of hepatogenic differentiation medium**

**Fig. S1.** The composition of the hepatocyte differentiation medium


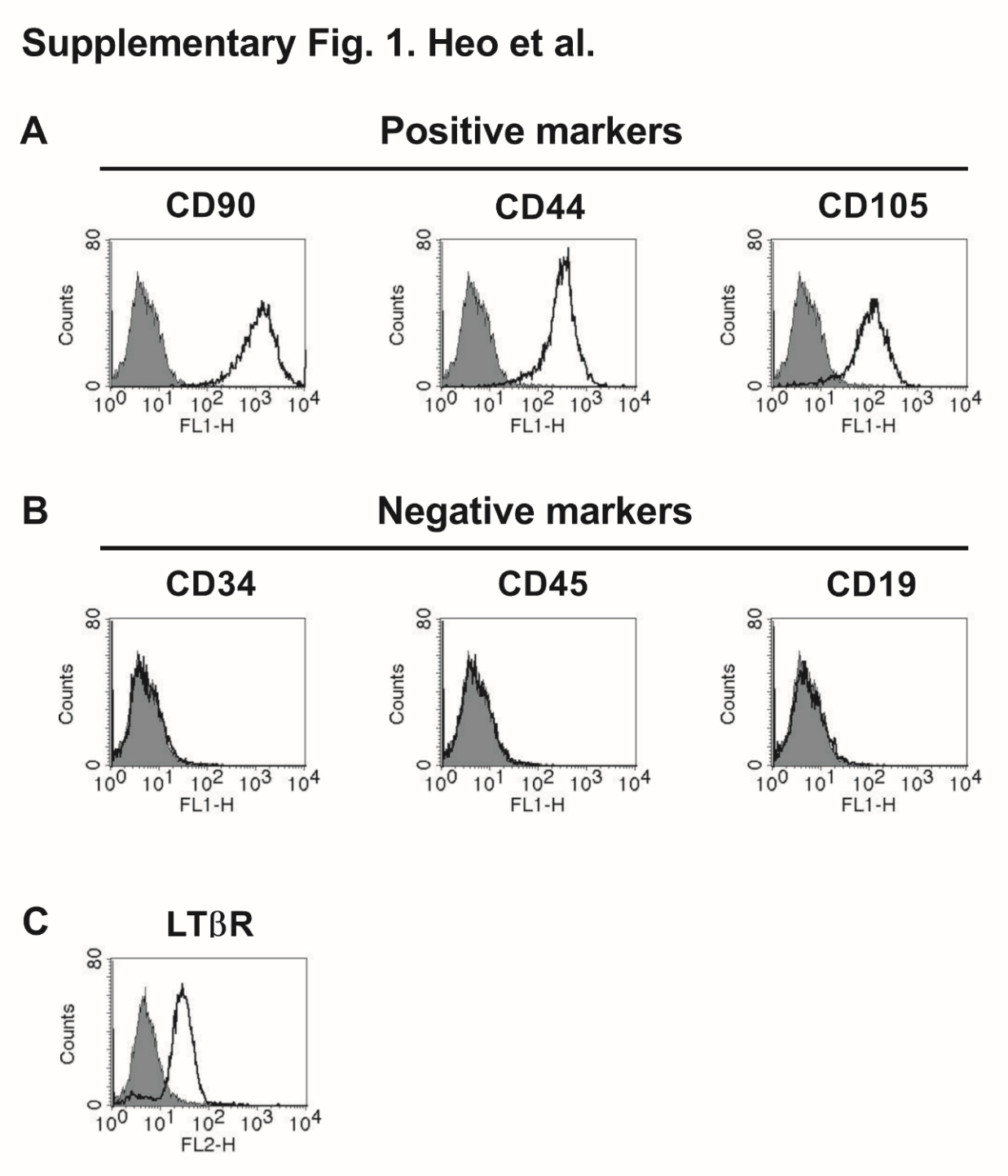


**Fig. S2.** Quality test for hBM-MSCs.

(A) Staining of positive markers (CD90, CD44, and CD105) in BM-MSCs. (B) Staining of negative markers (CD34, CD45, and CD19) in BM-MSCs. (C) LTβR expression on the surface of hBM-MSCs. The expression levels of each marker were determined using FACS analysis. The filled histogram represents the isotype control (mouse IgG); the open histogram represents each antigen.

Abbreviations: BM-MSCs, bone marrow-derived mesenchymal stem cells; FACS, fluorescence-activated cell sorter; hBM-MSCs, human BM-MSCs; IgG, immunoglobulin G; LTβR, lymphotoxin-β receptor.


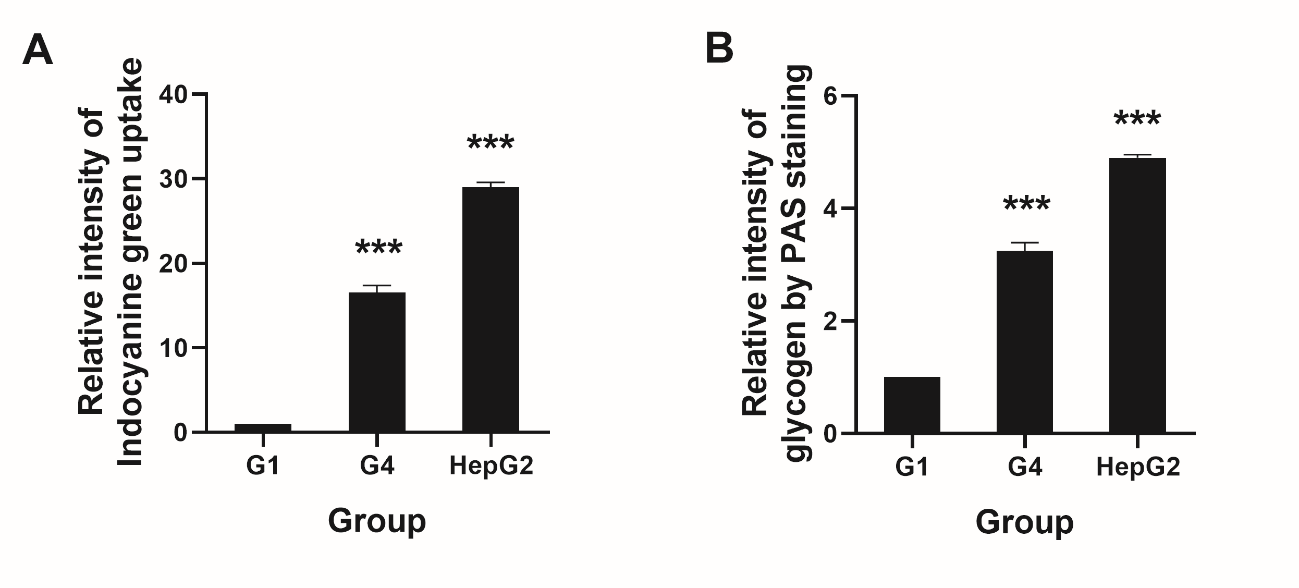


**Fig. S3.** Relative intensity of indocyanine green uptake of Figure 3A, and relative intensity of glycogen by PAS staining of Figure 3B. The relative intensity was measured using the ImageJ program. Data represent the mean ± SEM. *Significantly different from control cells; ***, *P* < 0.001.
