# Supplementary material for: LIGHT (TNFSF14) promotes the differentiation of human bone marrow-derived mesenchymal stem cells into functional hepatocyte-like cells
Source: PLoS One. 2023 Aug 8;18(8):e0289798. doi: 10.1371/journal.pone.0289798 (PMC10411951; doi:10.1371/journal.pone.0289798)
Supplement: S1 Raw images — (PPTX) [file pone.0289798.s002.pptx]

## Slide 1
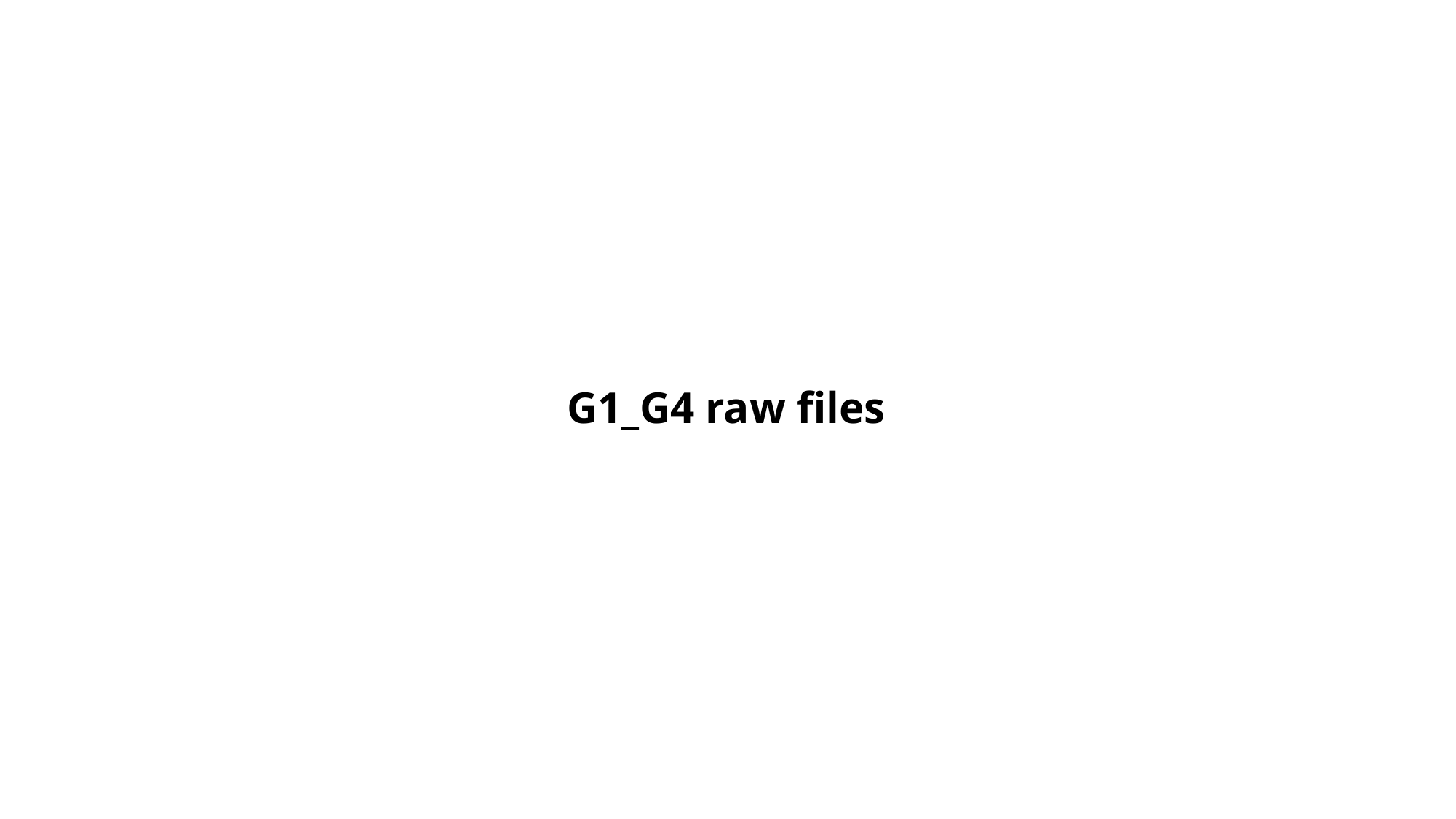

G1_G4 raw files

## Slide 2
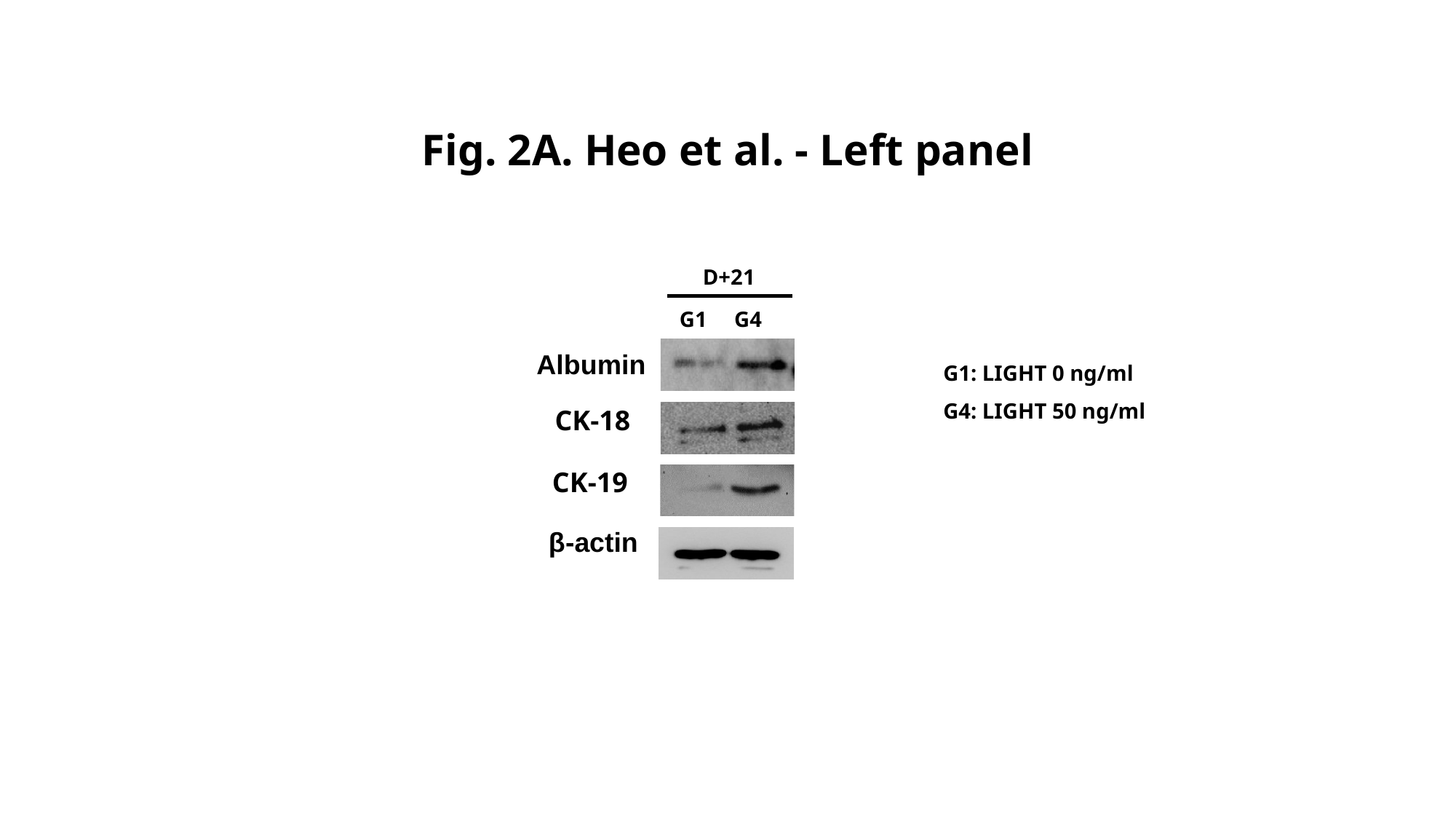

# Fig. 2A. Heo et al. - Left panel
D+21
G1 G4
G1: LIGHT 0 ng/ml
G4: LIGHT 50 ng/ml
Albumin
CK-18
CK-19
β-actin

## Slide 3
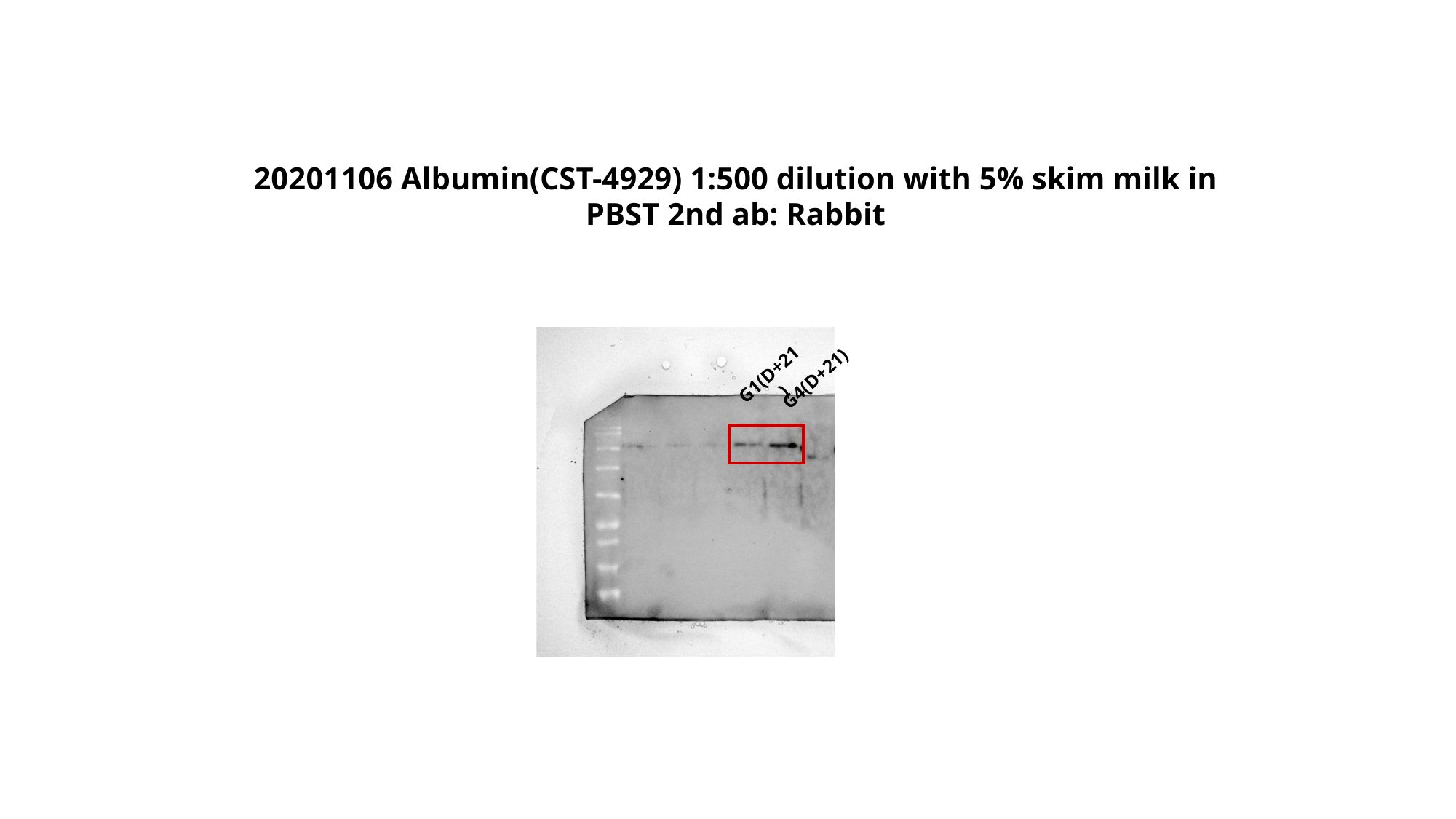

20201106 Albumin(CST-4929) 1:500 dilution with 5% skim milk in PBST 2nd ab: Rabbit
G1(D+21)
G4(D+21)

## Slide 4
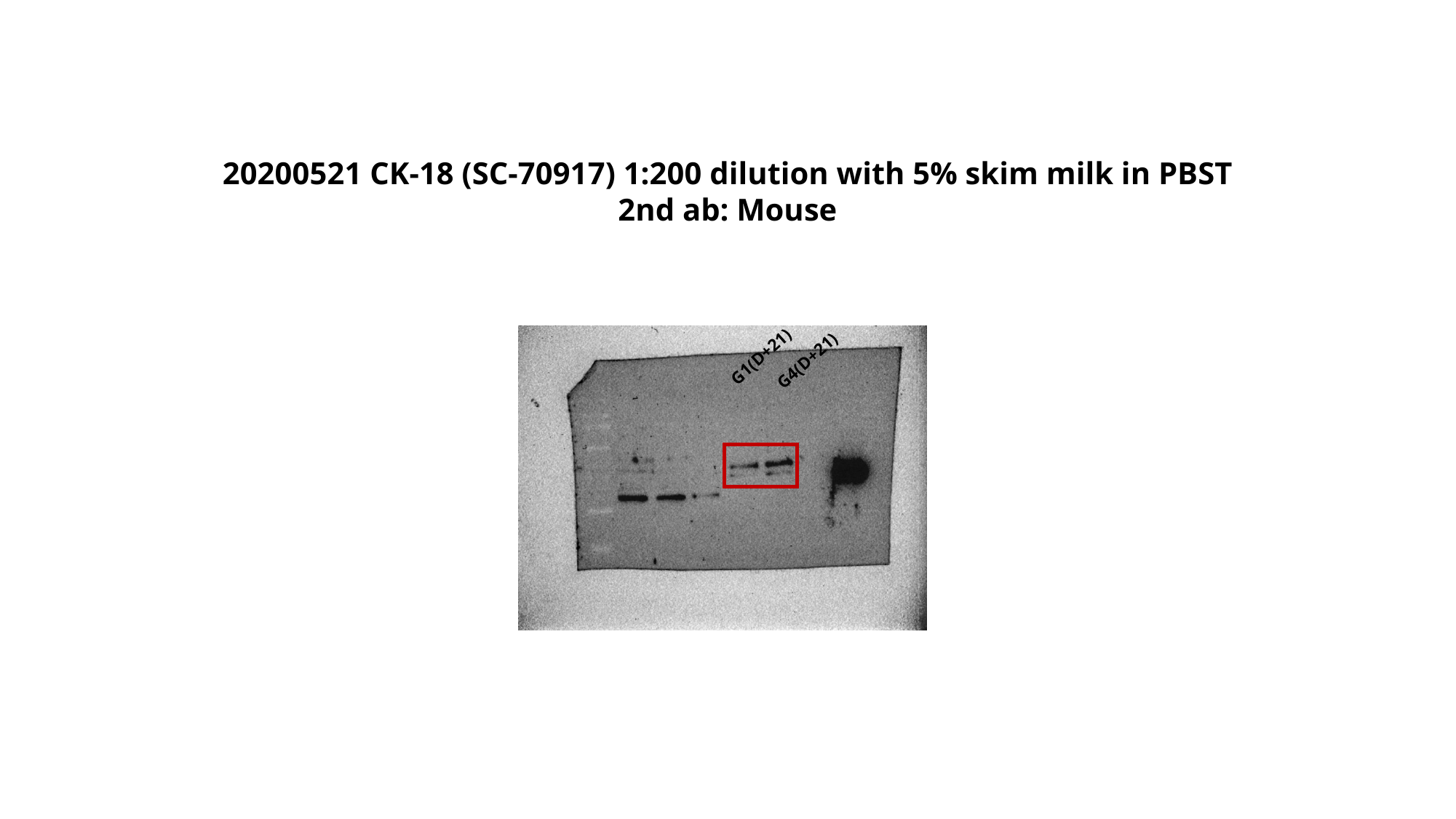

20200521 CK-18 (SC-70917) 1:200 dilution with 5% skim milk in PBST 2nd ab: Mouse
G1(D+21)
G4(D+21)

## Slide 5
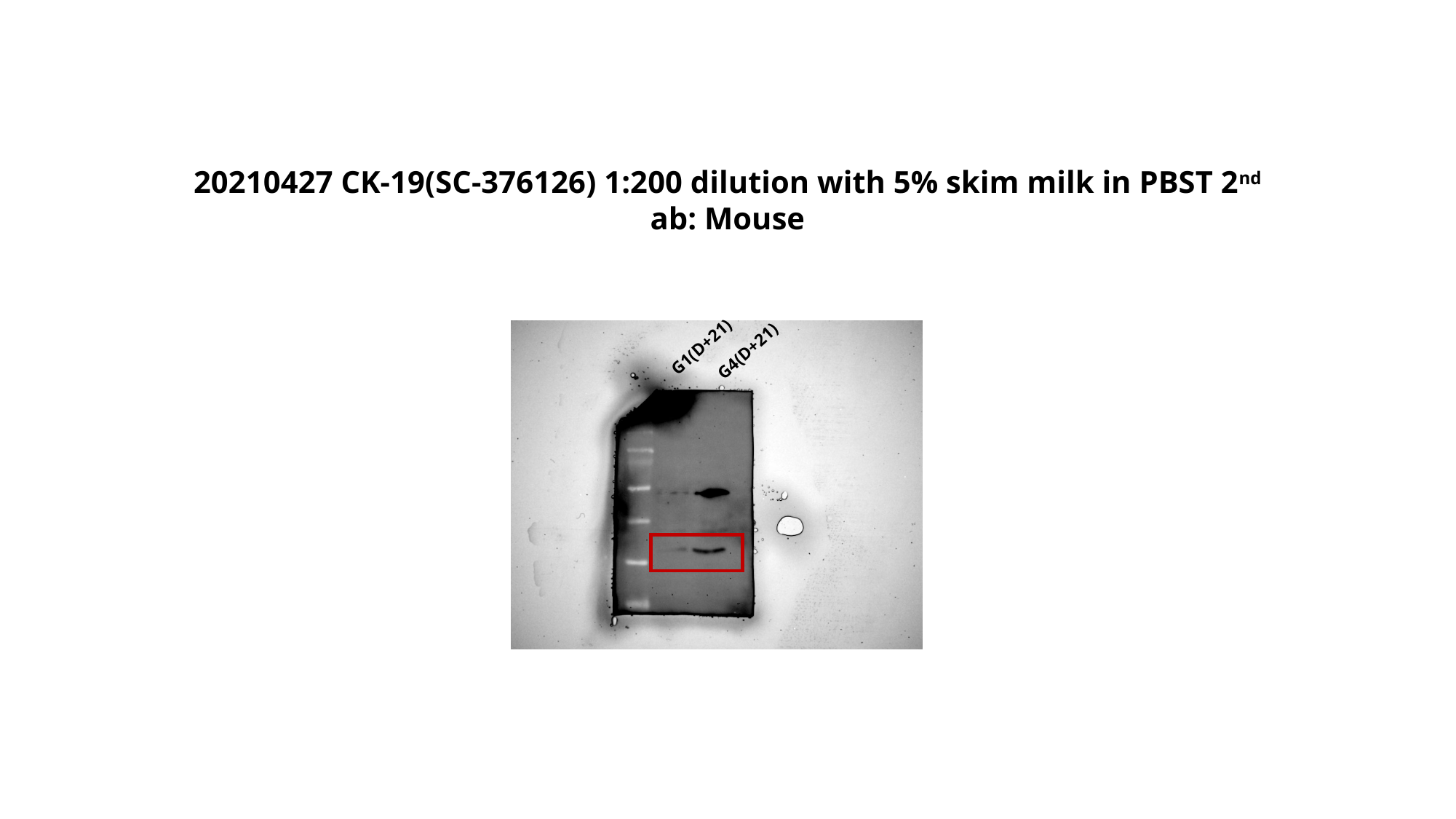

20210427 CK-19(SC-376126) 1:200 dilution with 5% skim milk in PBST 2nd ab: Mouse
G1(D+21)
G4(D+21)

## Slide 6
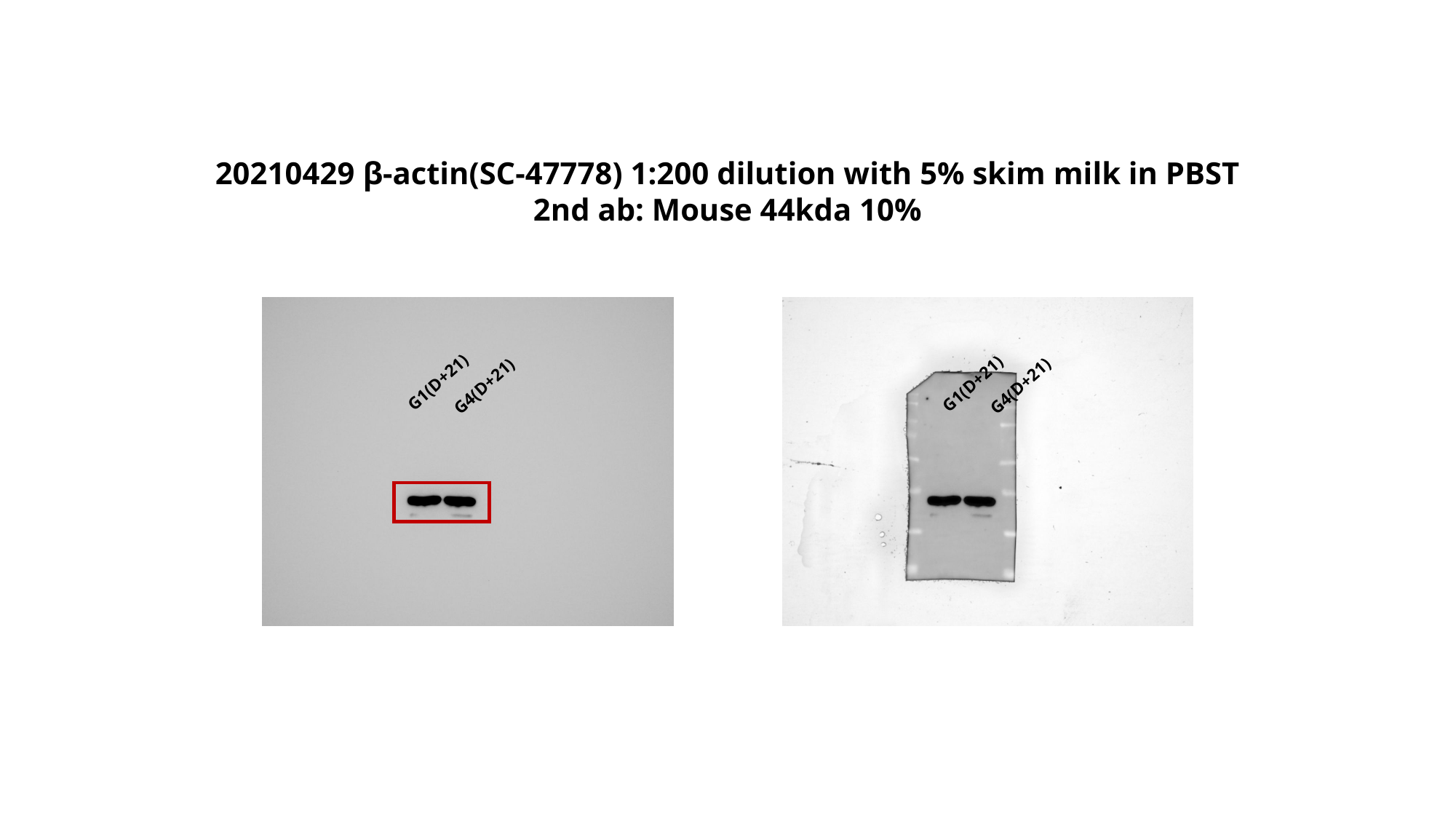

20210429 β-actin(SC-47778) 1:200 dilution with 5% skim milk in PBST 2nd ab: Mouse 44kda 10%
G1(D+21)
G4(D+21)
G1(D+21)
G4(D+21)

## Slide 7
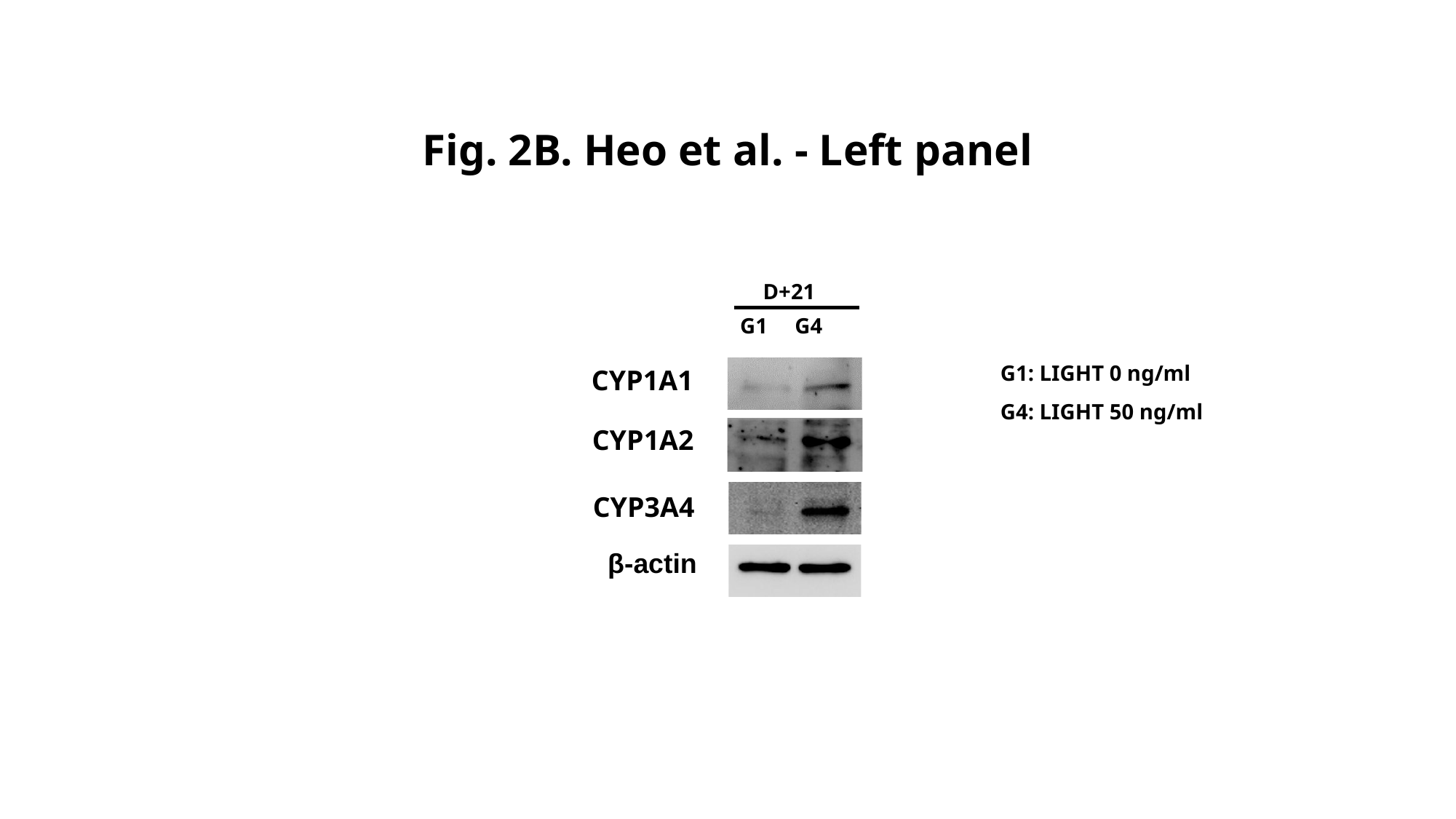

# Fig. 2B. Heo et al. - Left panel
D+21
G1 G4
G1: LIGHT 0 ng/ml
G4: LIGHT 50 ng/ml
CYP1A1
CYP1A2
CYP3A4
β-actin

## Slide 8
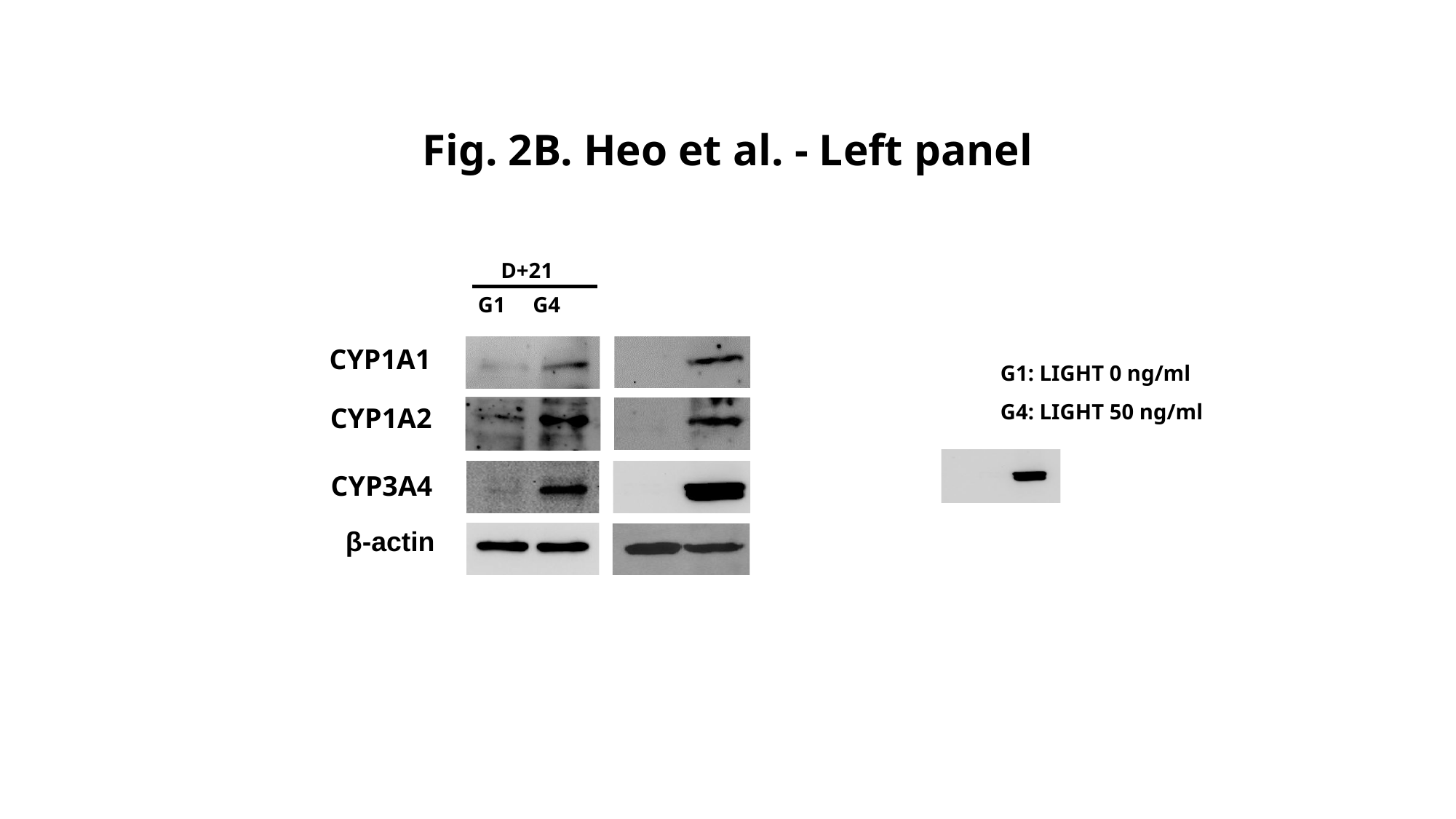

# Fig. 2B. Heo et al. - Left panel
D+21
G1 G4
CYP1A1
G1: LIGHT 0 ng/ml
G4: LIGHT 50 ng/ml
CYP1A2
CYP3A4
β-actin

## Slide 9
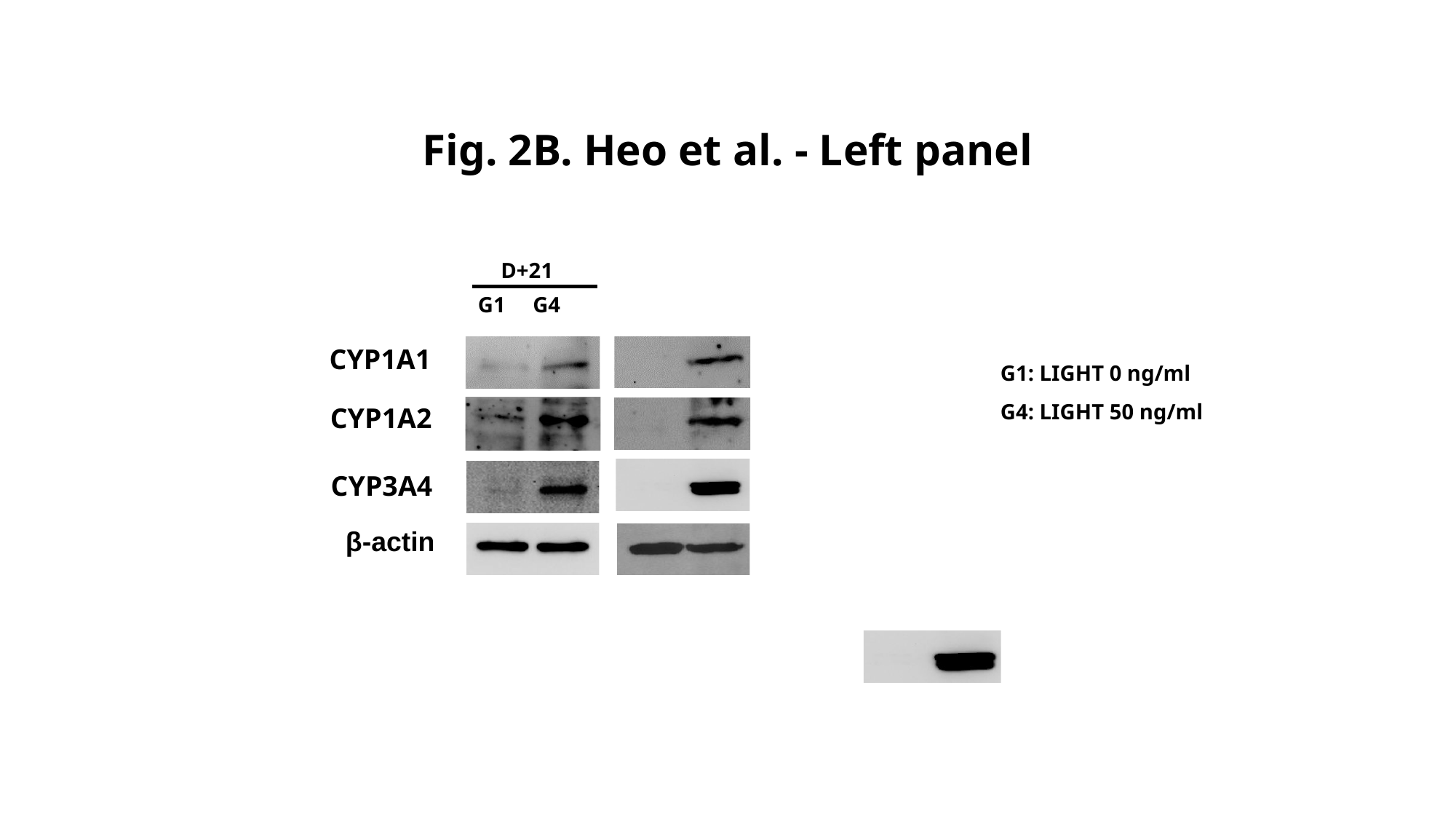

# Fig. 2B. Heo et al. - Left panel
D+21
G1 G4
CYP1A1
G1: LIGHT 0 ng/ml
G4: LIGHT 50 ng/ml
CYP1A2
CYP3A4
β-actin

## Slide 10
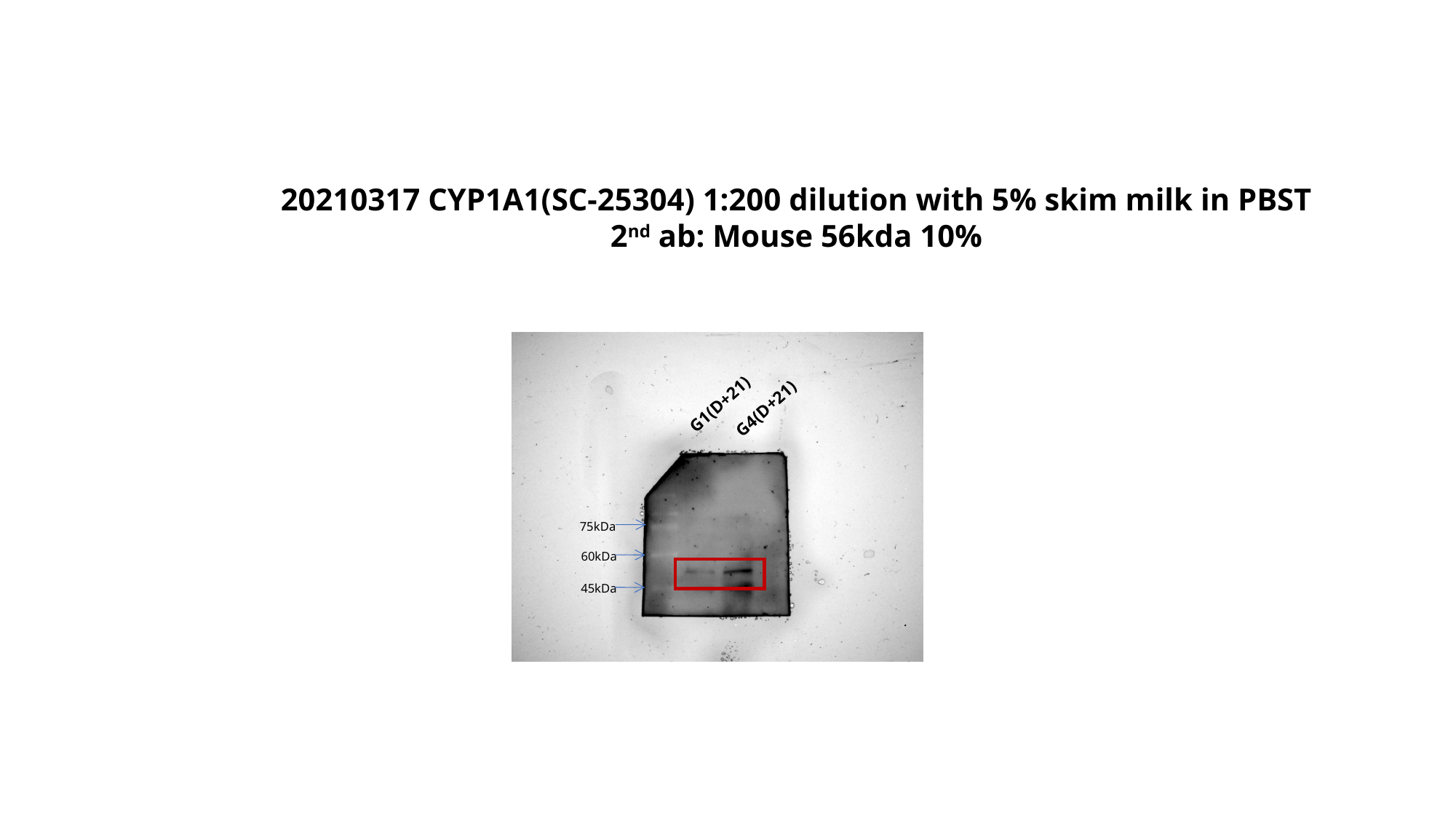

20210317 CYP1A1(SC-25304) 1:200 dilution with 5% skim milk in PBST 2nd ab: Mouse 56kda 10%
G1(D+21)
G4(D+21)
75kDa
60kDa
45kDa

## Slide 11
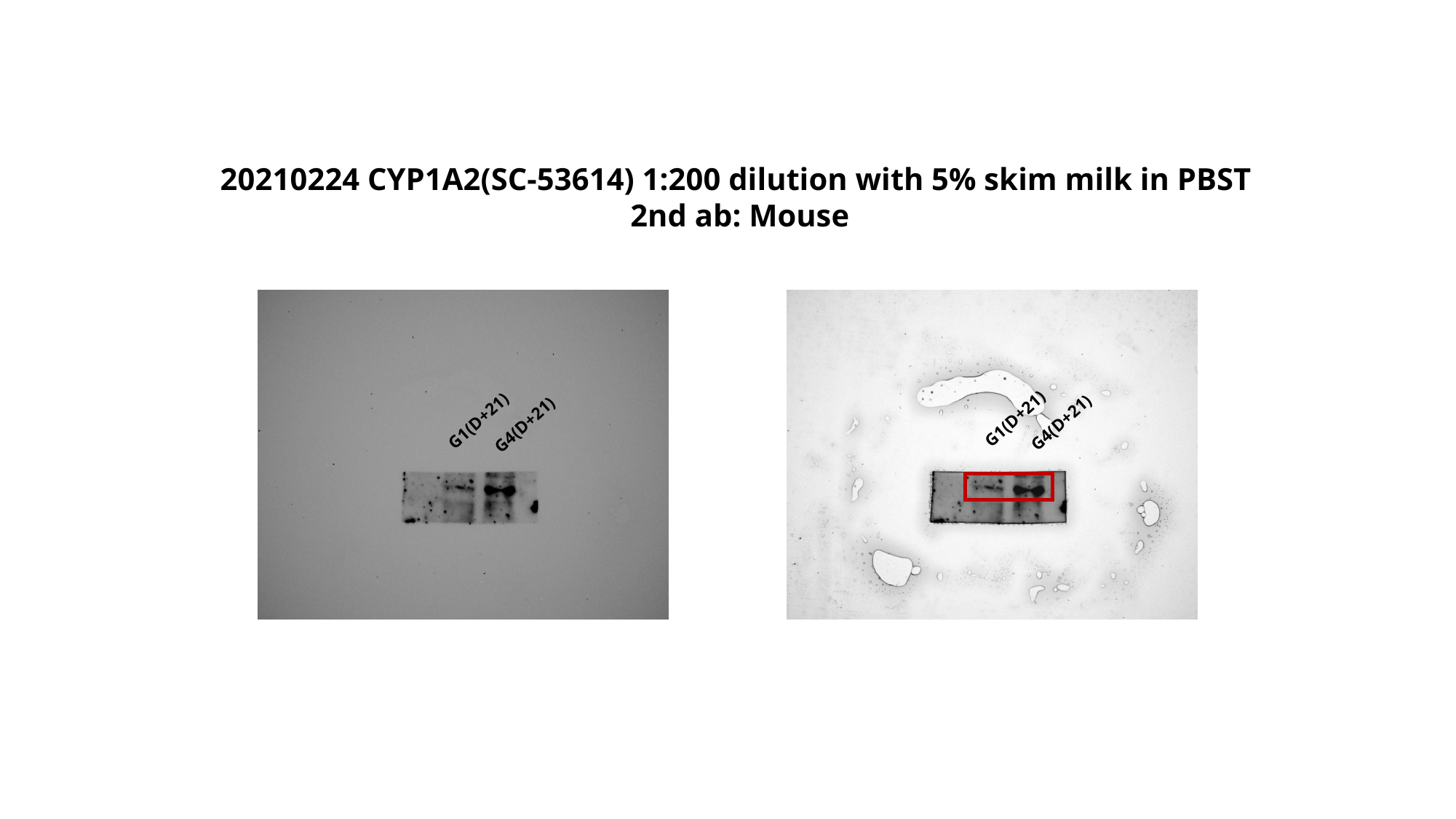

20210224 CYP1A2(SC-53614) 1:200 dilution with 5% skim milk in PBST
2nd ab: Mouse
G1(D+21)
G4(D+21)
G1(D+21)
G4(D+21)

## Slide 12
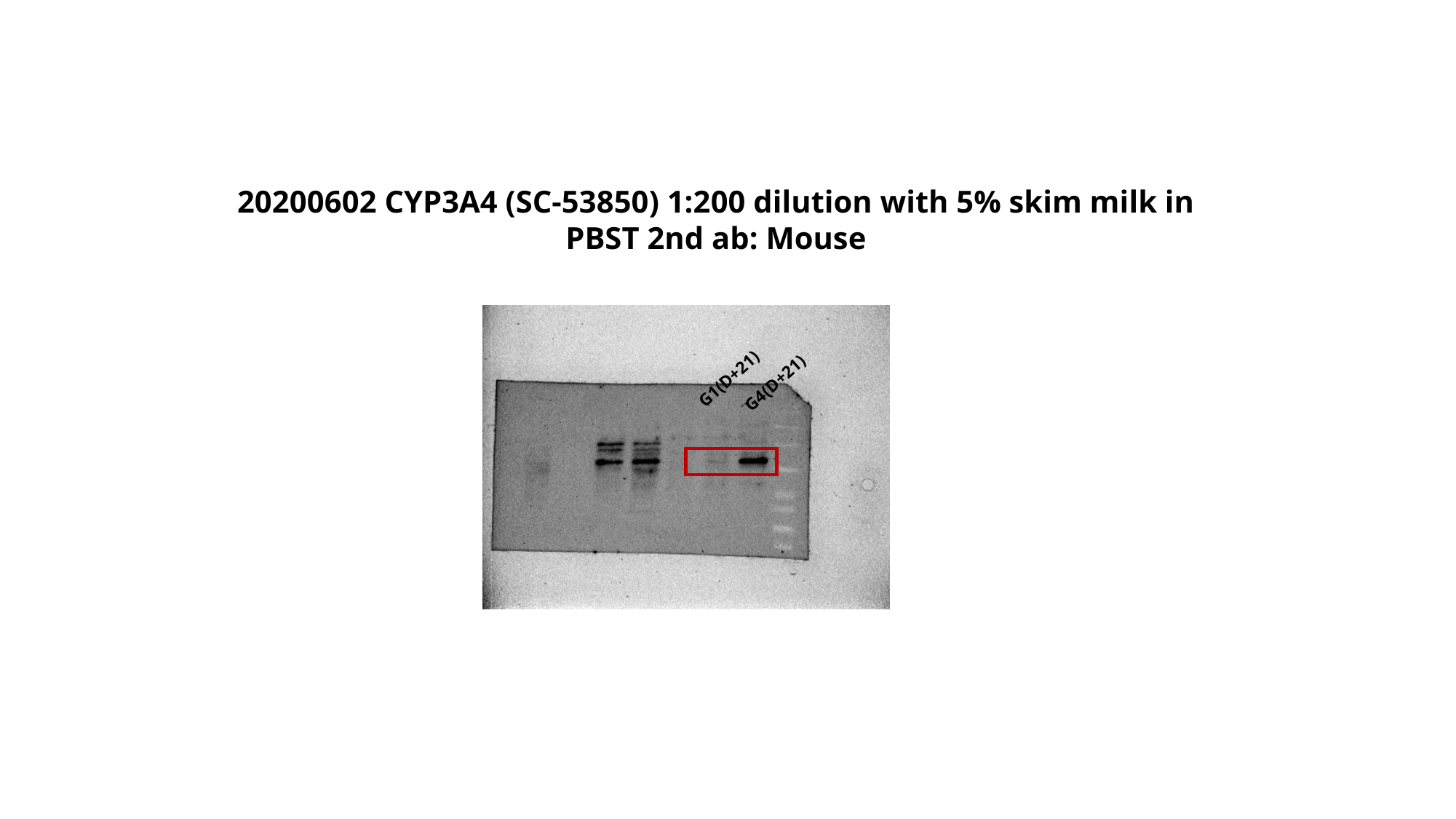

20200602 CYP3A4 (SC-53850) 1:200 dilution with 5% skim milk in PBST 2nd ab: Mouse
G1(D+21)
G4(D+21)

## Slide 13
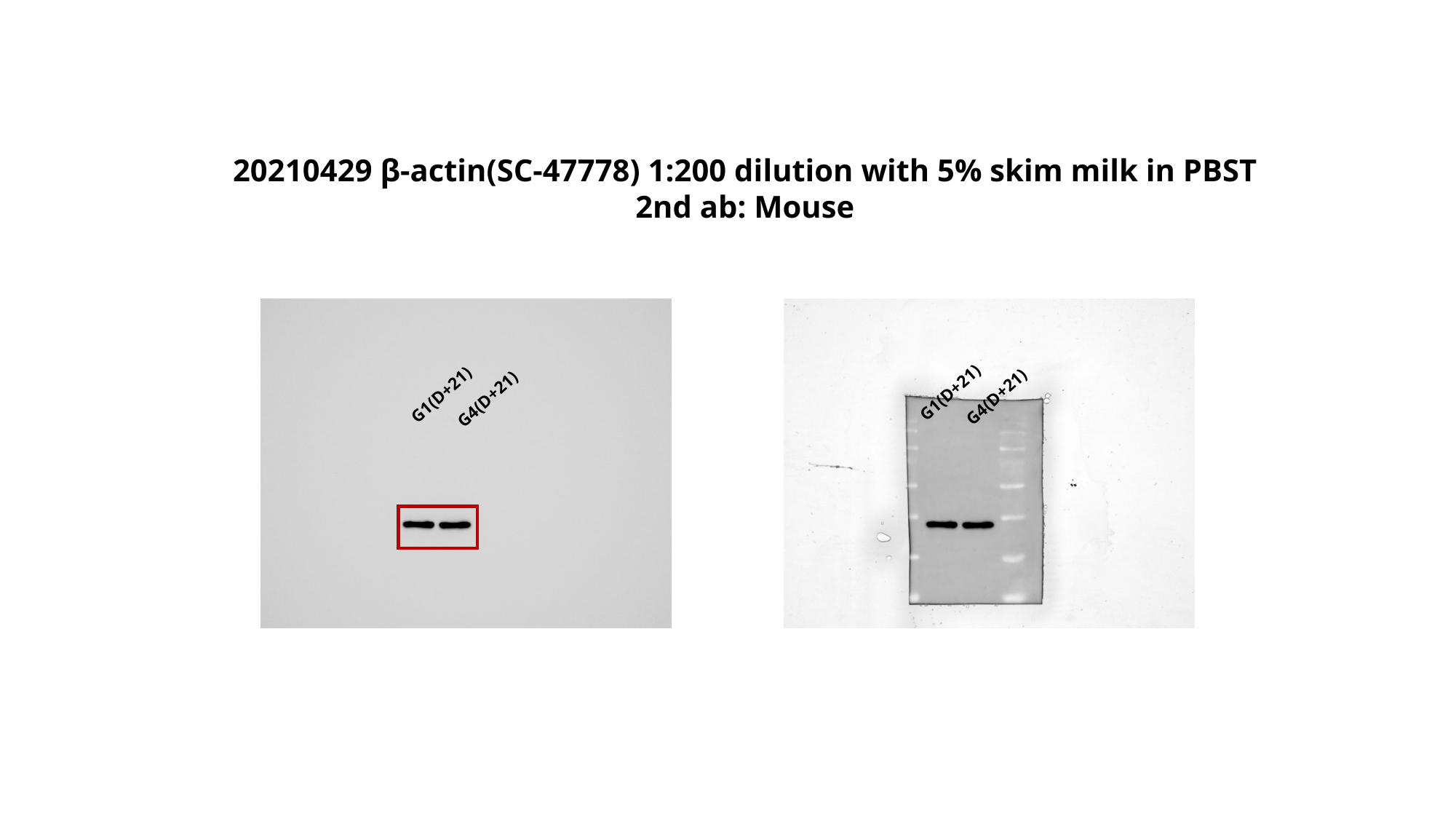

20210429 β-actin(SC-47778) 1:200 dilution with 5% skim milk in PBST 2nd ab: Mouse
G1(D+21)
G4(D+21)
G1(D+21)
G4(D+21)

## Slide 14
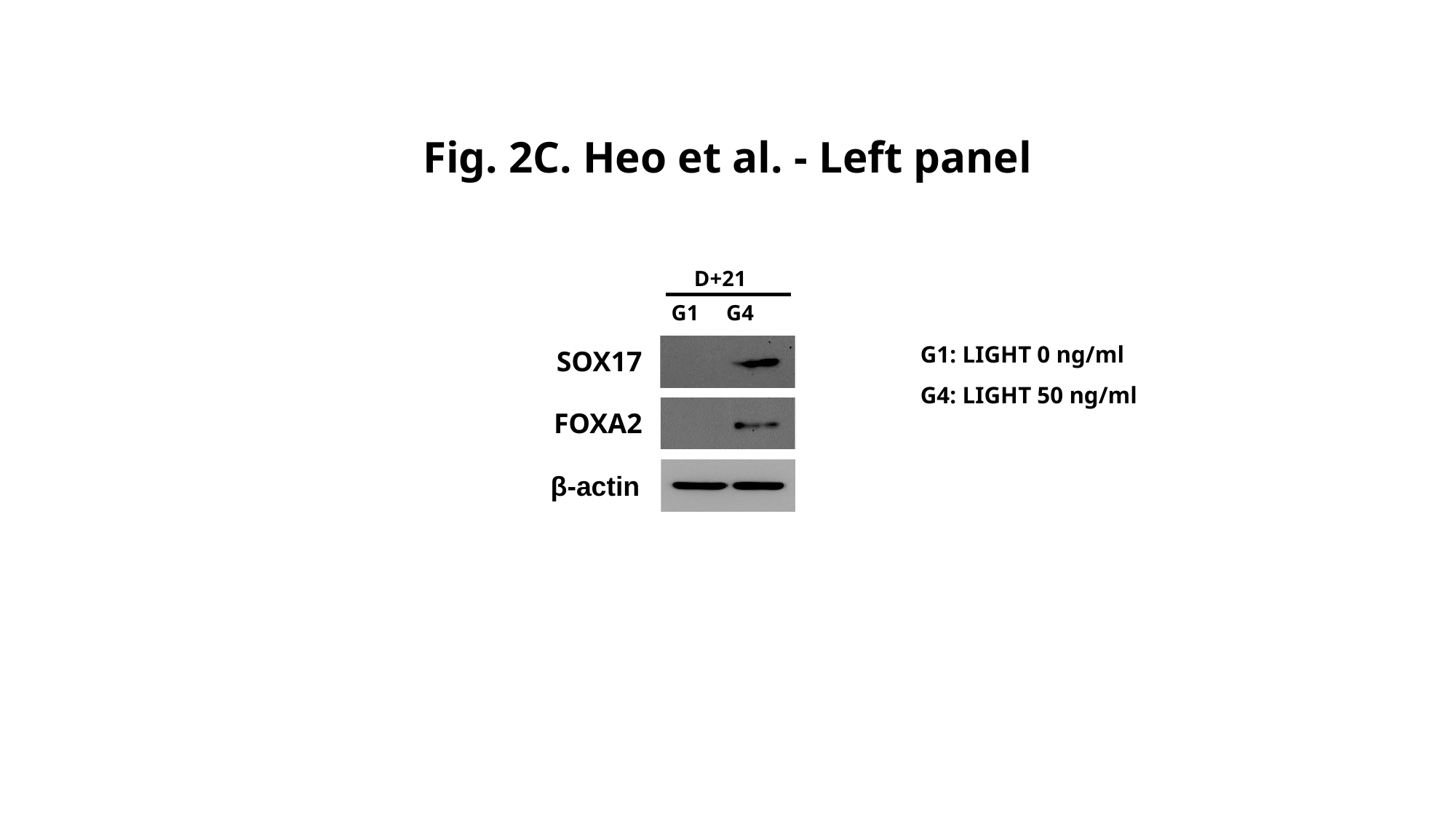

Fig. 2C. Heo et al. - Left panel
D+21
G1 G4
G1: LIGHT 0 ng/ml
G4: LIGHT 50 ng/ml
SOX17
FOXA2
β-actin

## Slide 15
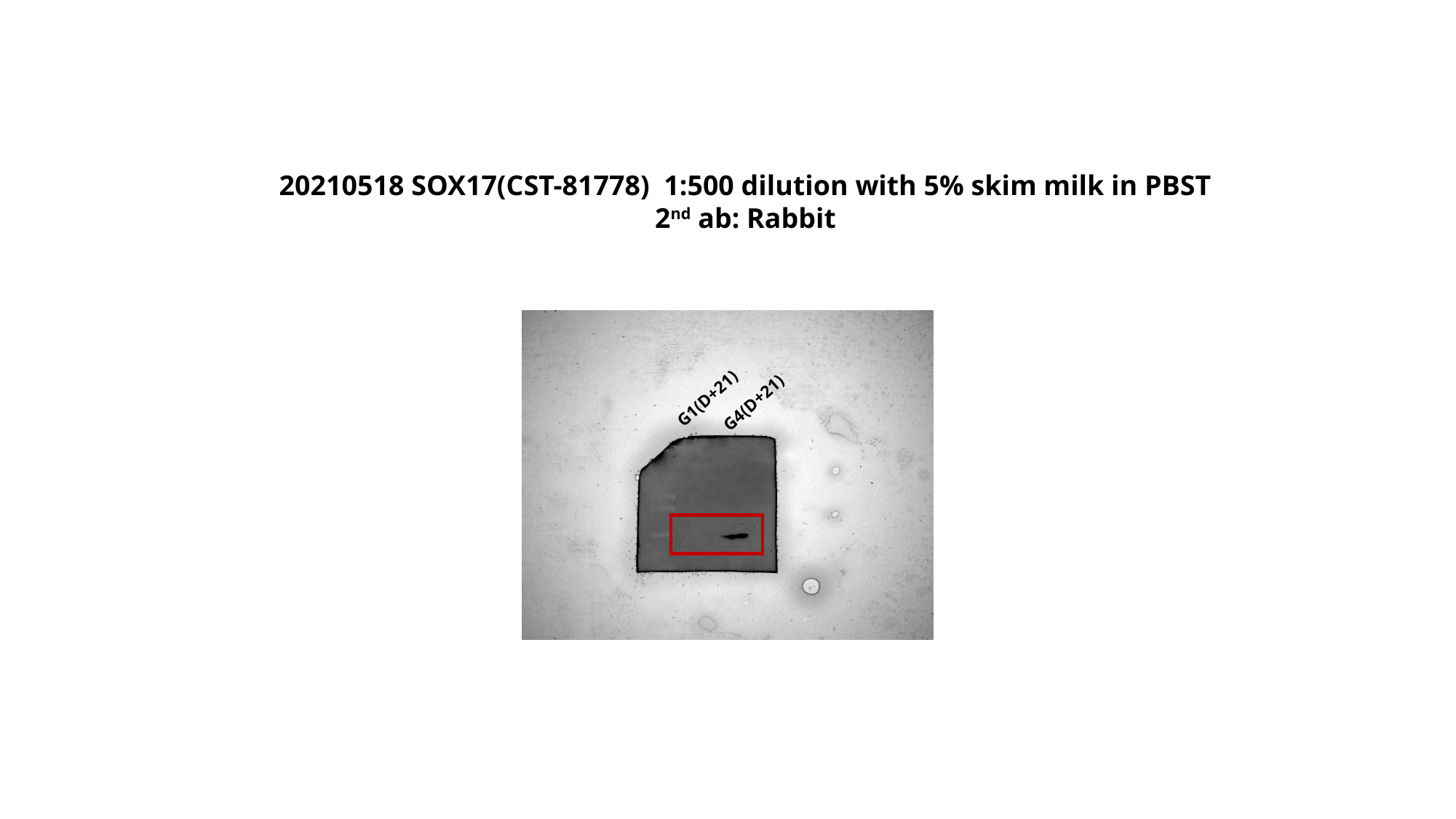

20210518 SOX17(CST-81778) 1:500 dilution with 5% skim milk in PBST 2nd ab: Rabbit
G1(D+21)
G4(D+21)

## Slide 16
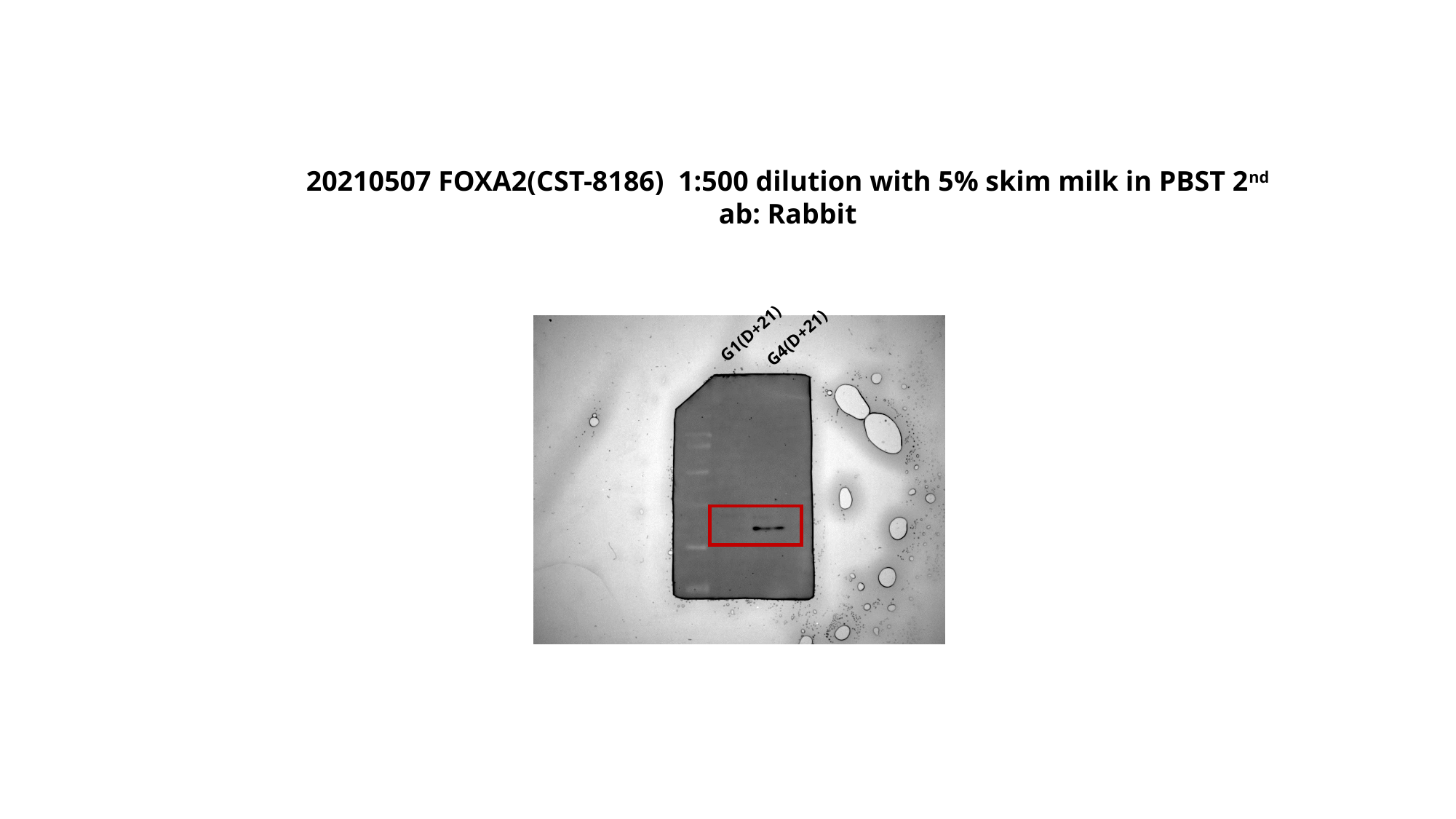

20210507 FOXA2(CST-8186) 1:500 dilution with 5% skim milk in PBST 2nd ab: Rabbit
G1(D+21)
G4(D+21)

## Slide 17
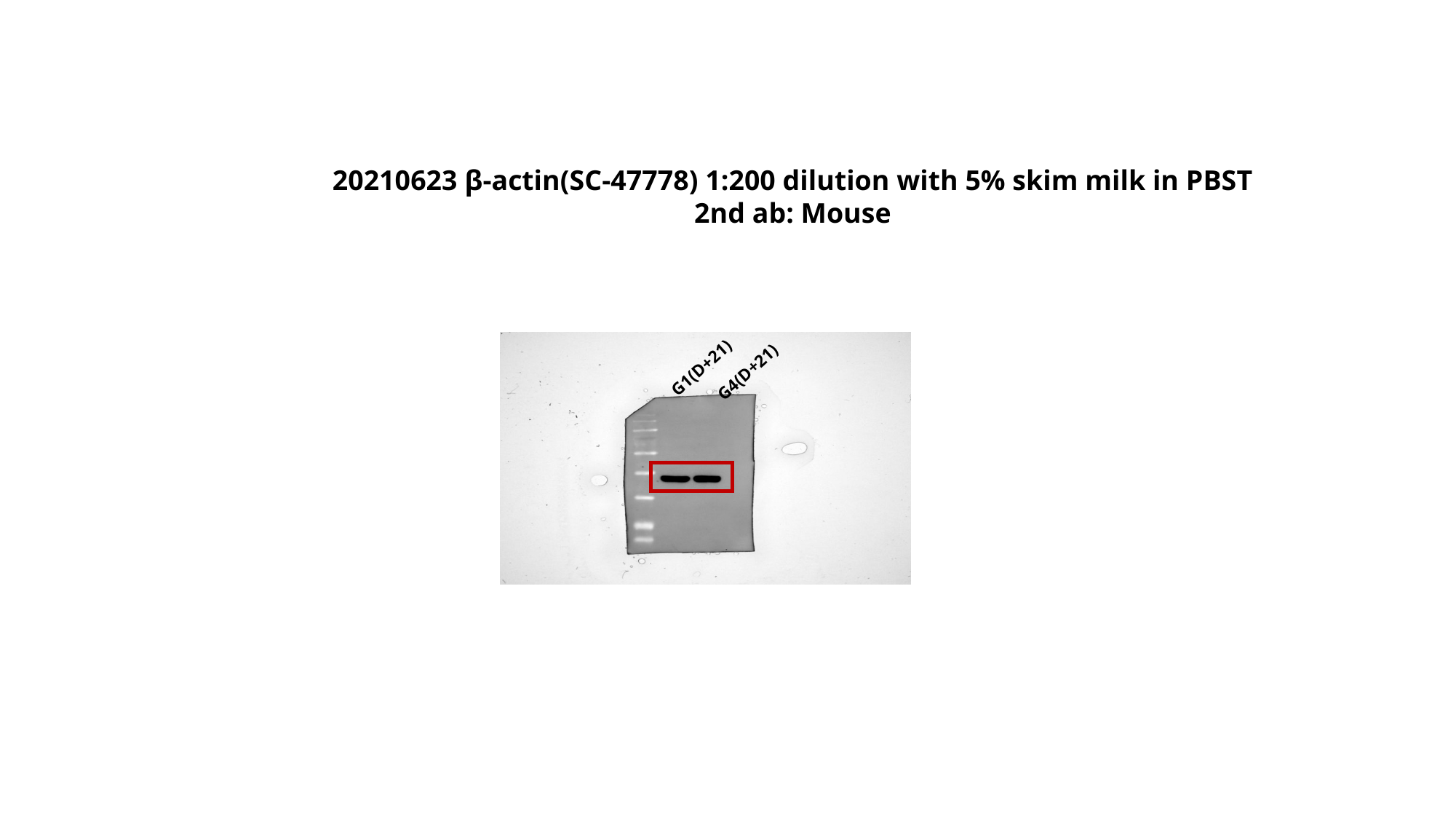

20210623 β-actin(SC-47778) 1:200 dilution with 5% skim milk in PBST 2nd ab: Mouse
G1(D+21)
G4(D+21)

## Slide 18
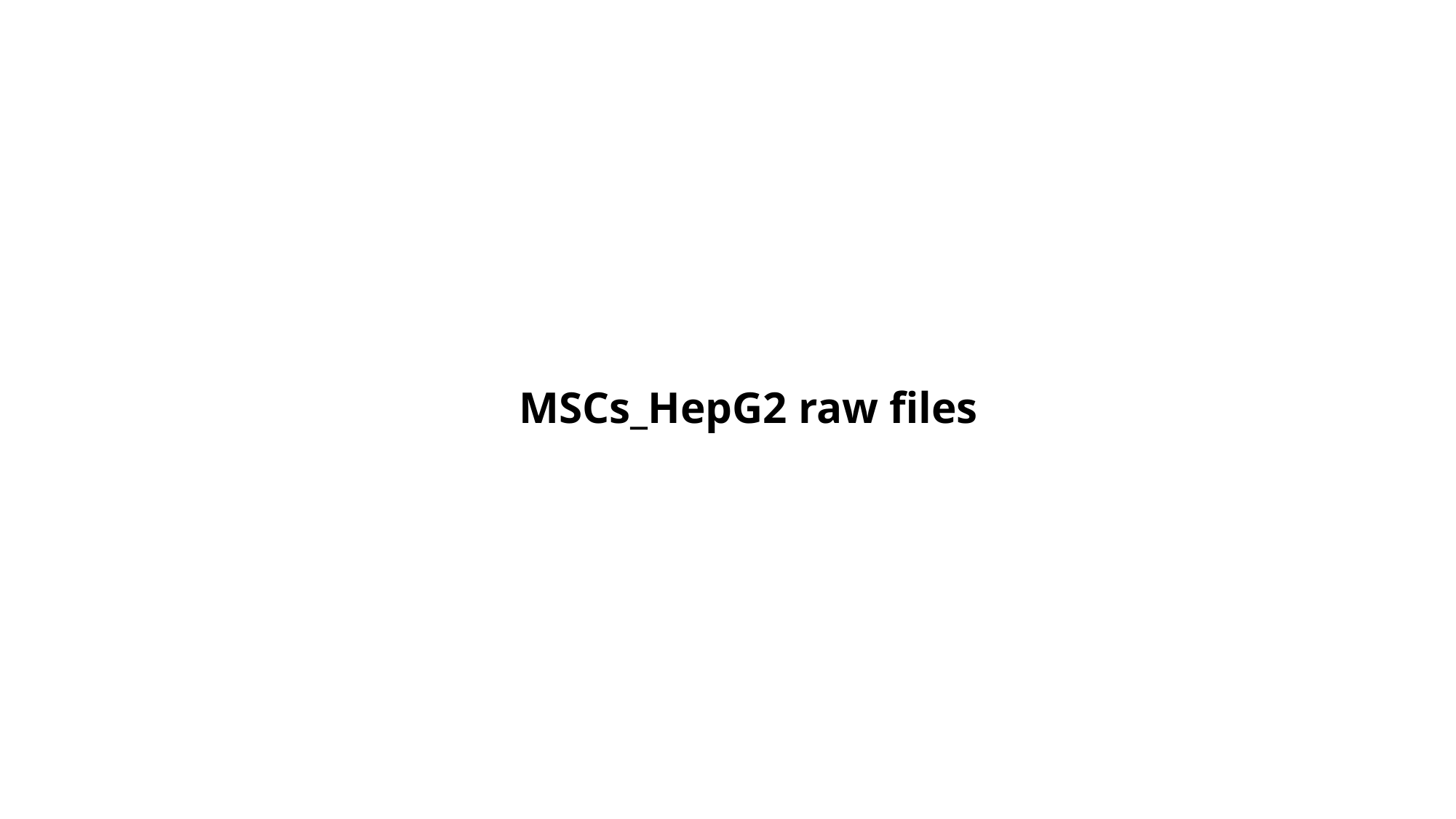

# MSCs_HepG2 raw files

## Slide 19
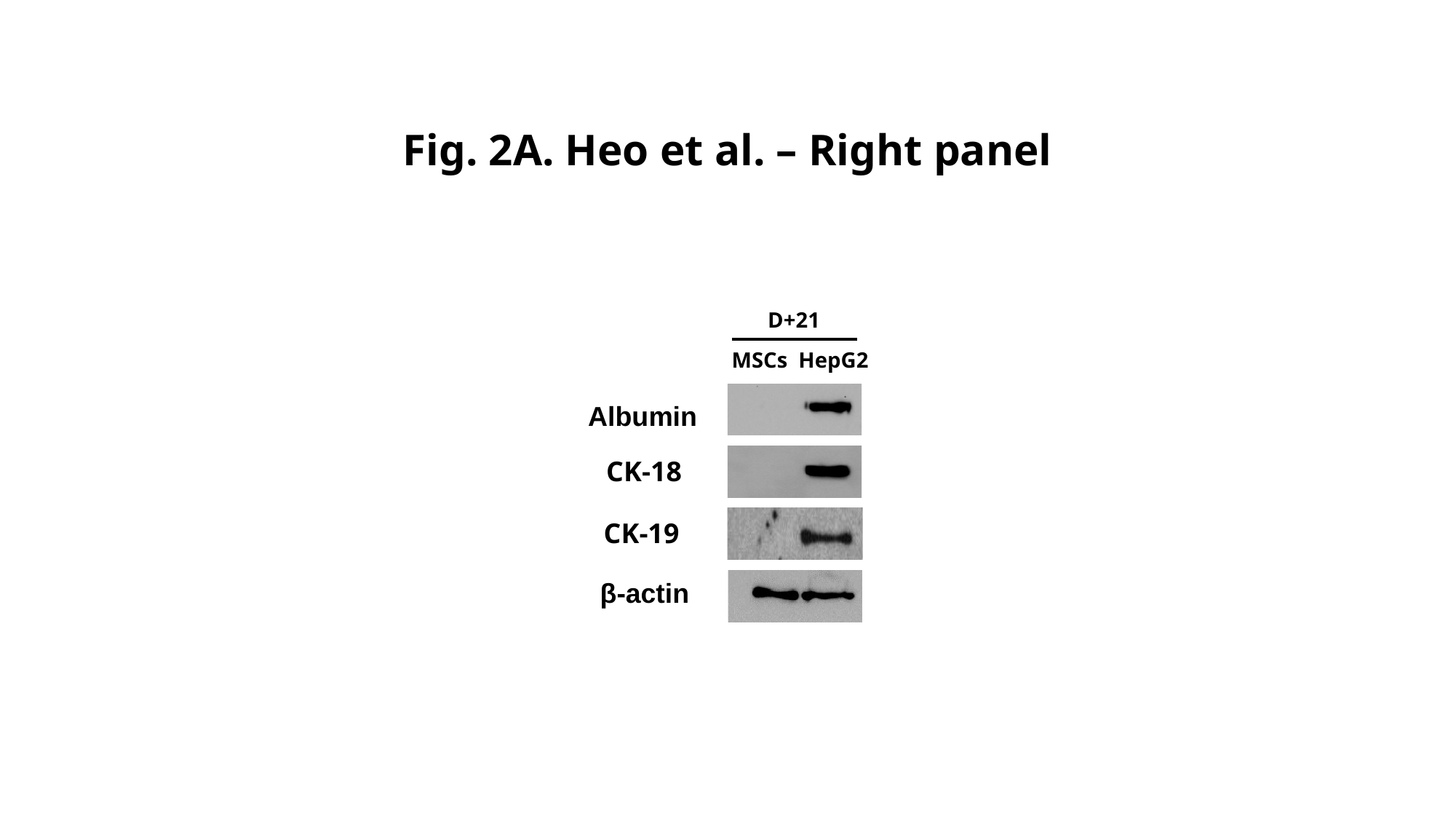

# Fig. 2A. Heo et al. – Right panel
D+21
MSCs HepG2
Albumin
CK-18
CK-19
β-actin

## Slide 20
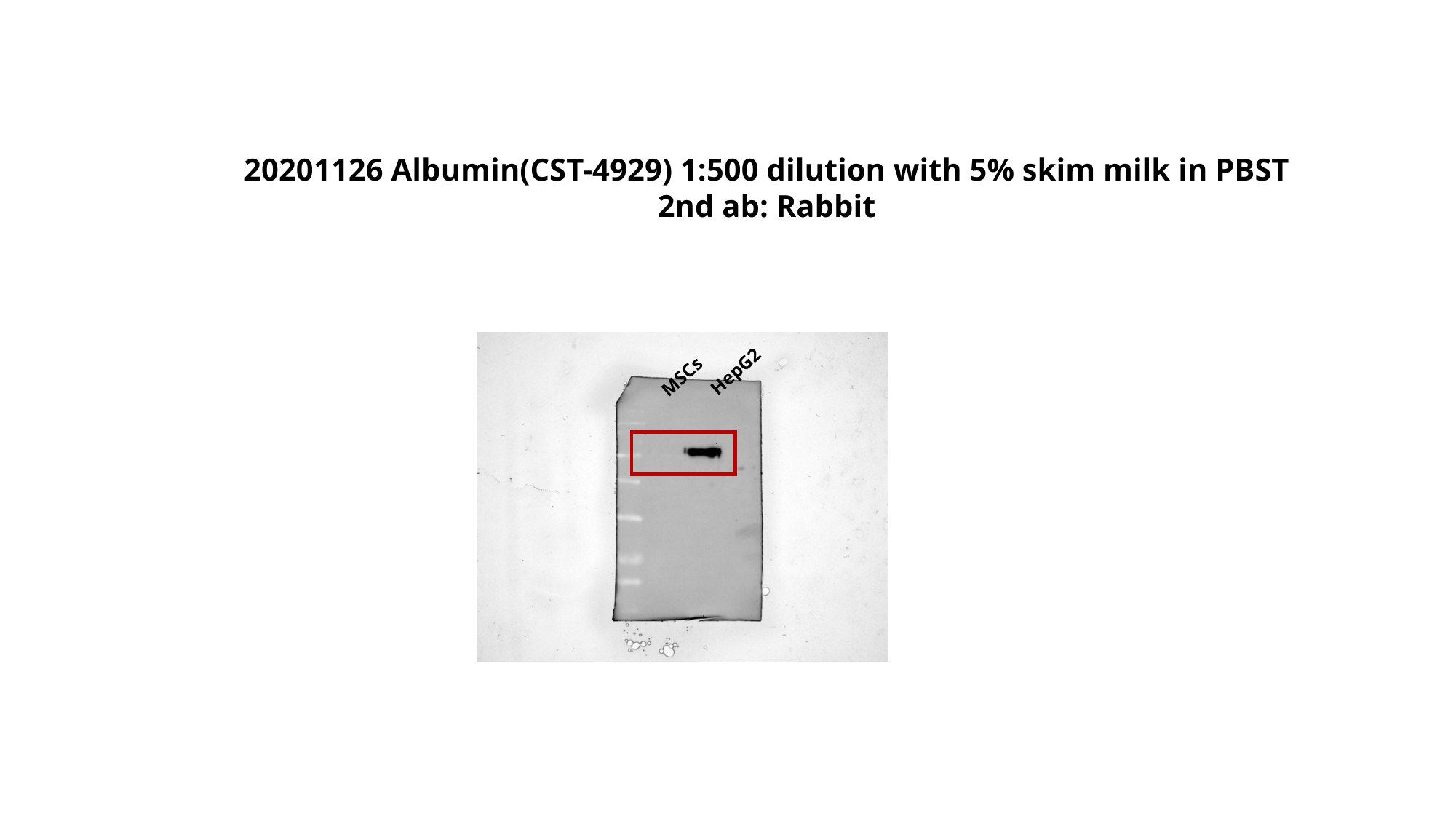

20201126 Albumin(CST-4929) 1:500 dilution with 5% skim milk in PBST 2nd ab: Rabbit
HepG2
MSCs

## Slide 21
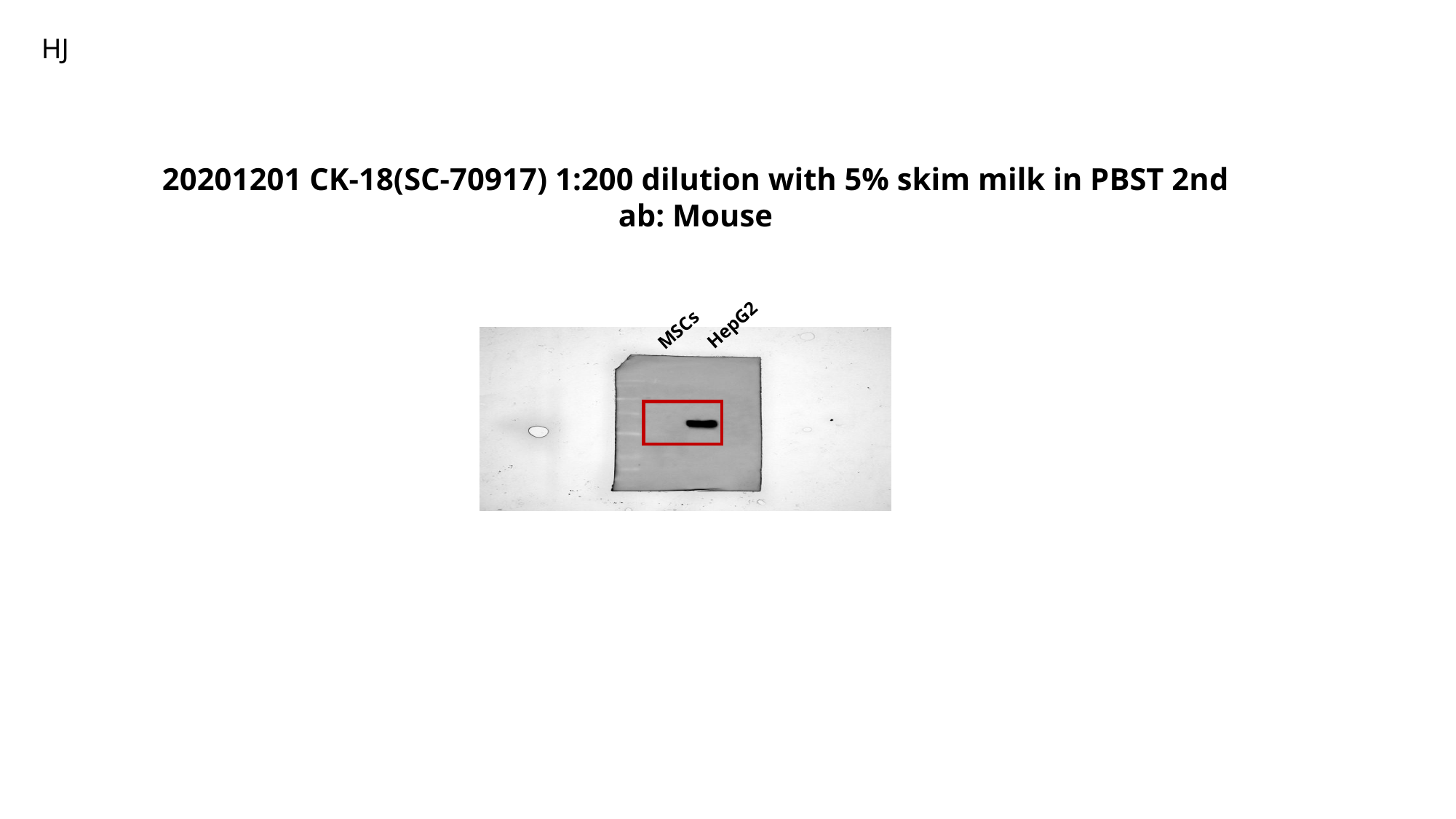

HJ
20201201 CK-18(SC-70917) 1:200 dilution with 5% skim milk in PBST 2nd ab: Mouse
HepG2
MSCs

## Slide 22
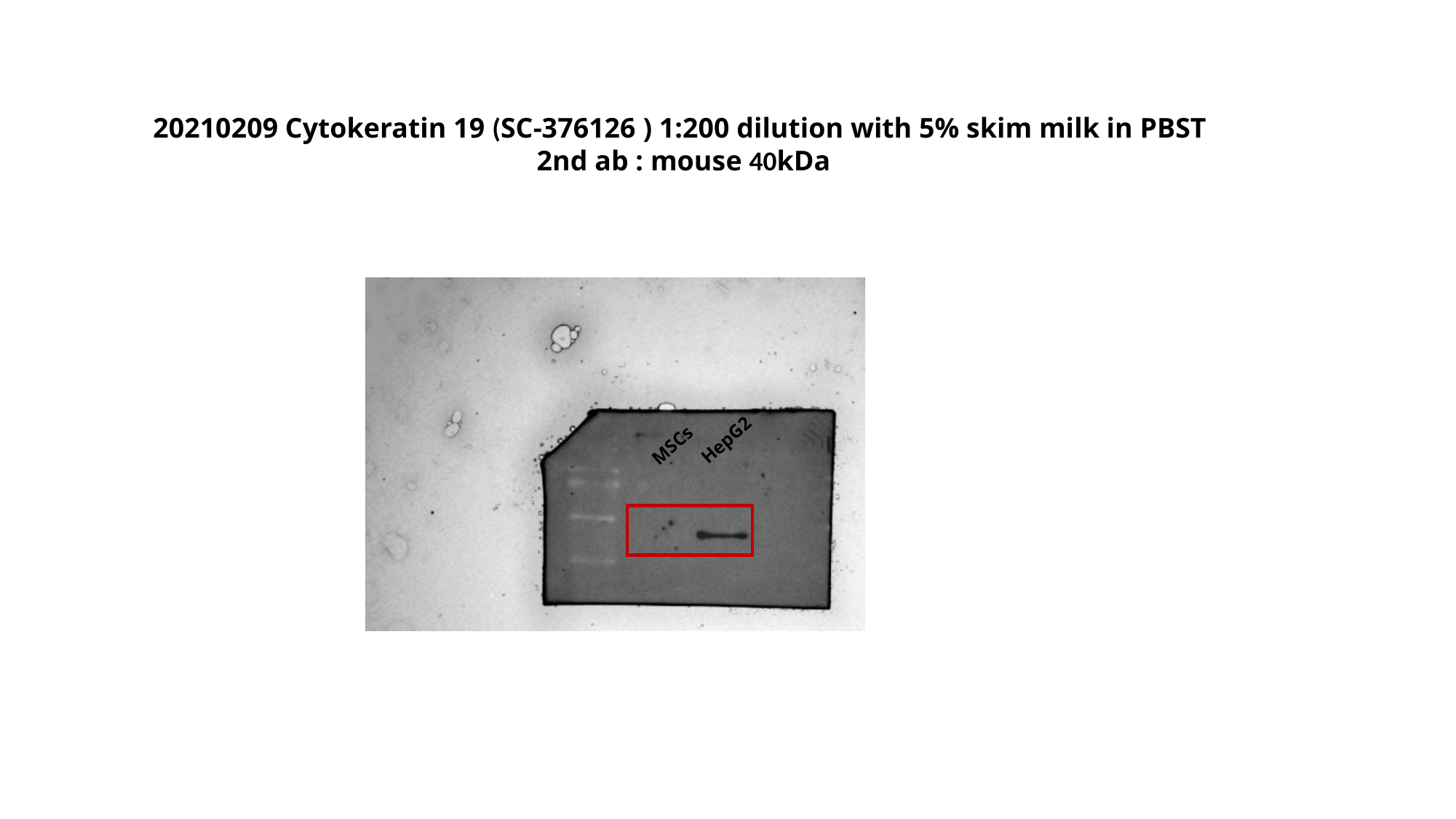

20210209 Cytokeratin 19 (SC-376126 ) 1:200 dilution with 5% skim milk in PBST
2nd ab : mouse 40kDa
HepG2
MSCs

## Slide 23
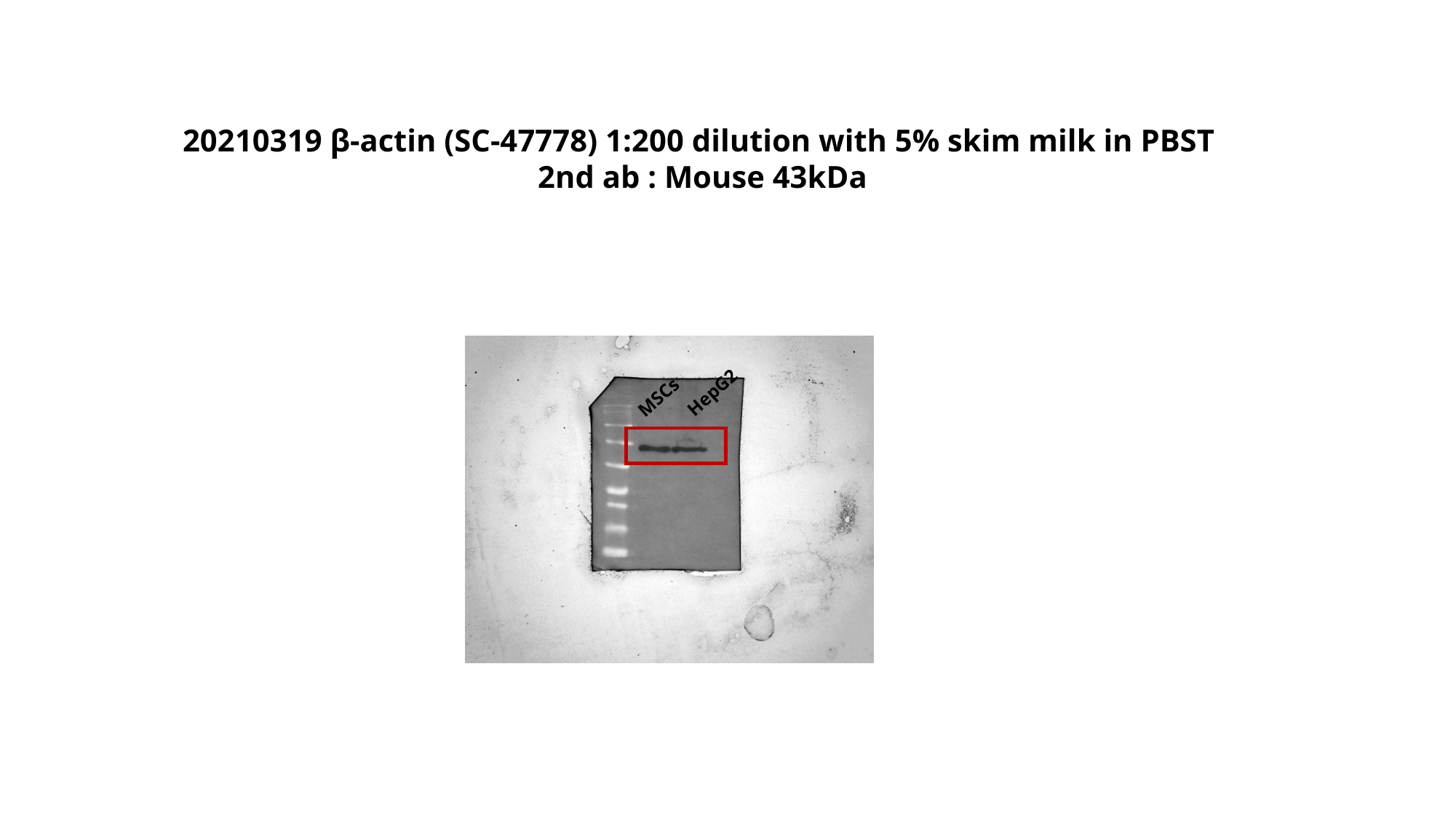

20210319 β-actin (SC-47778) 1:200 dilution with 5% skim milk in PBST
2nd ab : Mouse 43kDa
HepG2
MSCs

## Slide 24
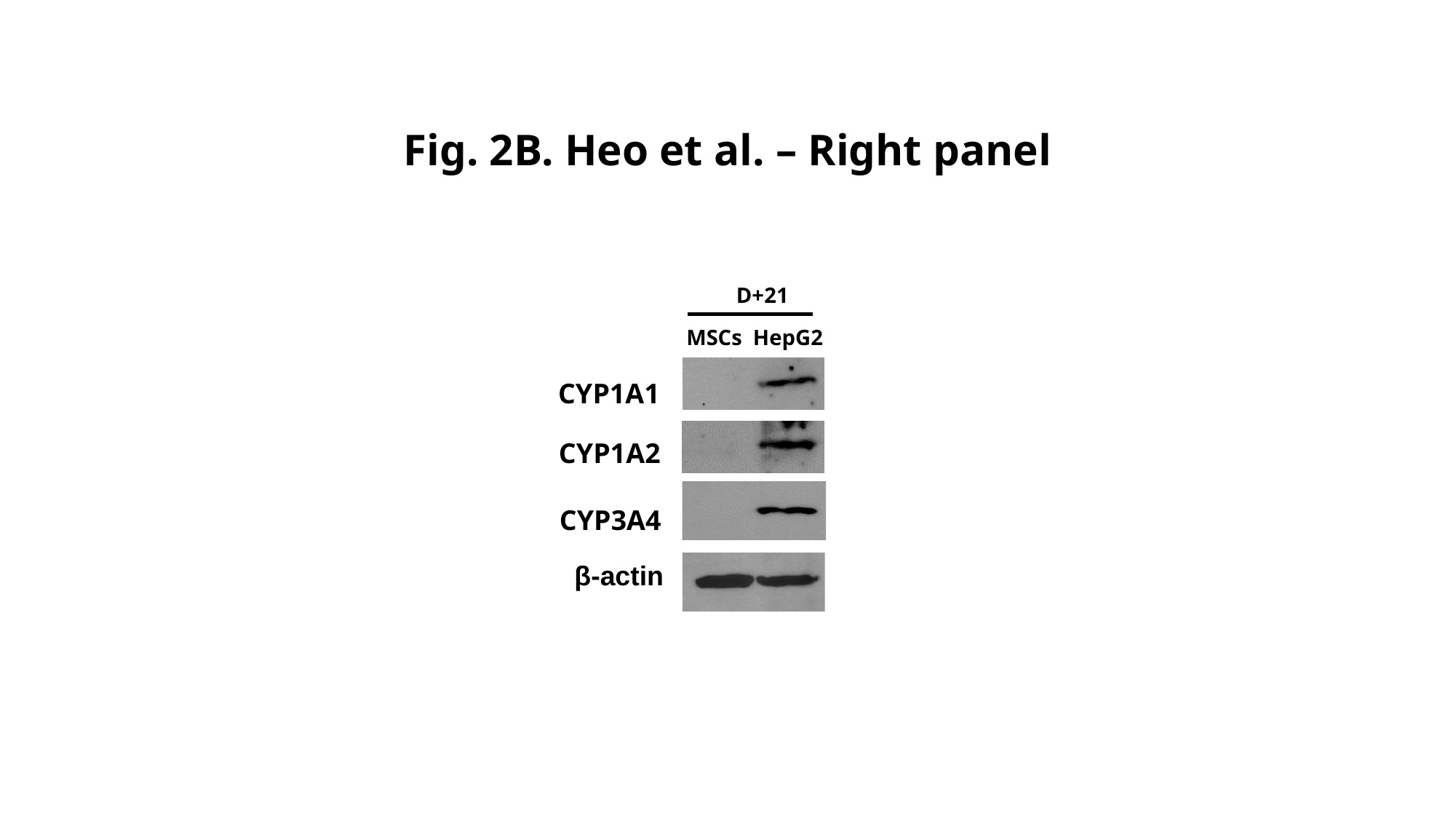

# Fig. 2B. Heo et al. – Right panel
D+21
MSCs HepG2
CYP1A1
CYP1A2
CYP3A4
β-actin

## Slide 25
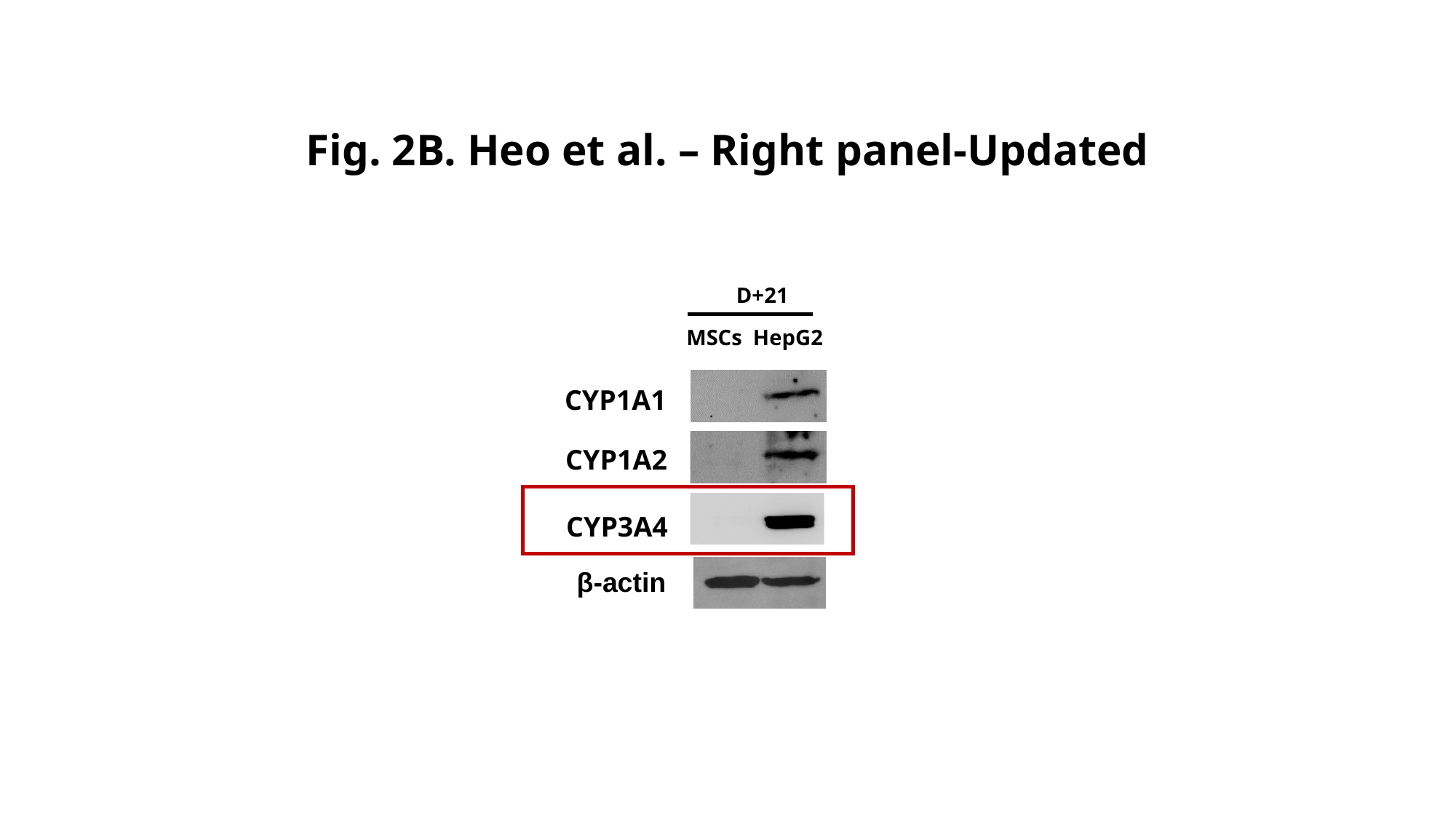

# Fig. 2B. Heo et al. – Right panel-Updated
D+21
MSCs HepG2
CYP1A1
CYP1A2
CYP3A4
β-actin

## Slide 26
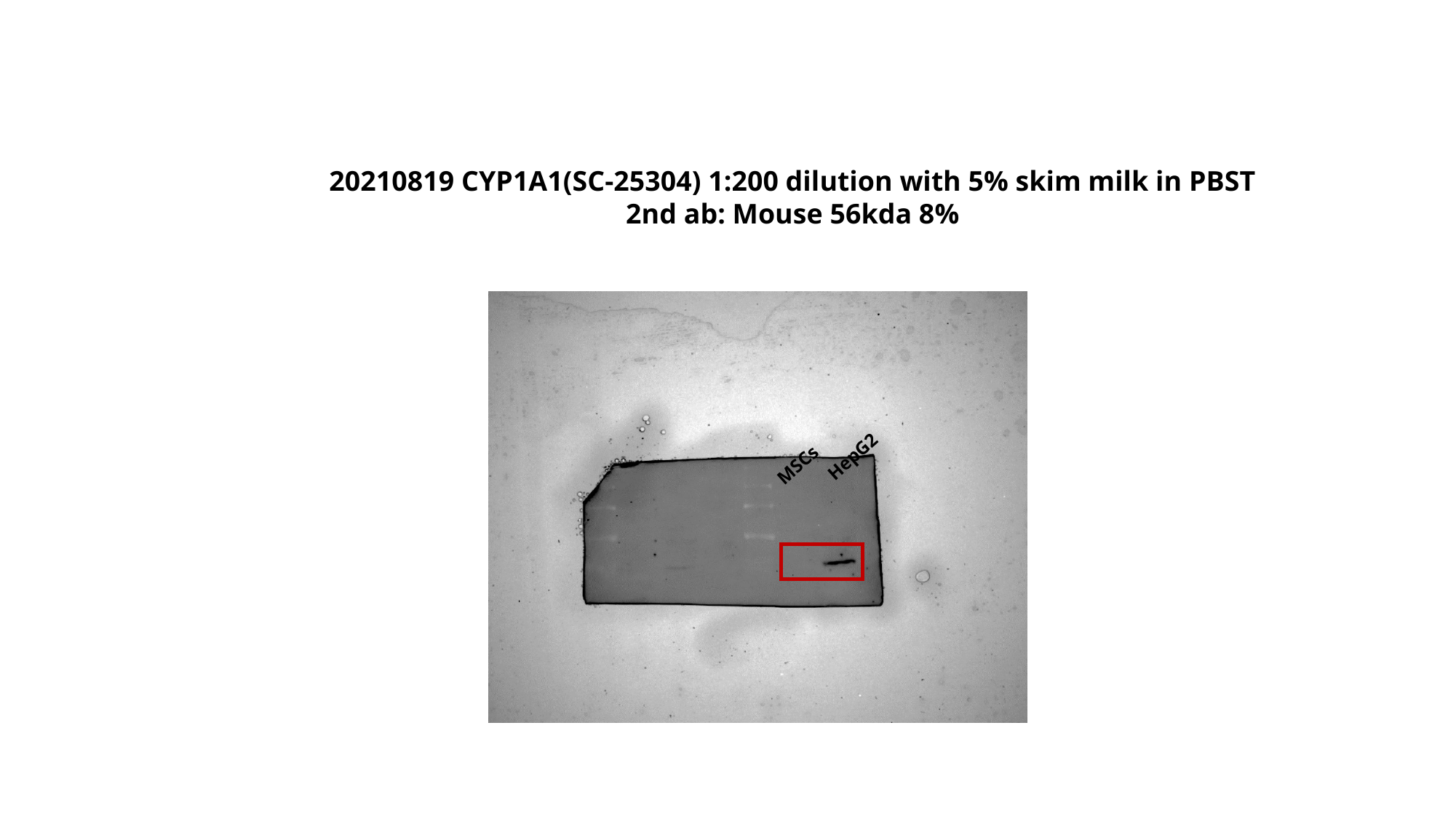

20210819 CYP1A1(SC-25304) 1:200 dilution with 5% skim milk in PBST 2nd ab: Mouse 56kda 8%
HepG2
MSCs

## Slide 27
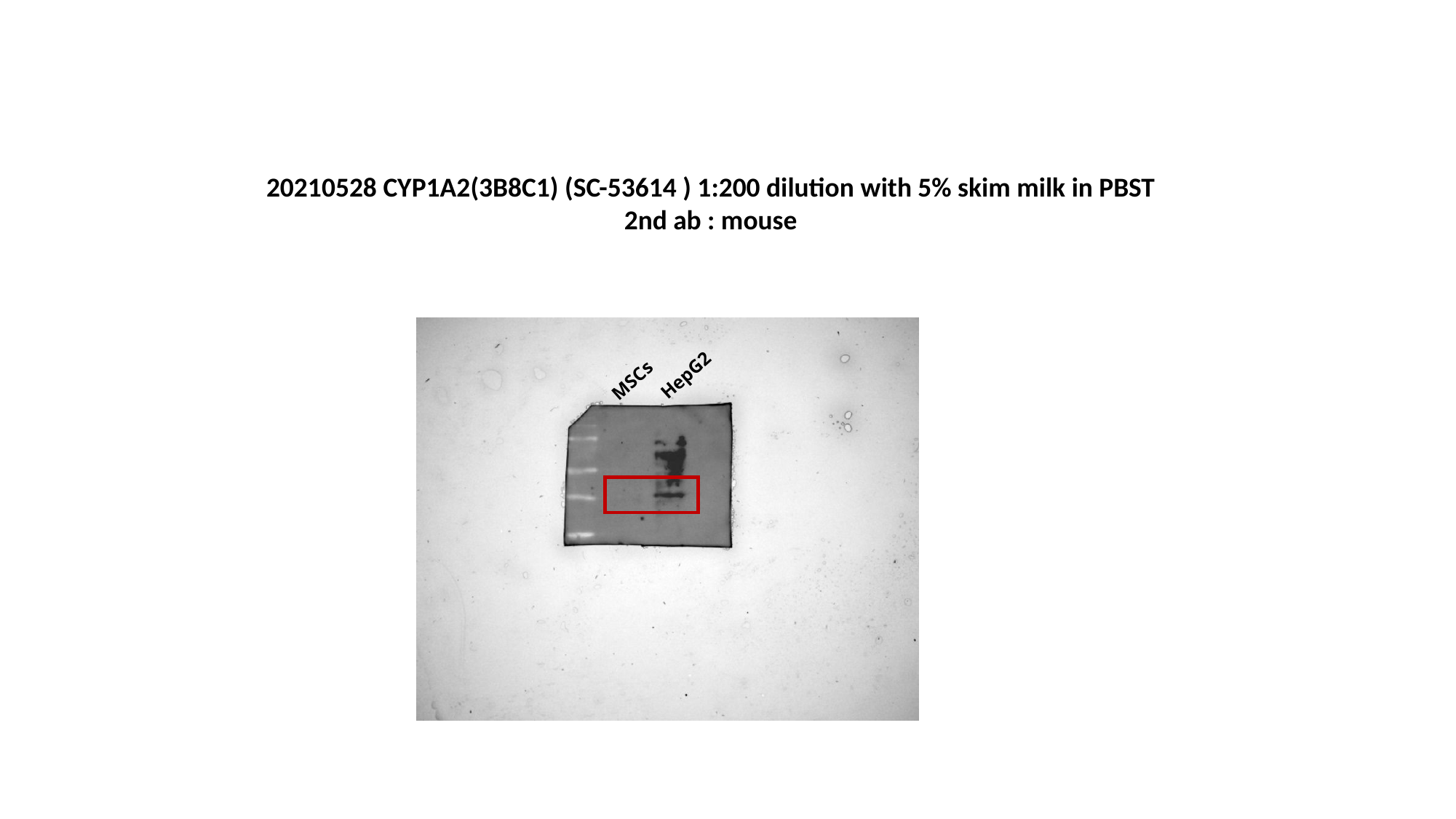

20210528 CYP1A2(3B8C1) (SC-53614 ) 1:200 dilution with 5% skim milk in PBST
2nd ab : mouse
HepG2
MSCs

## Slide 28
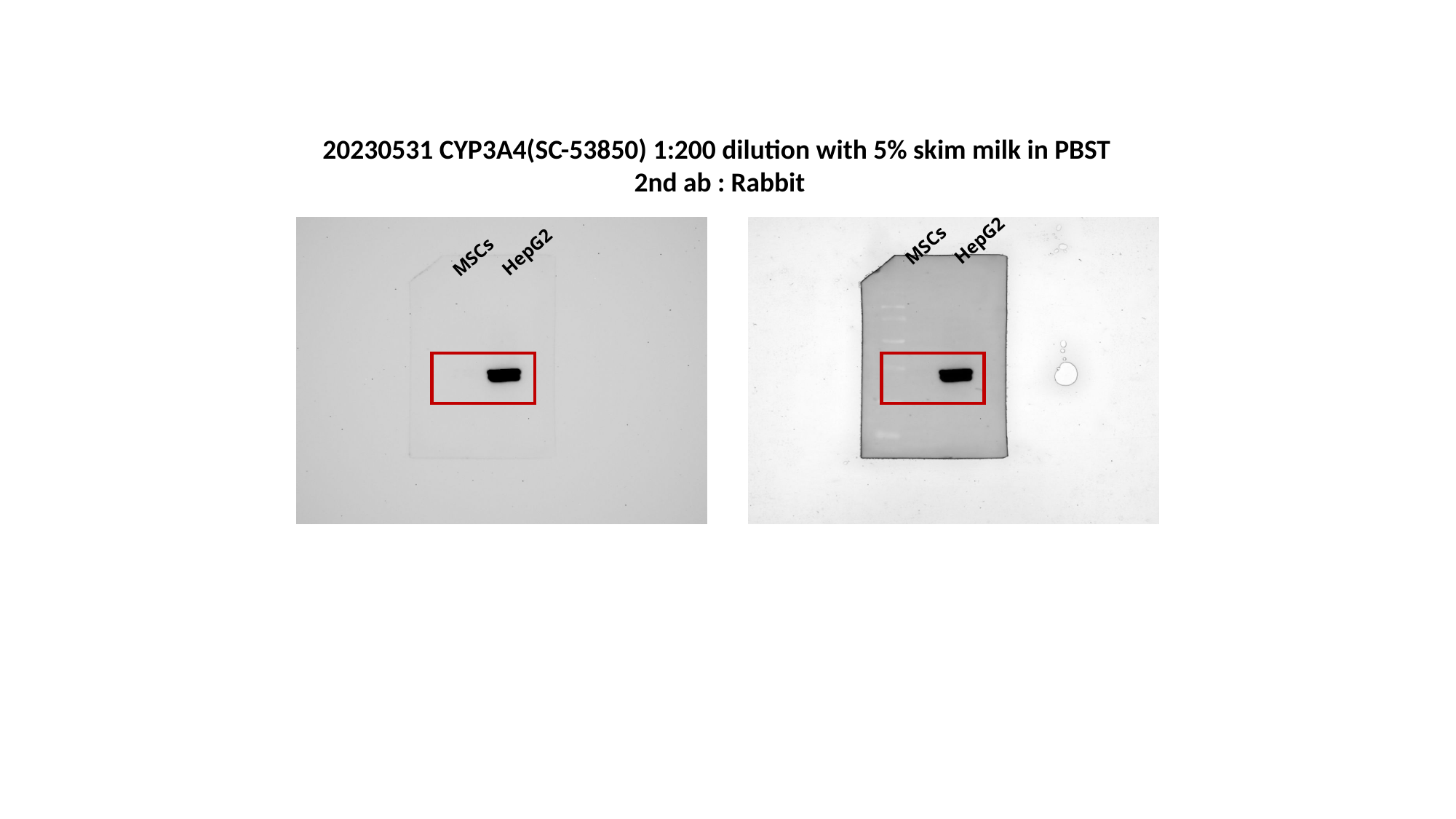

20230531 CYP3A4(SC-53850) 1:200 dilution with 5% skim milk in PBST
2nd ab : Rabbit
HepG2
MSCs
HepG2
MSCs

## Slide 29
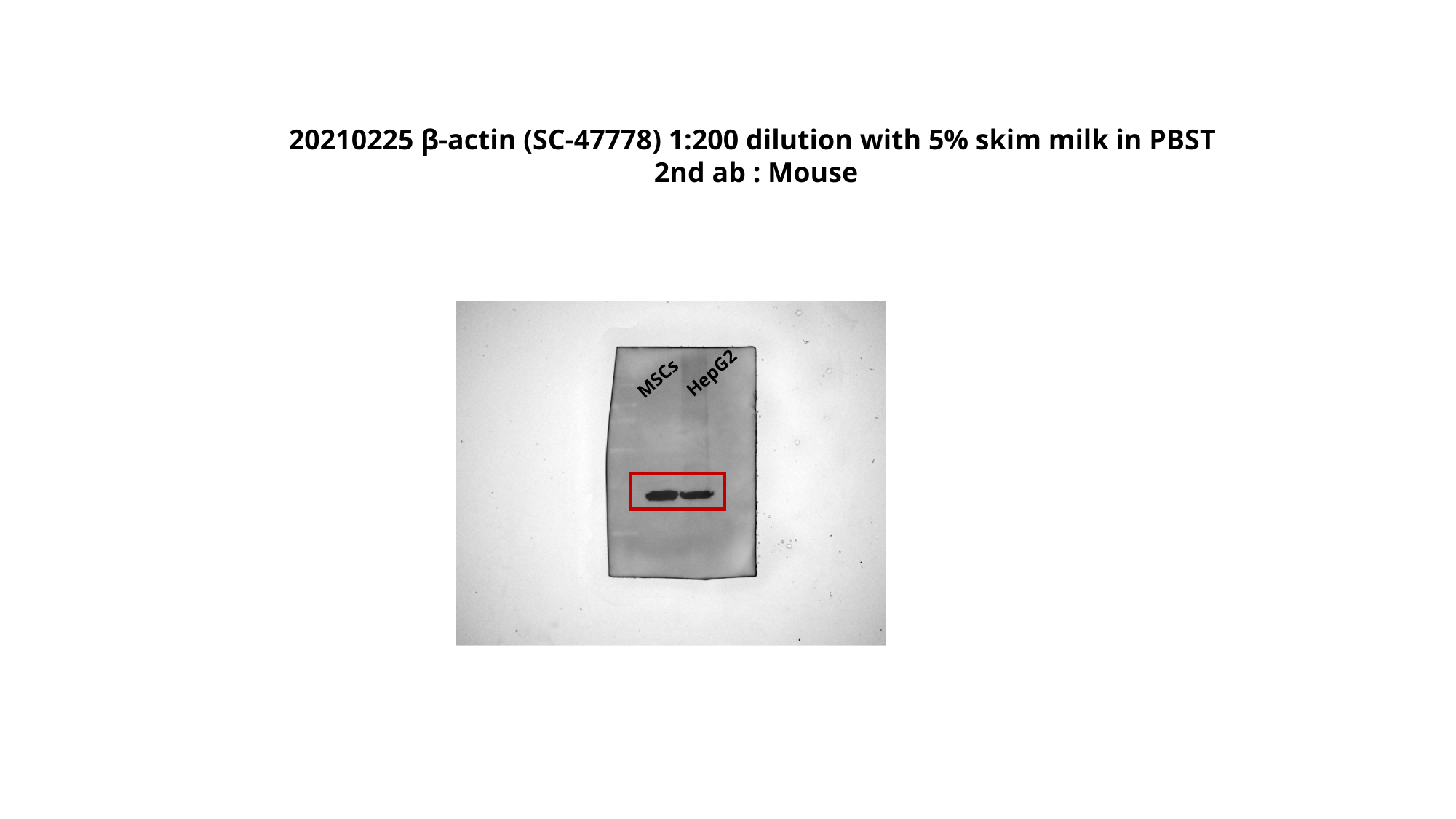

20210225 β-actin (SC-47778) 1:200 dilution with 5% skim milk in PBST
2nd ab : Mouse
HepG2
MSCs

## Slide 30
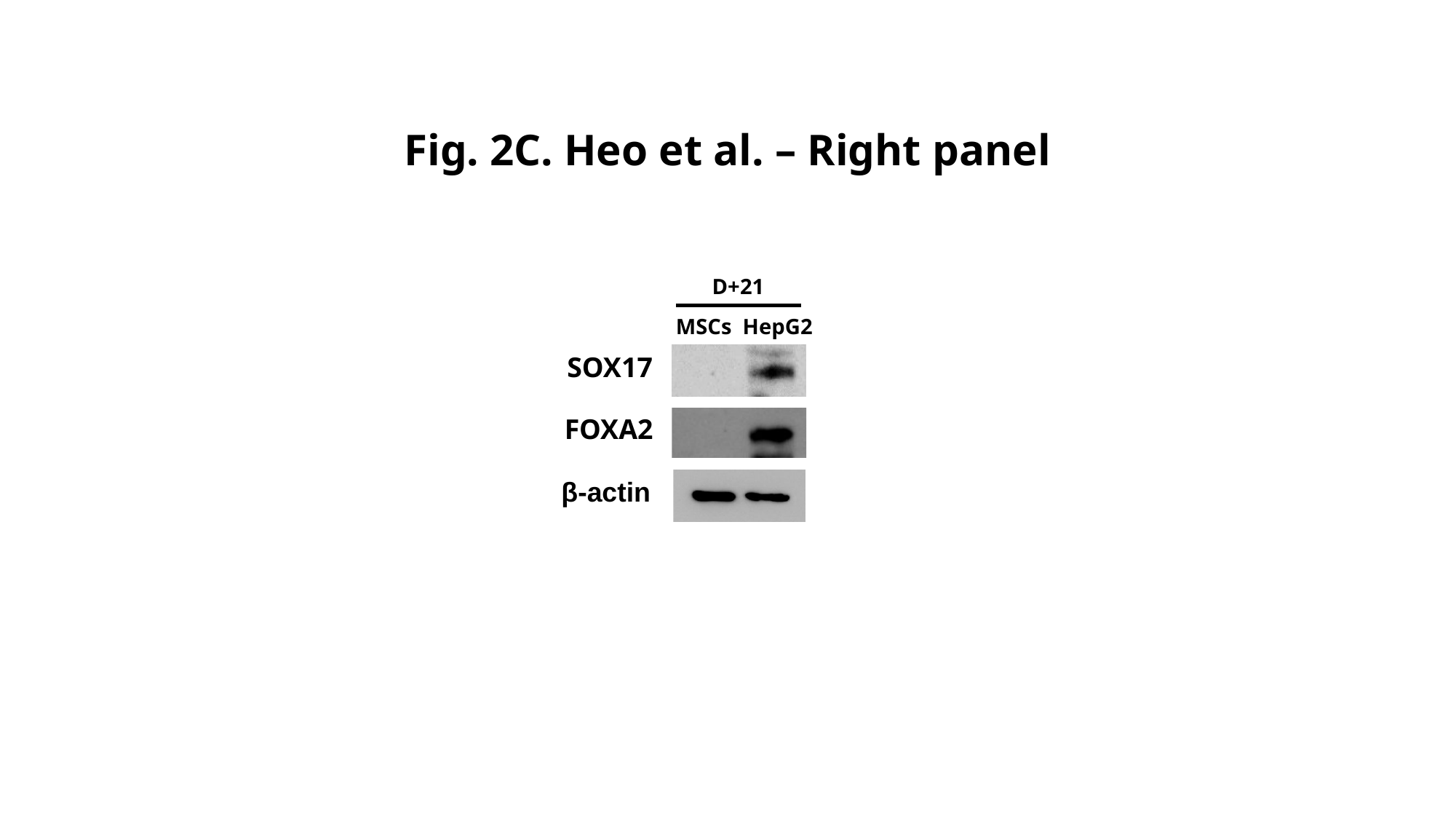

# Fig. 2C. Heo et al. – Right panel
D+21
MSCs HepG2
SOX17
FOXA2
β-actin

## Slide 31
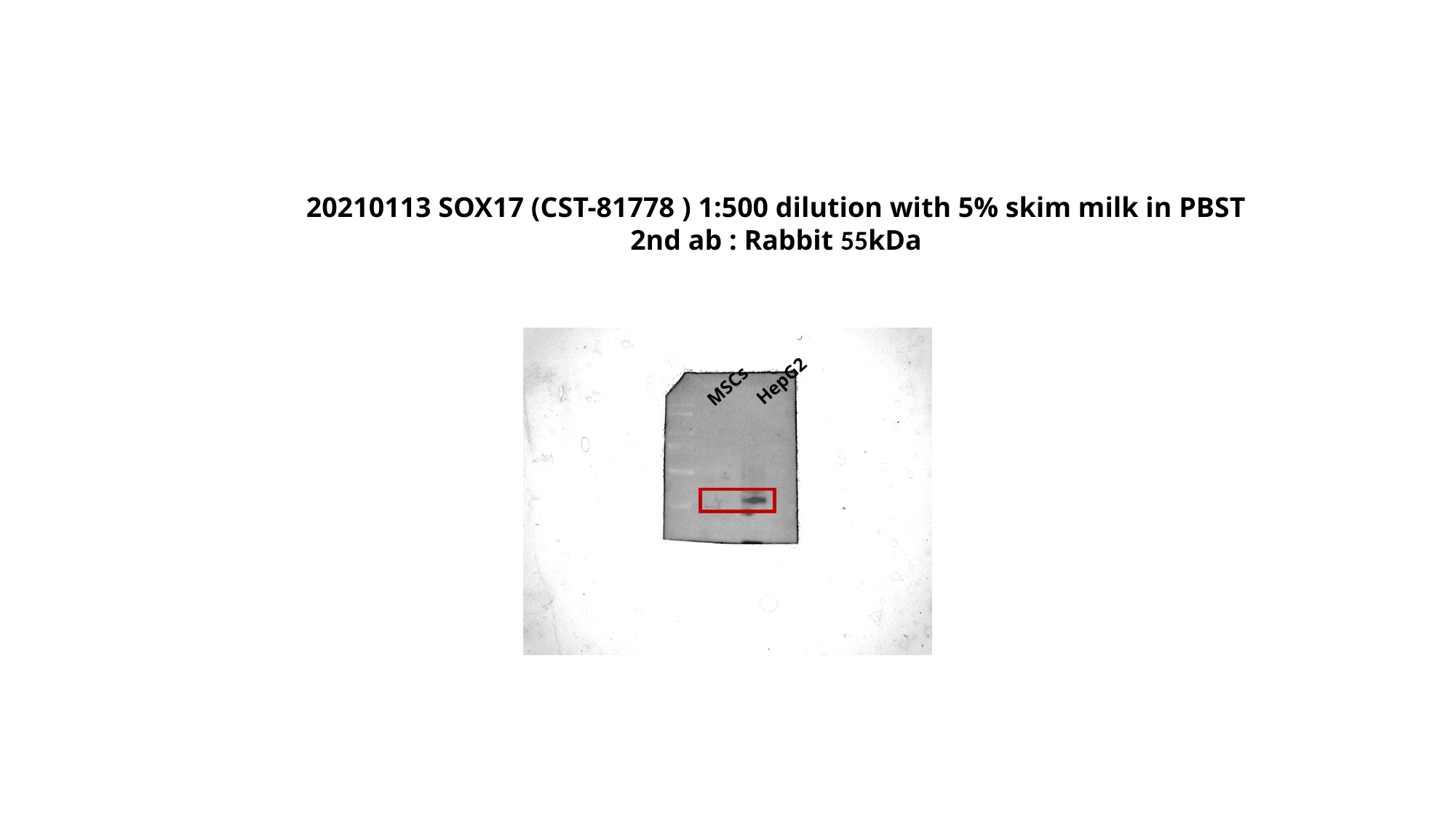

20210113 SOX17 (CST-81778 ) 1:500 dilution with 5% skim milk in PBST 2nd ab : Rabbit 55kDa
HepG2
MSCs

## Slide 32
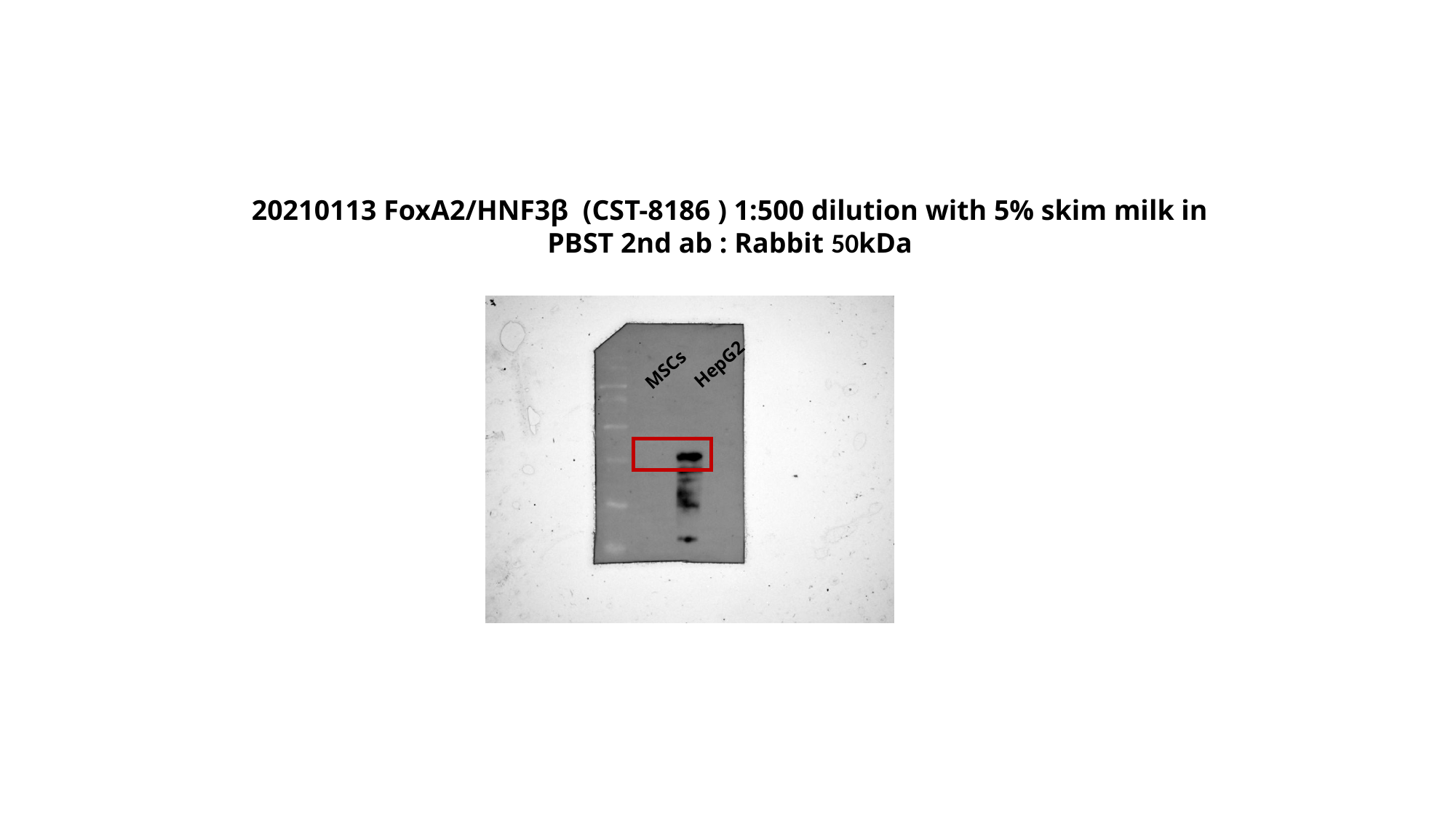

20210113 FoxA2/HNF3β (CST-8186 ) 1:500 dilution with 5% skim milk in PBST 2nd ab : Rabbit 50kDa
HepG2
MSCs

## Slide 33
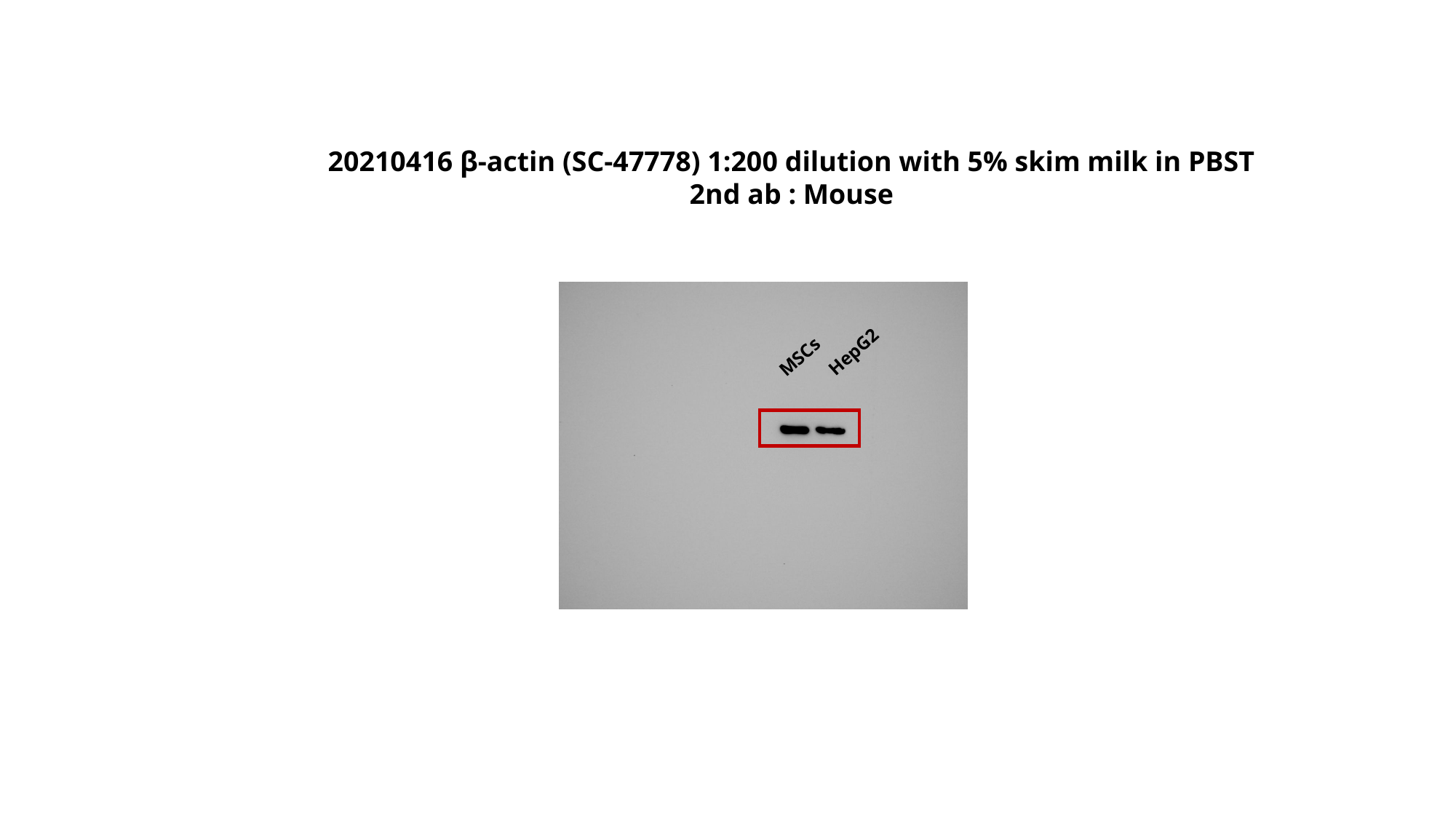

20210416 β-actin (SC-47778) 1:200 dilution with 5% skim milk in PBST 2nd ab : Mouse
HepG2
MSCs
