# Supplementary material for: LIGHT (TNFSF14) promotes the differentiation of human bone marrow-derived mesenchymal stem cells into functional hepatocyte-like cells
Source: PLoS One. 2023 Aug 8;18(8):e0289798. doi: 10.1371/journal.pone.0289798 (PMC10411951; doi:10.1371/journal.pone.0289798)
Supplement: S2 Raw images — (PPTX) [file pone.0289798.s003.pptx]

## Slide 1
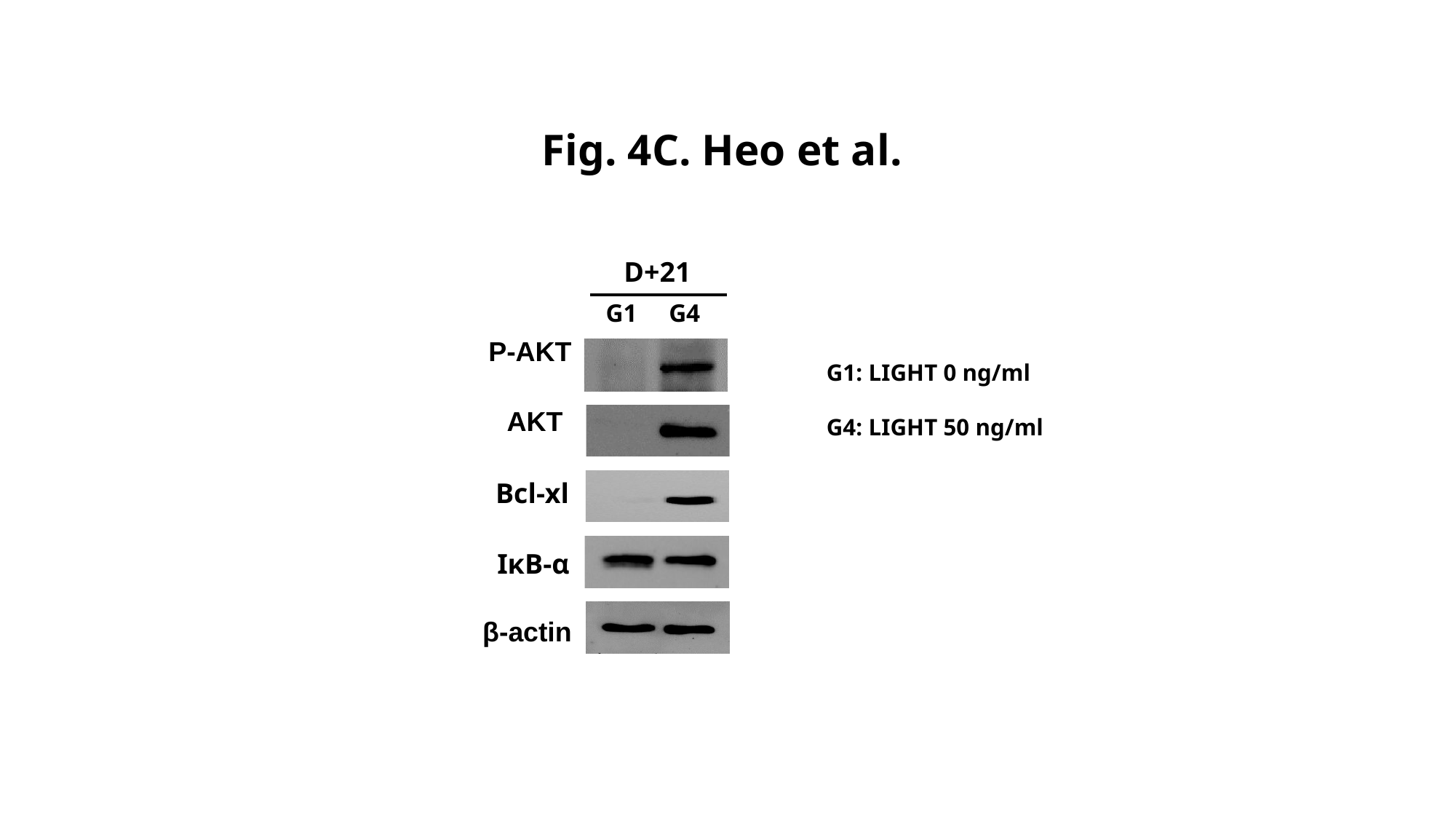

# Fig. 4C. Heo et al.
D+21
G1 G4
G1: LIGHT 0 ng/ml
G4: LIGHT 50 ng/ml
P-AKT
AKT
Bcl-xl
IκB-α
β-actin

## Slide 2
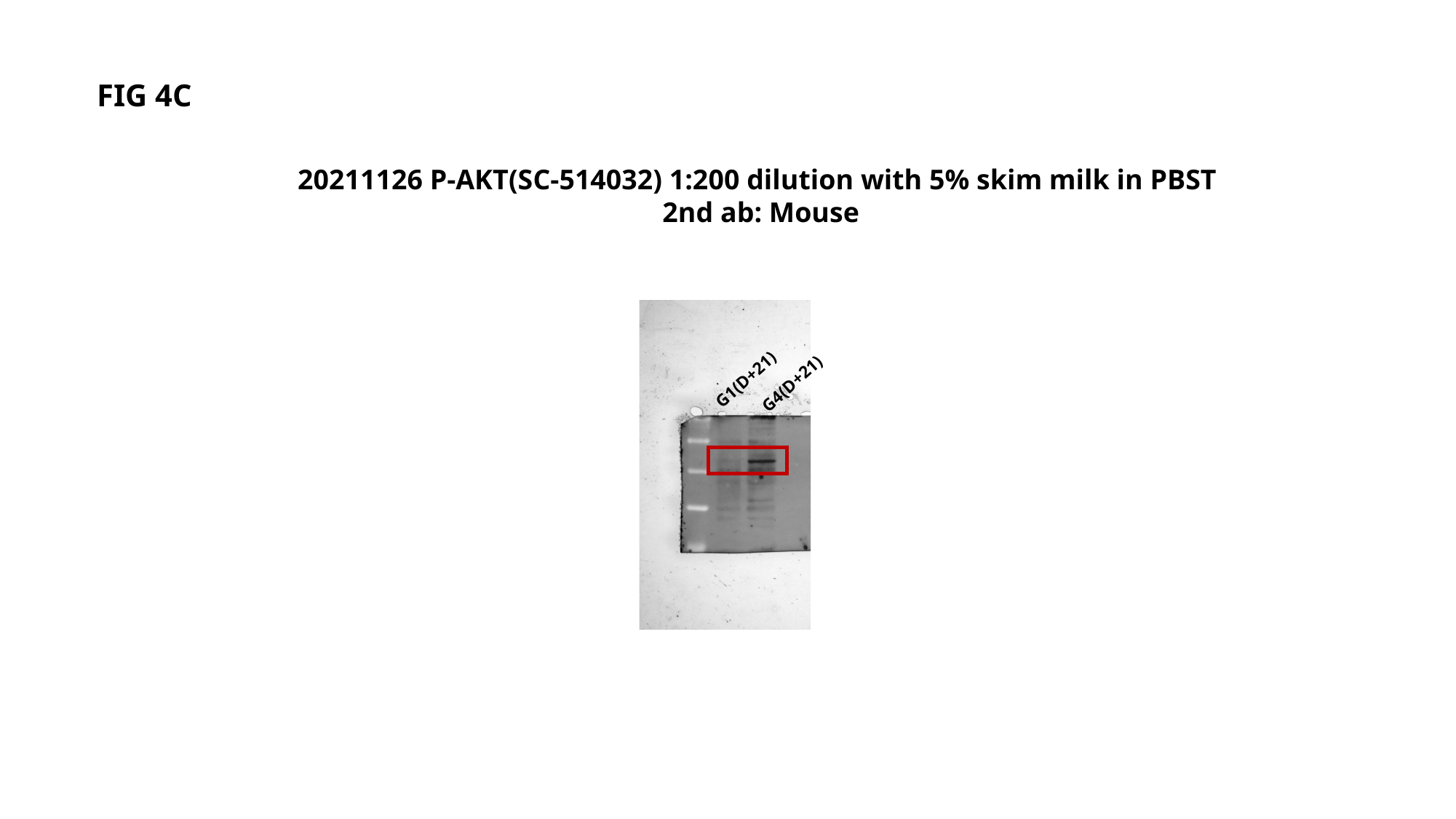

FIG 4C
20211126 P-AKT(SC-514032) 1:200 dilution with 5% skim milk in PBST 2nd ab: Mouse
G1(D+21)
G4(D+21)

## Slide 3
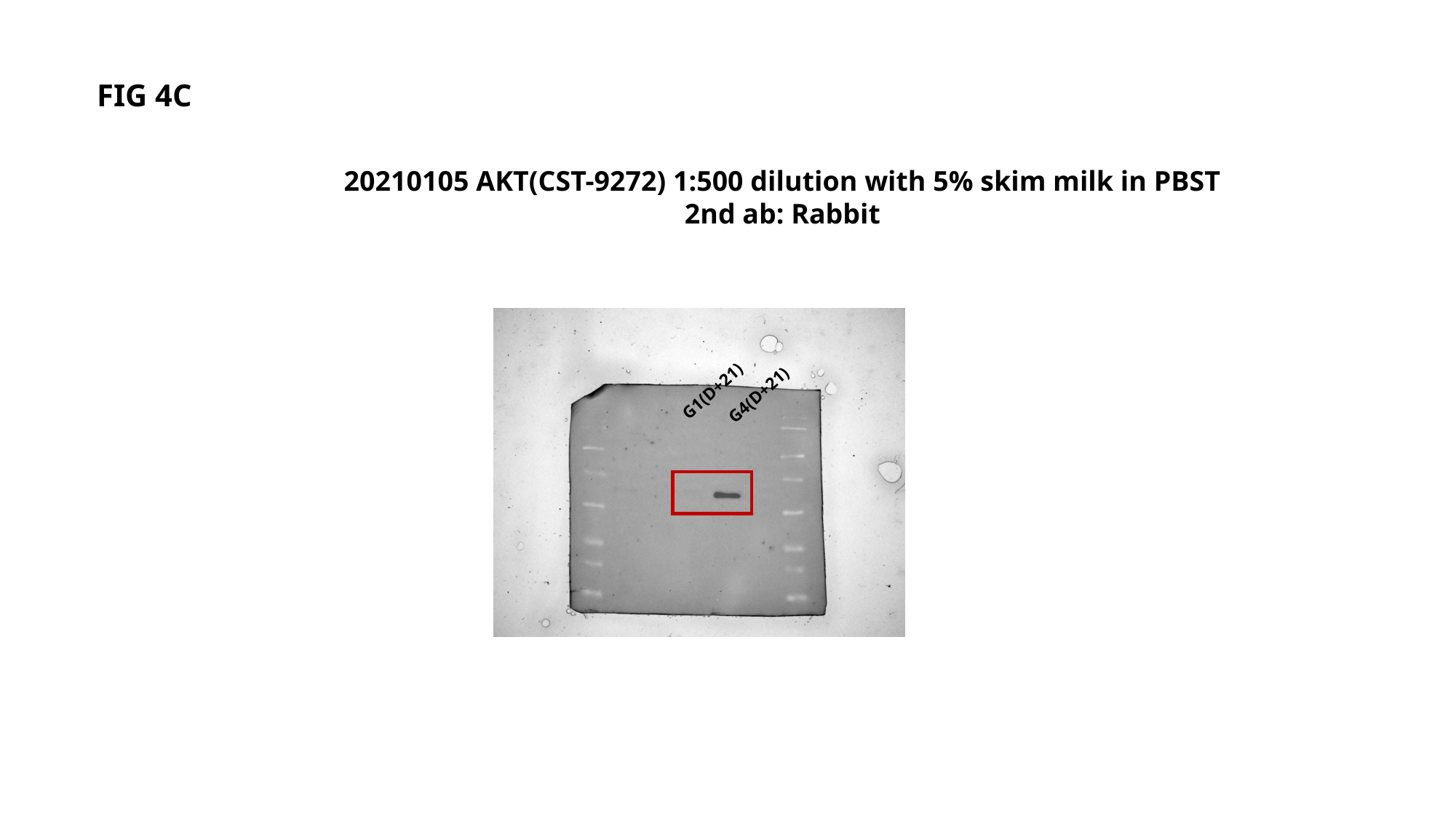

FIG 4C
20210105 AKT(CST-9272) 1:500 dilution with 5% skim milk in PBST 2nd ab: Rabbit
G1(D+21)
G4(D+21)

## Slide 4
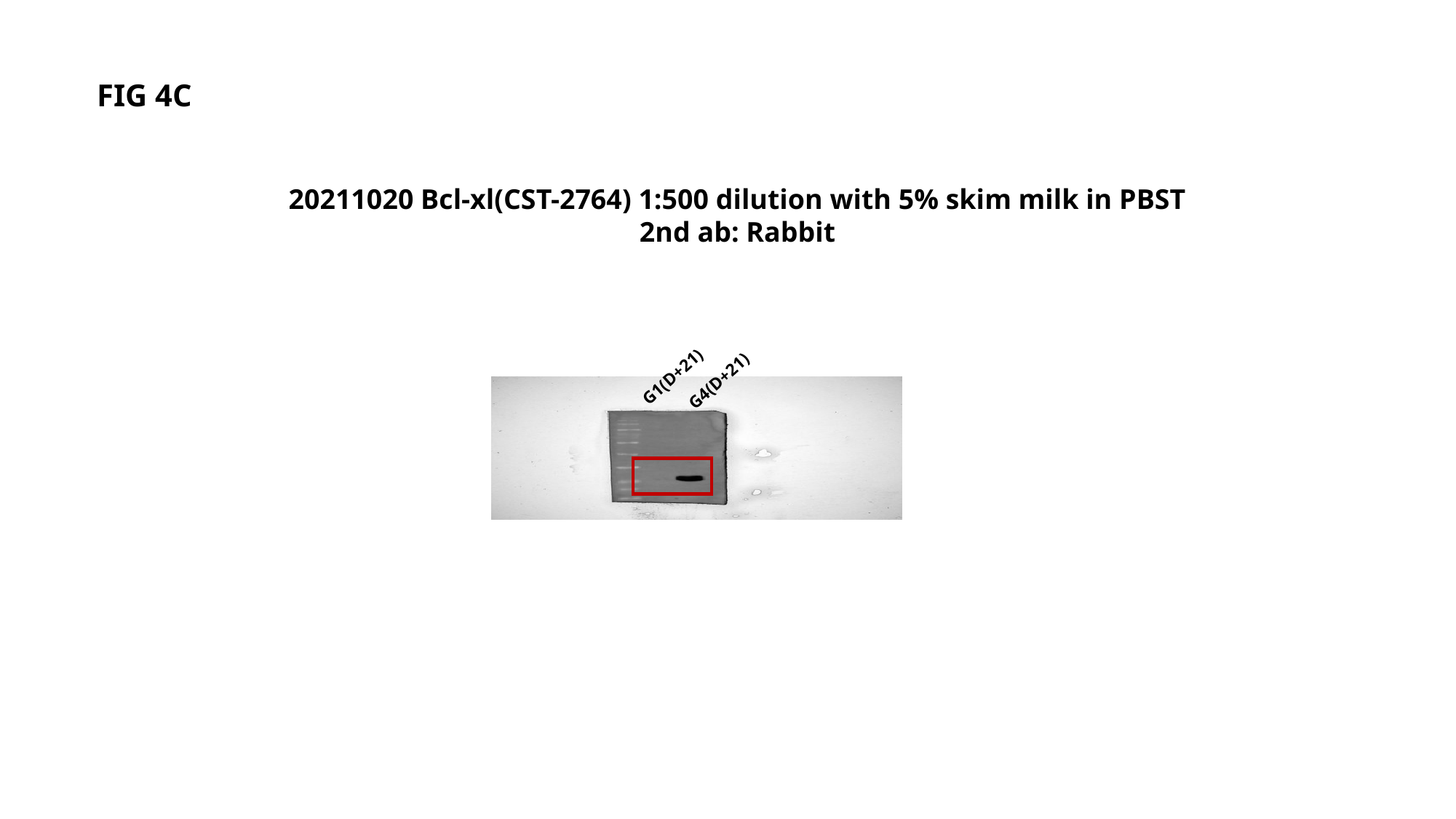

FIG 4C
20211020 Bcl-xl(CST-2764) 1:500 dilution with 5% skim milk in PBST 2nd ab: Rabbit
G1(D+21)
G4(D+21)

## Slide 5
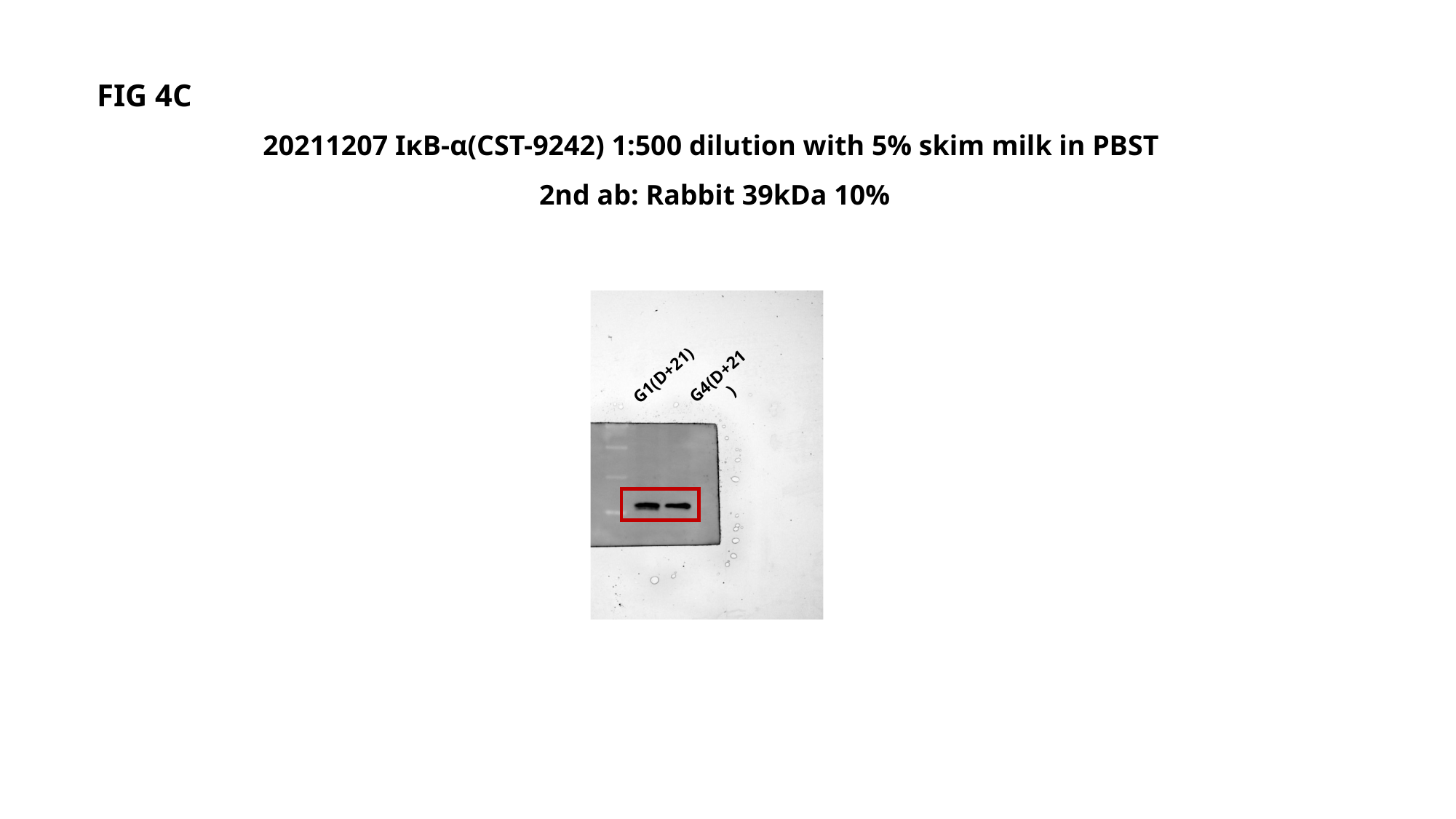

FIG 4C
20211207 IκB-α(CST-9242) 1:500 dilution with 5% skim milk in PBST 2nd ab: Rabbit 39kDa 10%
G1(D+21)
G4(D+21)

## Slide 6
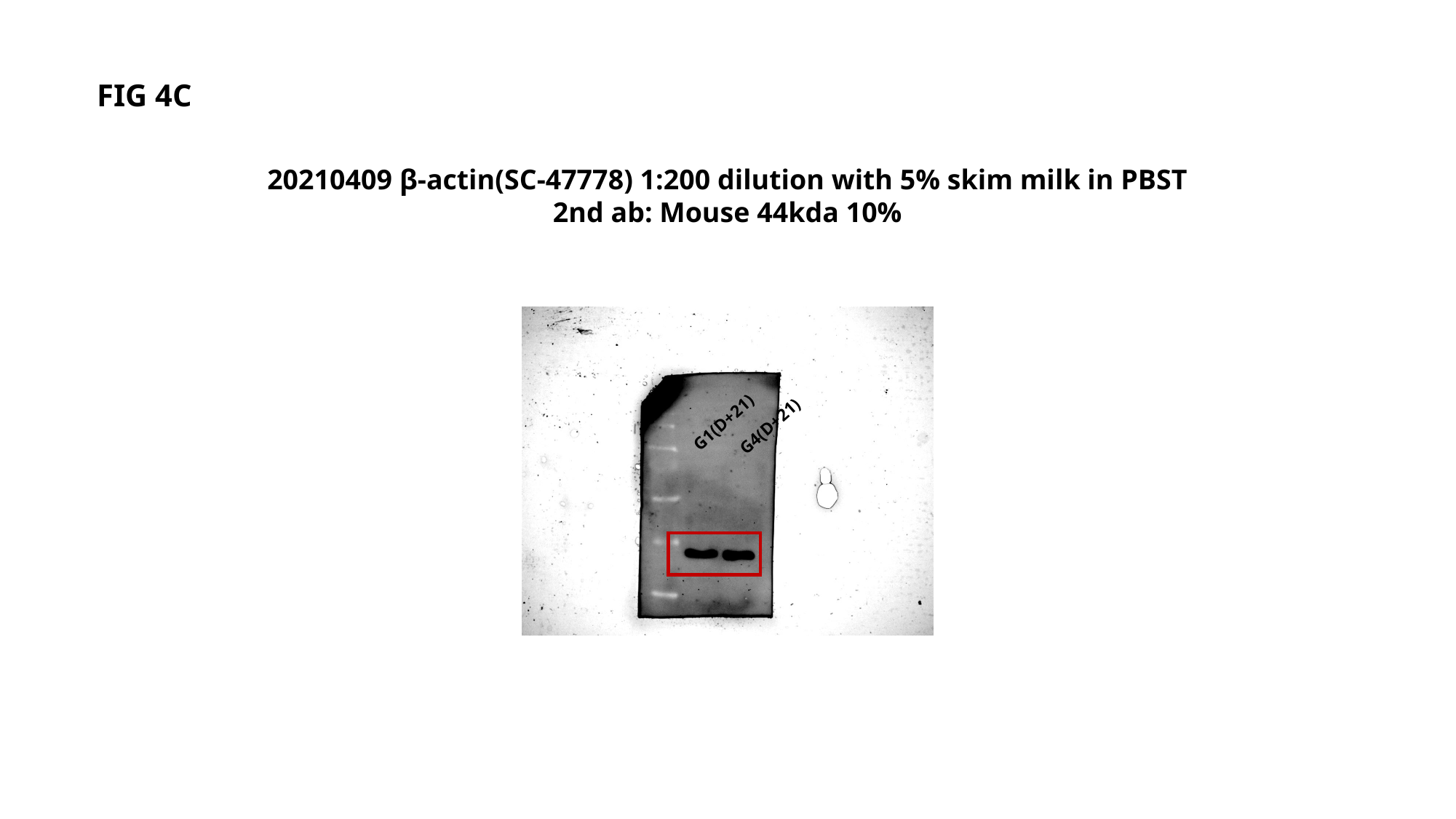

FIG 4C
20210409 β-actin(SC-47778) 1:200 dilution with 5% skim milk in PBST 2nd ab: Mouse 44kda 10%
G1(D+21)
G4(D+21)

## Slide 7
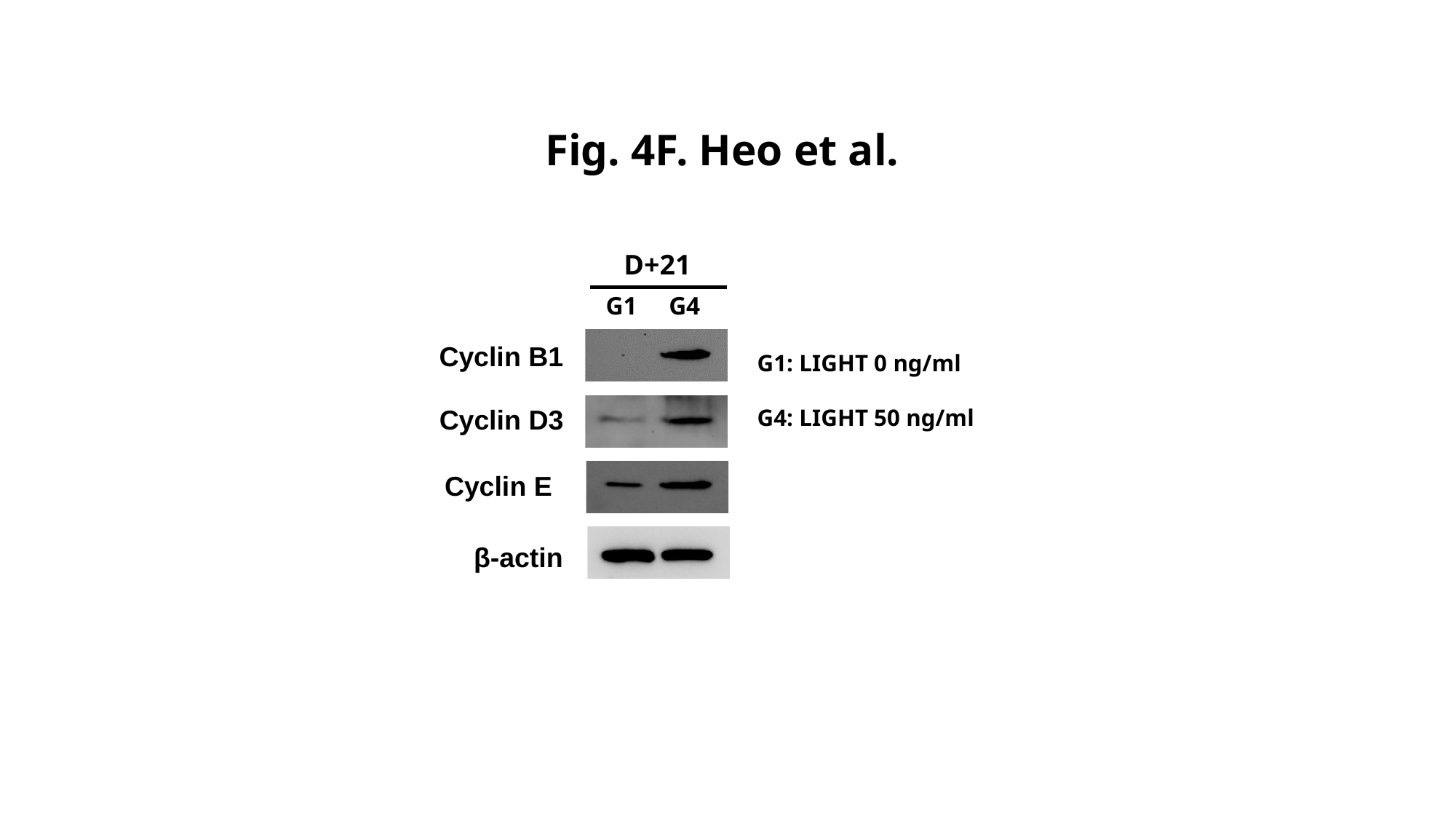

# Fig. 4F. Heo et al.
D+21
G1 G4
G1: LIGHT 0 ng/ml
G4: LIGHT 50 ng/ml
Cyclin B1
Cyclin D3
Cyclin E
β-actin

## Slide 8
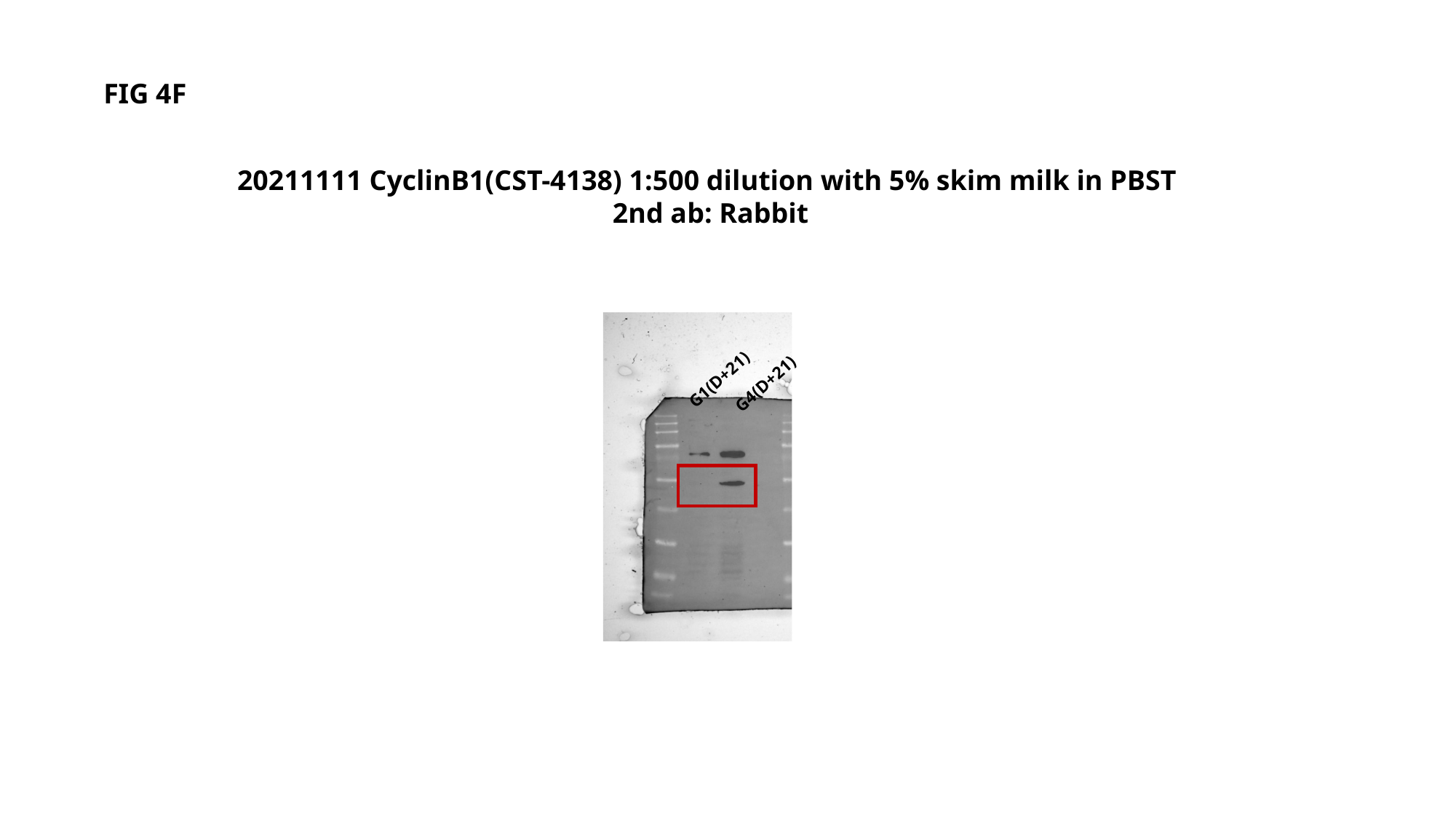

FIG 4F
20211111 CyclinB1(CST-4138) 1:500 dilution with 5% skim milk in PBST 2nd ab: Rabbit
G1(D+21)
G4(D+21)

## Slide 9
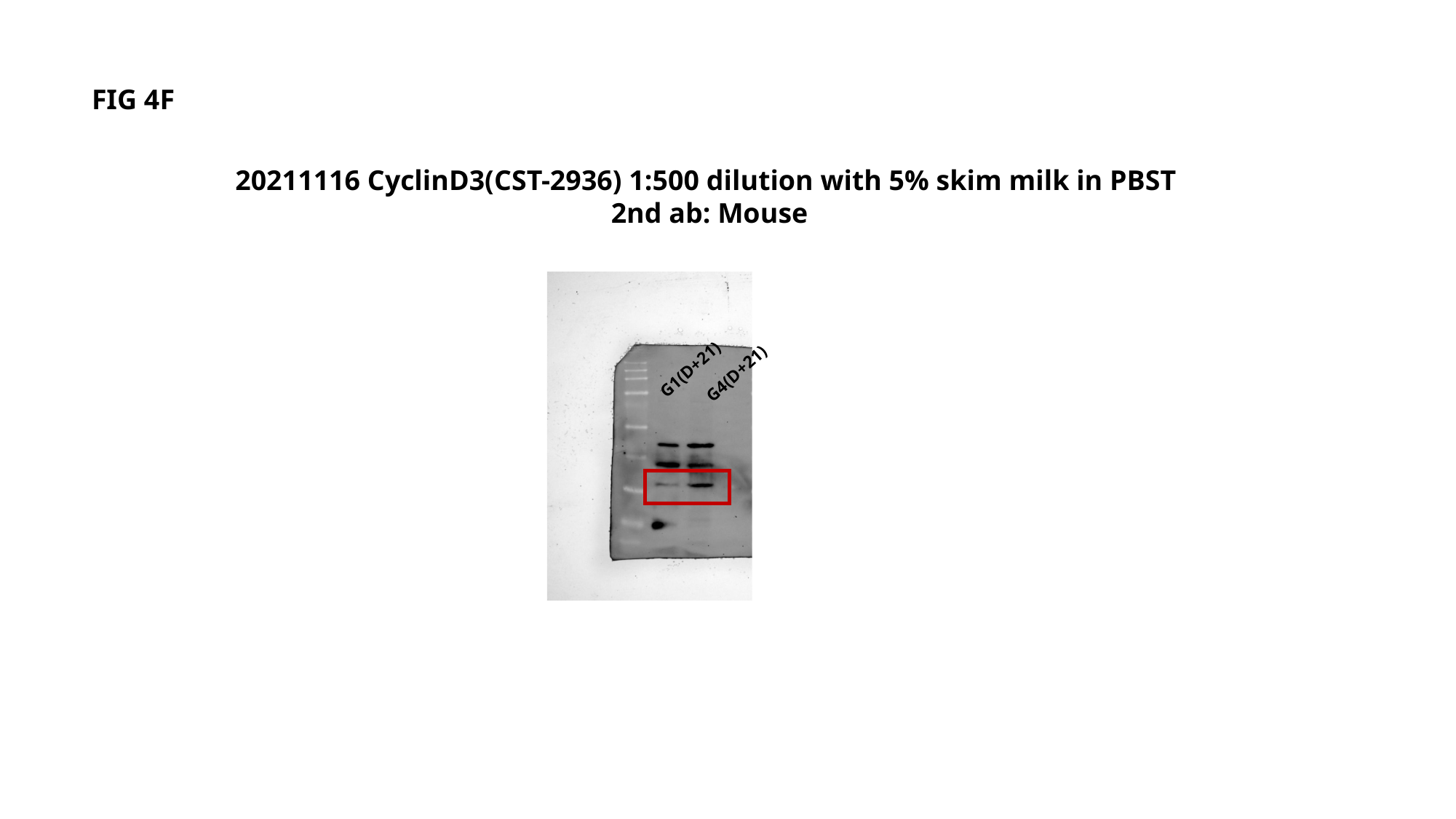

FIG 4F
20211116 CyclinD3(CST-2936) 1:500 dilution with 5% skim milk in PBST 2nd ab: Mouse
G1(D+21)
G4(D+21)

## Slide 10
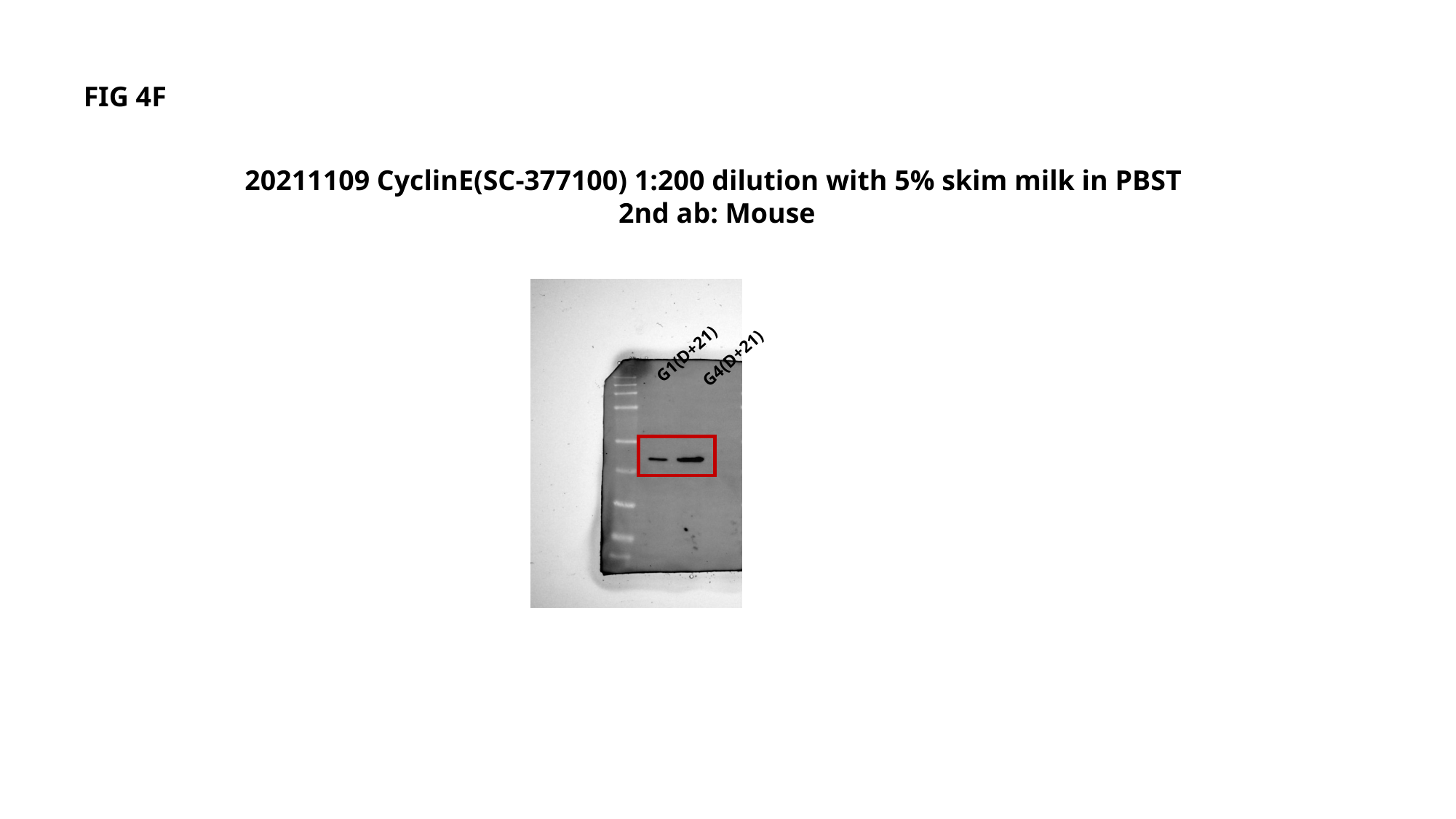

FIG 4F
20211109 CyclinE(SC-377100) 1:200 dilution with 5% skim milk in PBST 2nd ab: Mouse
G1(D+21)
G4(D+21)

## Slide 11
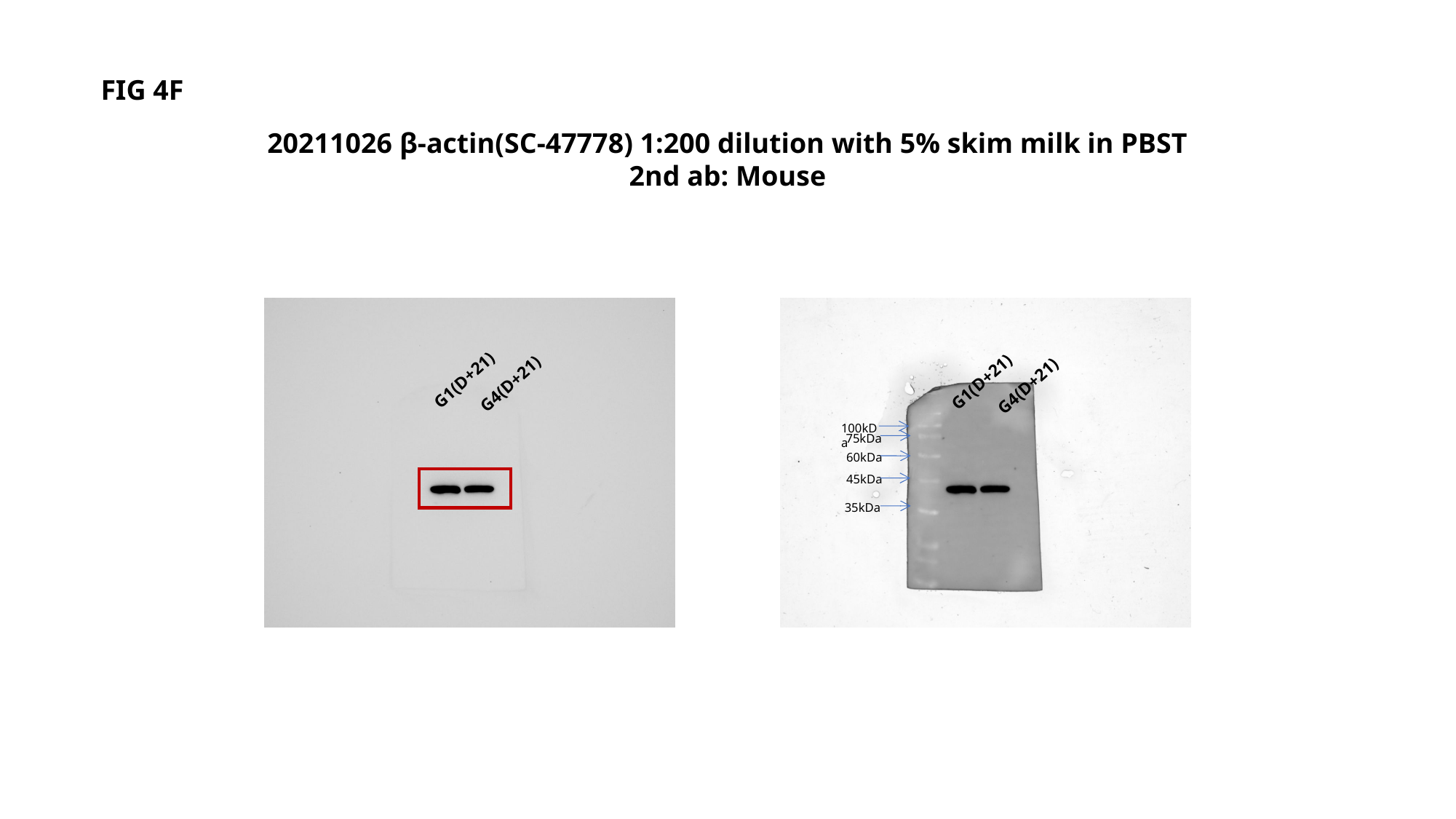

FIG 4F
20211026 β-actin(SC-47778) 1:200 dilution with 5% skim milk in PBST 2nd ab: Mouse
G1(D+21)
G4(D+21)
G1(D+21)
G4(D+21)
100kDa
60kDa
45kDa
35kDa
75kDa

## Slide 12
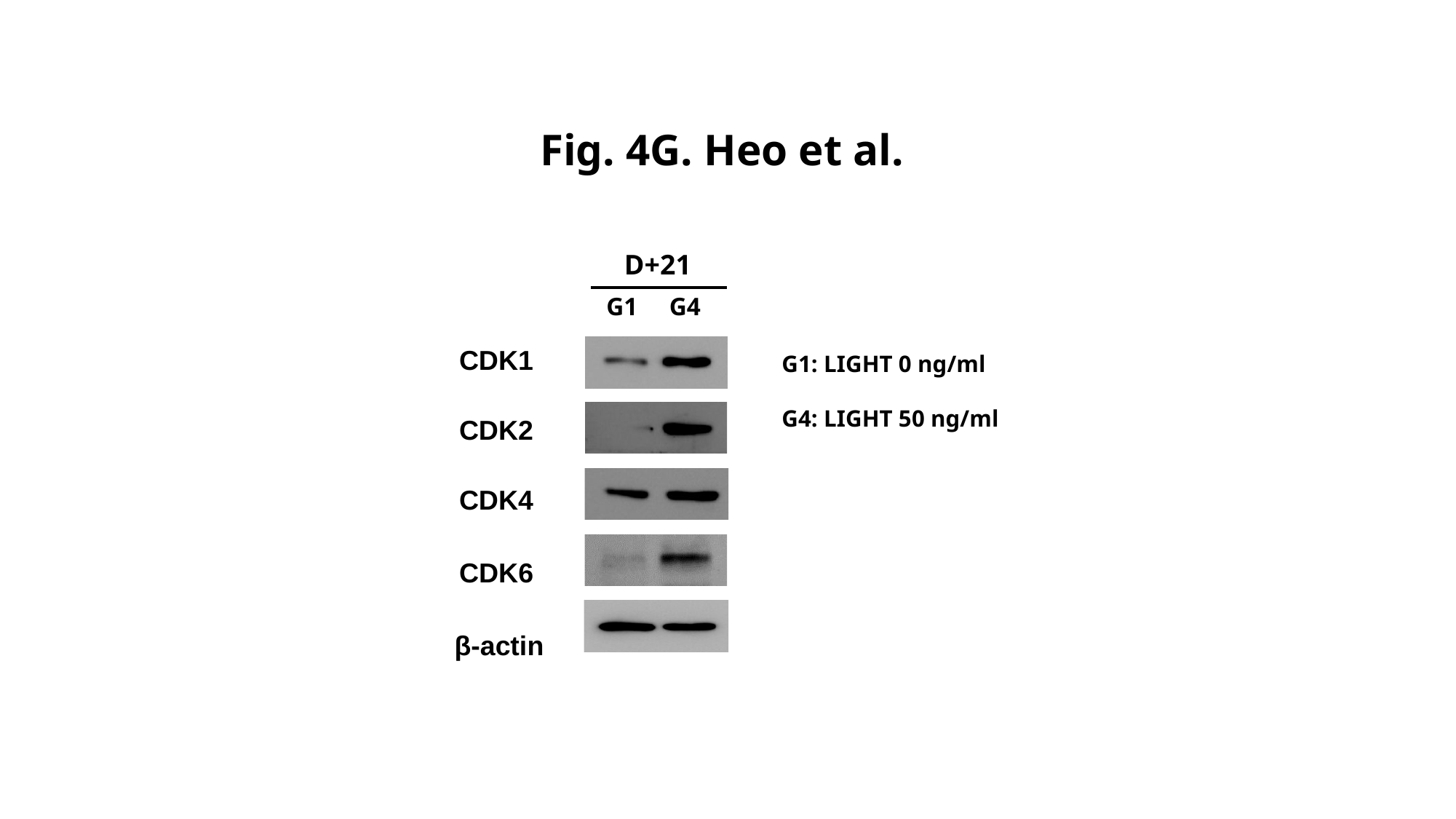

# Fig. 4G. Heo et al.
D+21
G1 G4
G1: LIGHT 0 ng/ml
G4: LIGHT 50 ng/ml
CDK1
CDK2
CDK4
CDK6
β-actin

## Slide 13
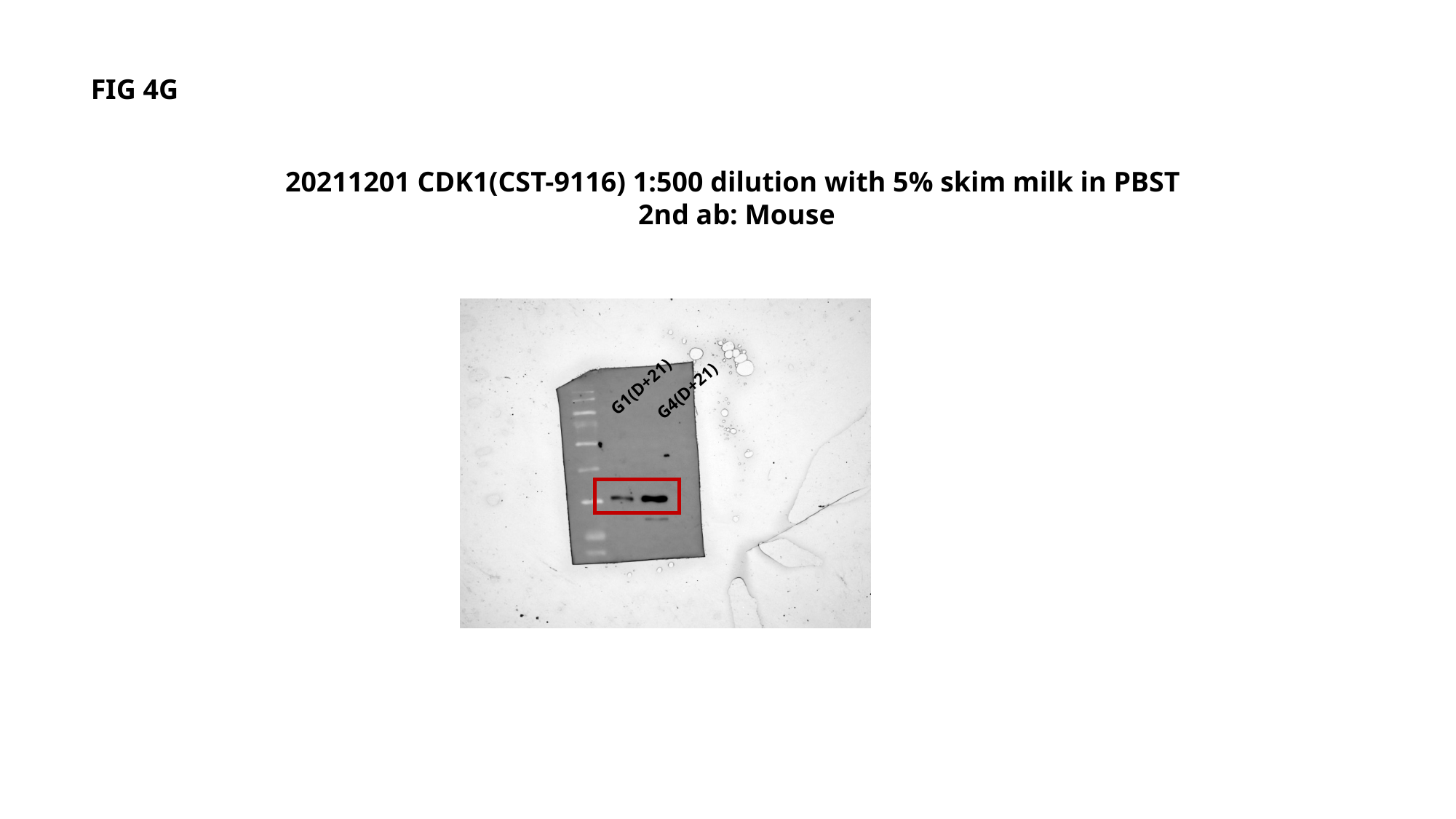

FIG 4G
20211201 CDK1(CST-9116) 1:500 dilution with 5% skim milk in PBST 2nd ab: Mouse
G1(D+21)
G4(D+21)

## Slide 14
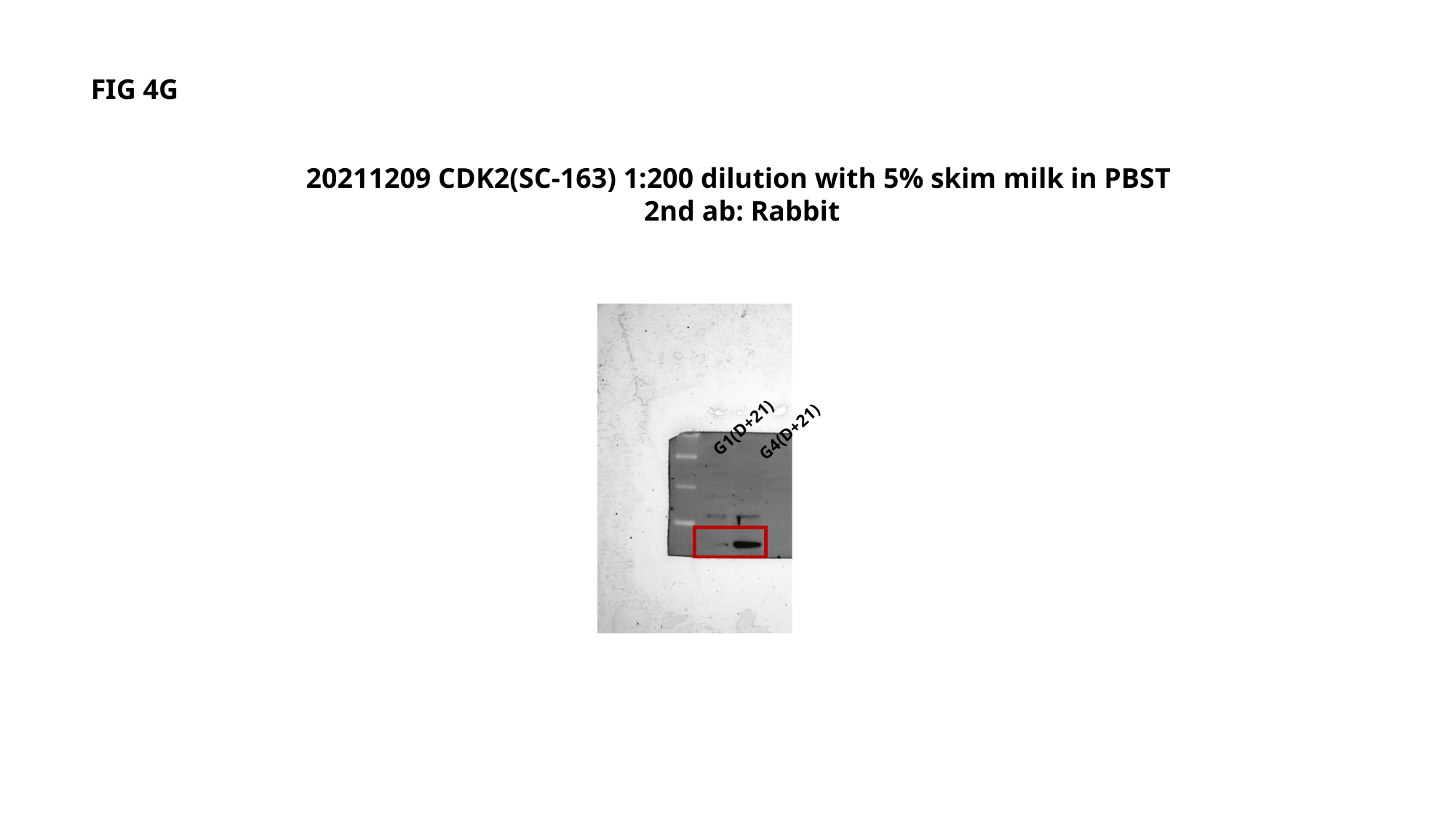

FIG 4G
20211209 CDK2(SC-163) 1:200 dilution with 5% skim milk in PBST 2nd ab: Rabbit
G1(D+21)
G4(D+21)

## Slide 15
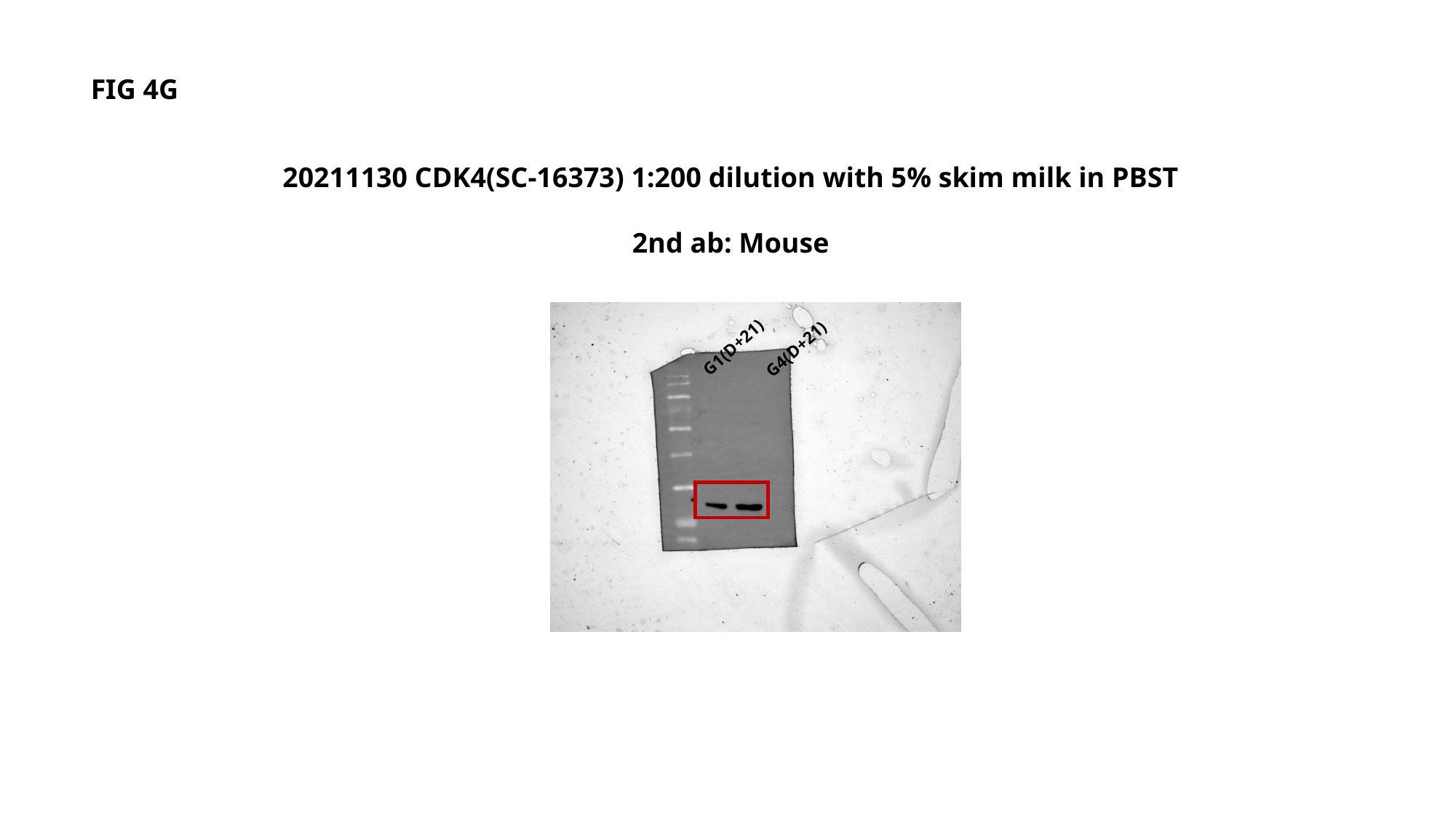

FIG 4G
20211130 CDK4(SC-16373) 1:200 dilution with 5% skim milk in PBST
2nd ab: Mouse
G1(D+21)
G4(D+21)

## Slide 16
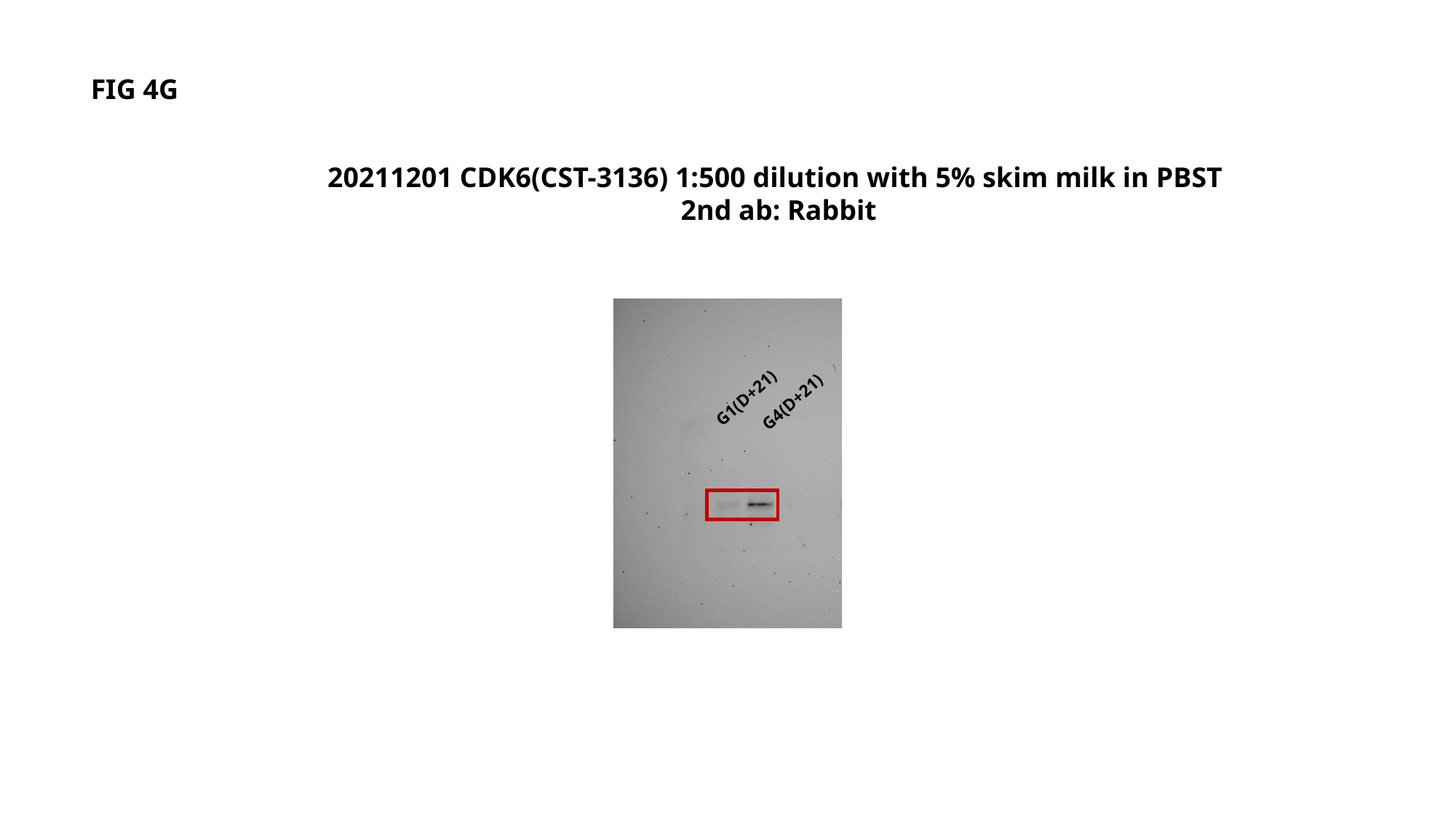

FIG 4G
20211201 CDK6(CST-3136) 1:500 dilution with 5% skim milk in PBST 2nd ab: Rabbit
G1(D+21)
G4(D+21)

## Slide 17
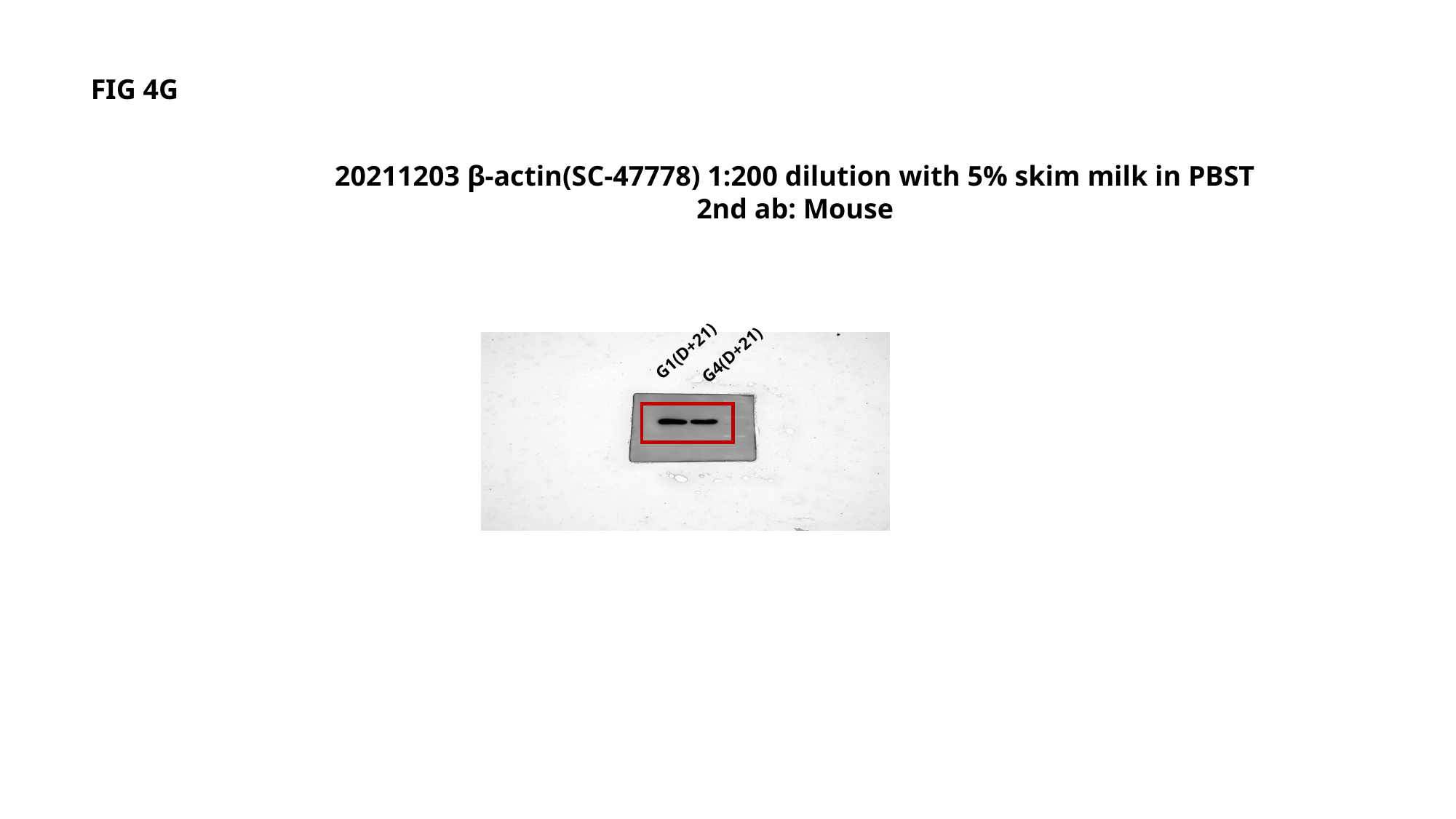

FIG 4G
20211203 β-actin(SC-47778) 1:200 dilution with 5% skim milk in PBST 2nd ab: Mouse
G1(D+21)
G4(D+21)

## Slide 18
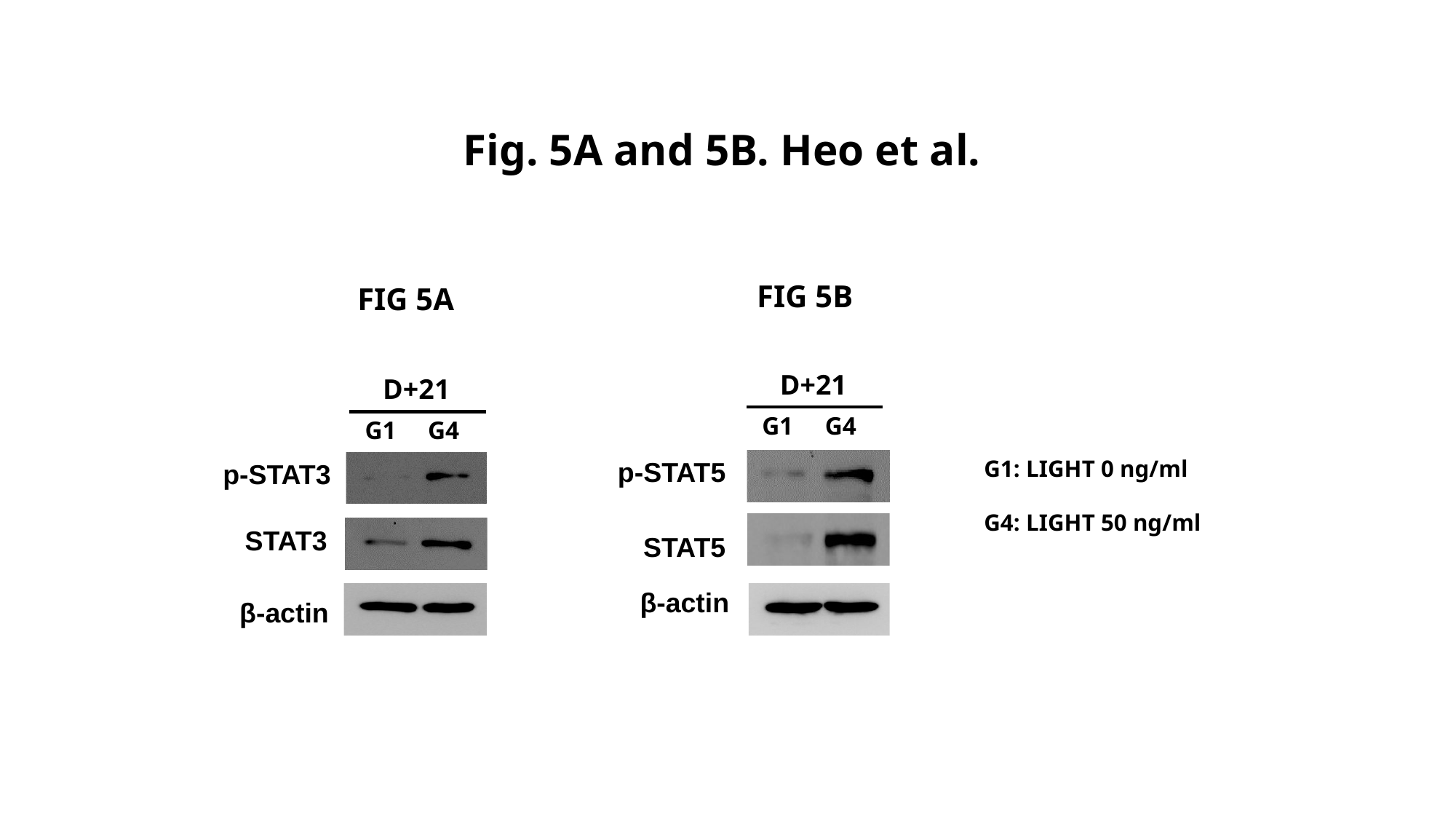

# Fig. 5A and 5B. Heo et al.
FIG 5B
FIG 5A
D+21
D+21
G1 G4
G1 G4
G1: LIGHT 0 ng/ml
G4: LIGHT 50 ng/ml
p-STAT5
p-STAT3
STAT3
STAT5
β-actin
β-actin

## Slide 19
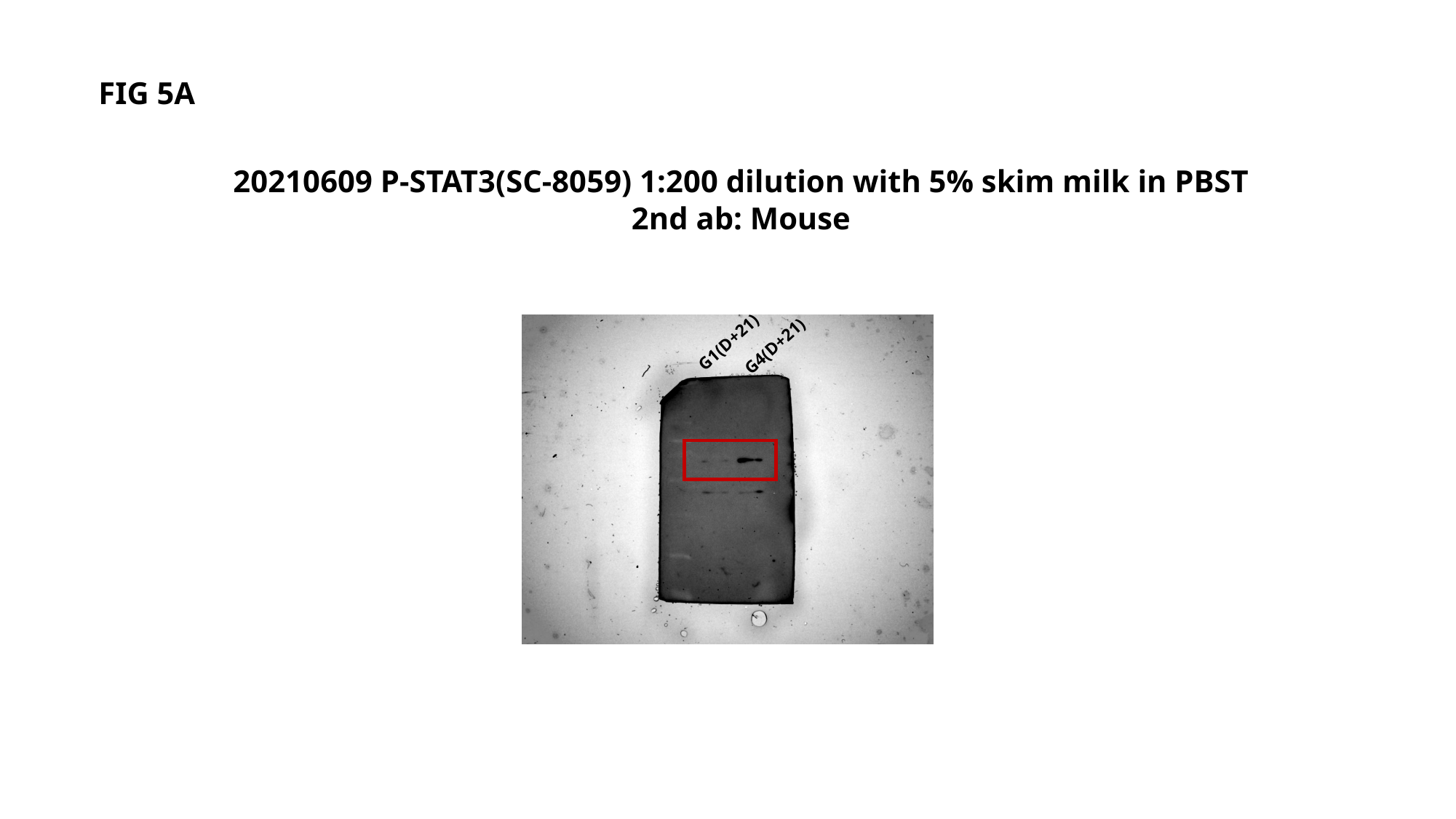

FIG 5A
20210609 P-STAT3(SC-8059) 1:200 dilution with 5% skim milk in PBST 2nd ab: Mouse
G1(D+21)
G4(D+21)

## Slide 20
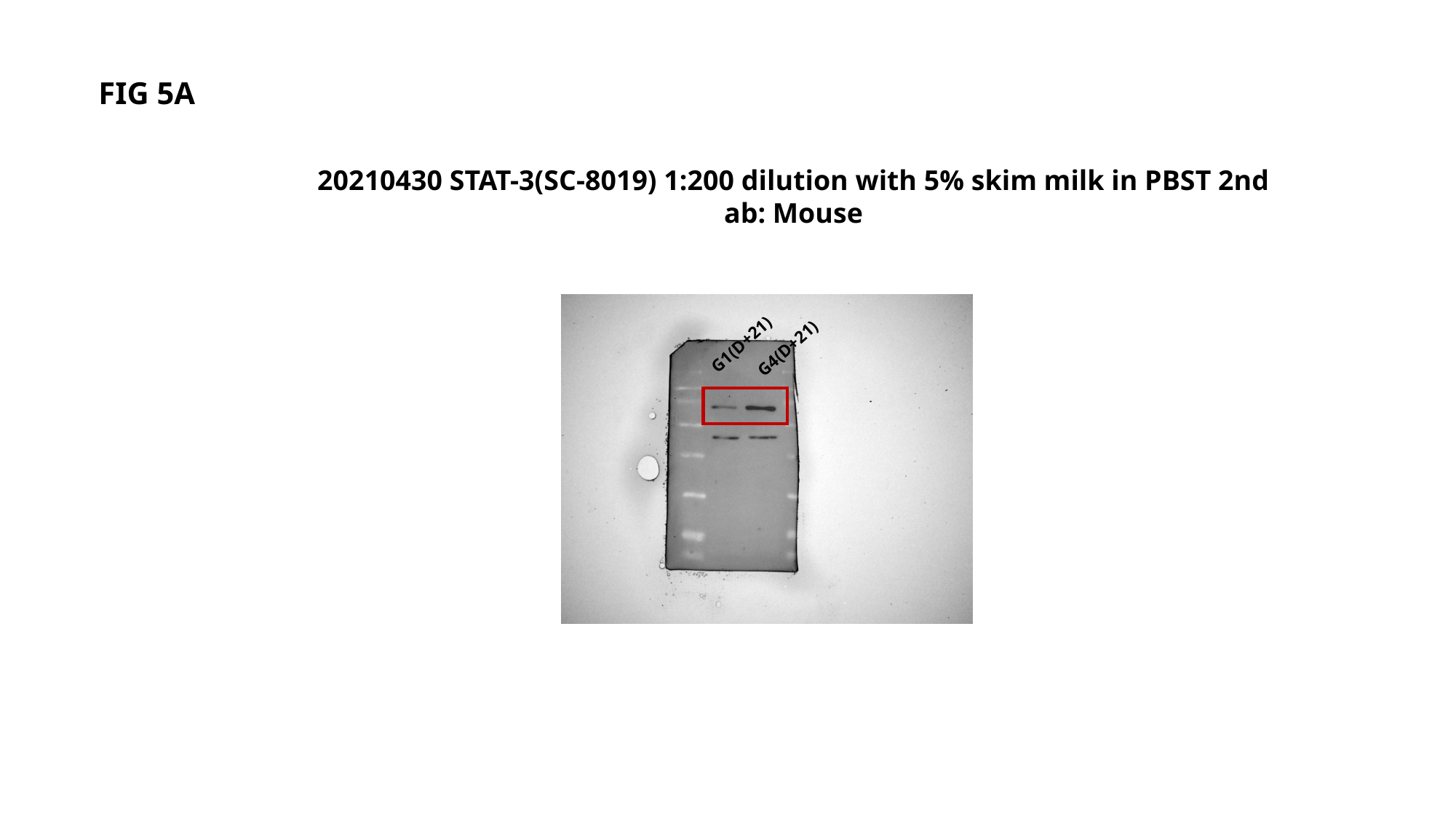

FIG 5A
20210430 STAT-3(SC-8019) 1:200 dilution with 5% skim milk in PBST 2nd ab: Mouse
G1(D+21)
G4(D+21)

## Slide 21
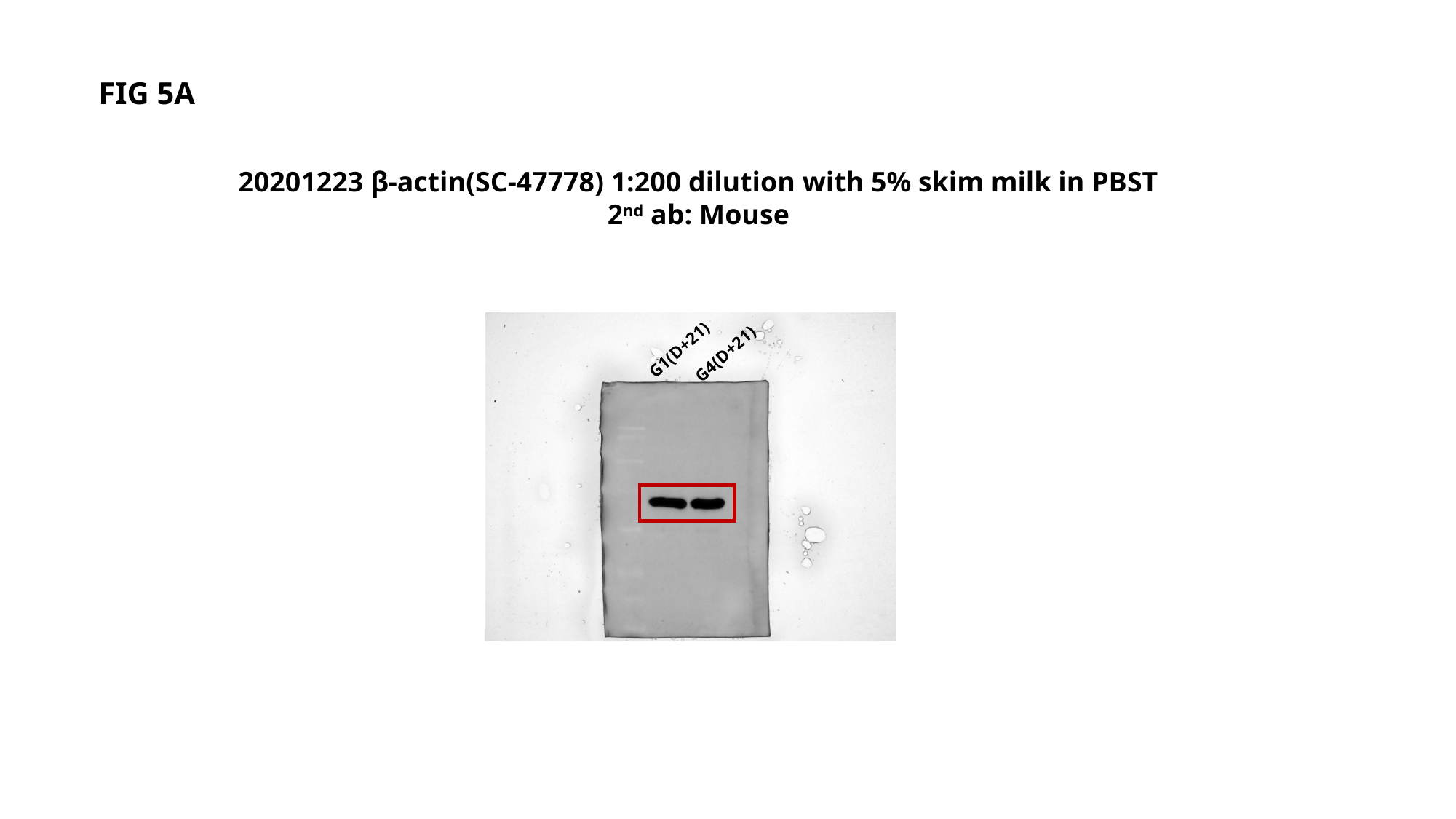

FIG 5A
20201223 β-actin(SC-47778) 1:200 dilution with 5% skim milk in PBST 2nd ab: Mouse
G1(D+21)
G4(D+21)

## Slide 22
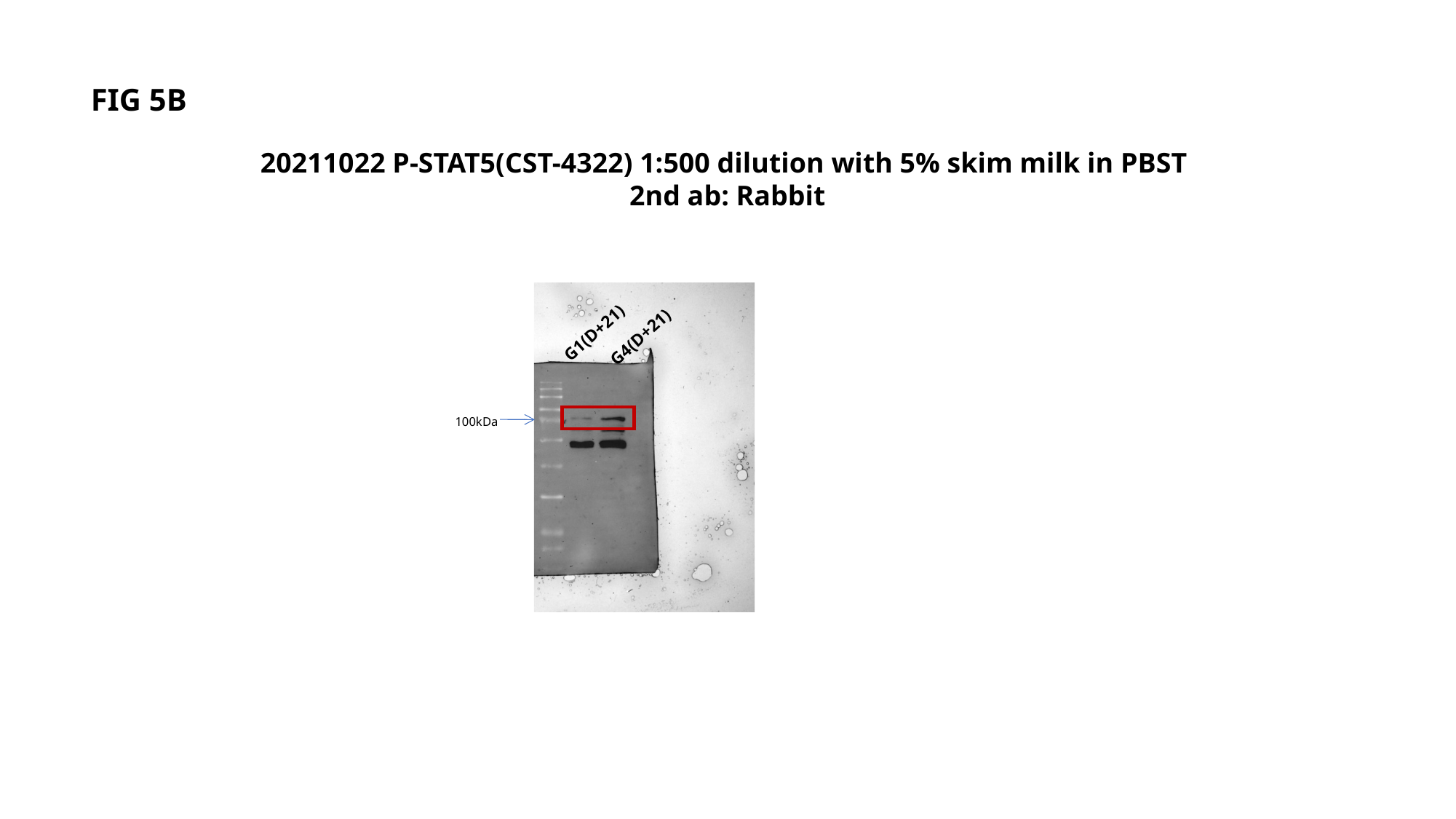

FIG 5B
20211022 P-STAT5(CST-4322) 1:500 dilution with 5% skim milk in PBST 2nd ab: Rabbit
G1(D+21)
G4(D+21)
100kDa

## Slide 23
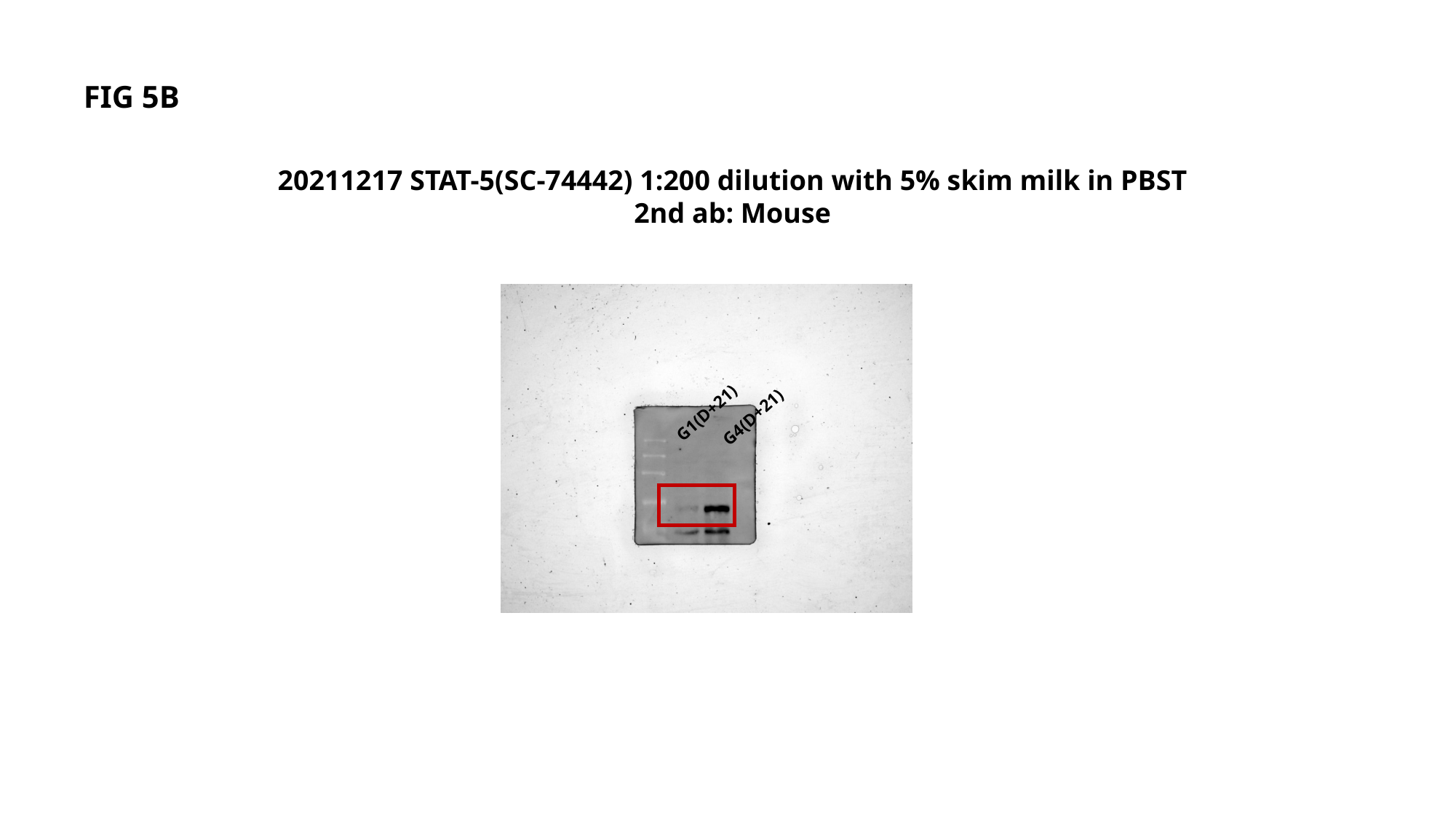

FIG 5B
20211217 STAT-5(SC-74442) 1:200 dilution with 5% skim milk in PBST 2nd ab: Mouse
G1(D+21)
G4(D+21)

## Slide 24
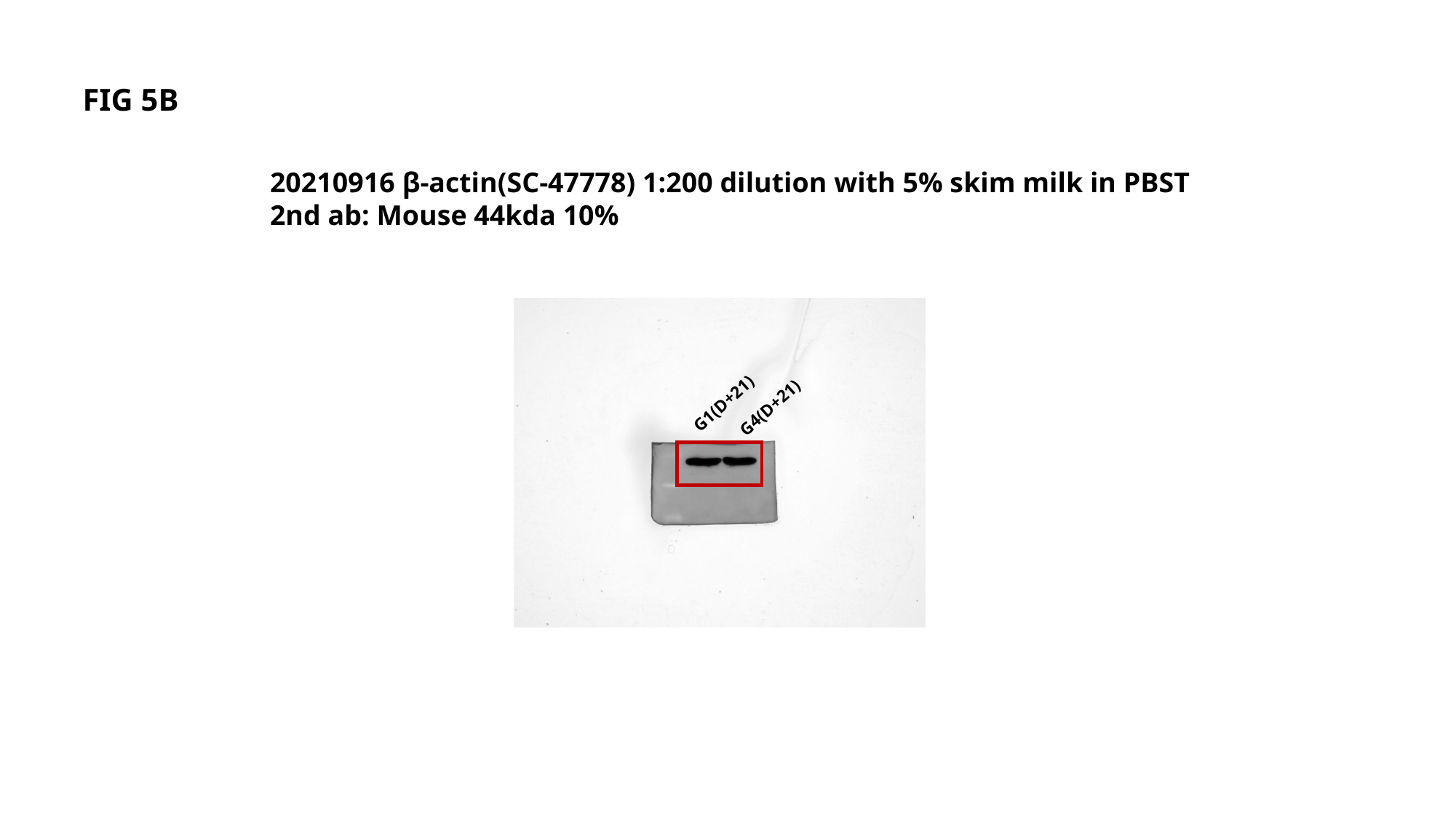

FIG 5B
20210916 β-actin(SC-47778) 1:200 dilution with 5% skim milk in PBST 2nd ab: Mouse 44kda 10%
G1(D+21)
G4(D+21)

## Slide 25
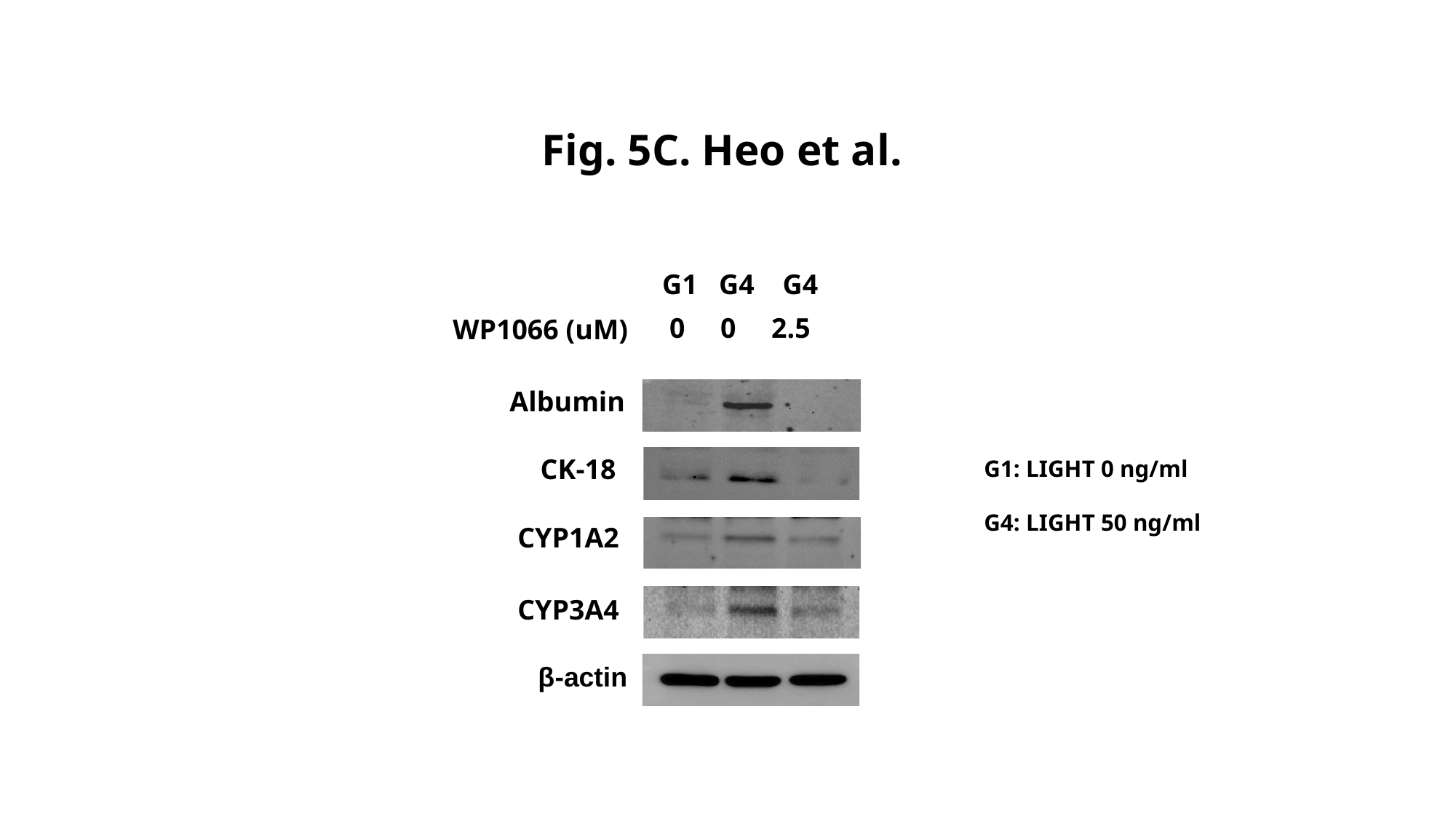

# Fig. 5C. Heo et al.
G1 G4 G4
 0 0 2.5
WP1066 (uM)
Albumin
CK-18
CYP1A2
CYP3A4
β-actin
G1: LIGHT 0 ng/ml
G4: LIGHT 50 ng/ml

## Slide 26
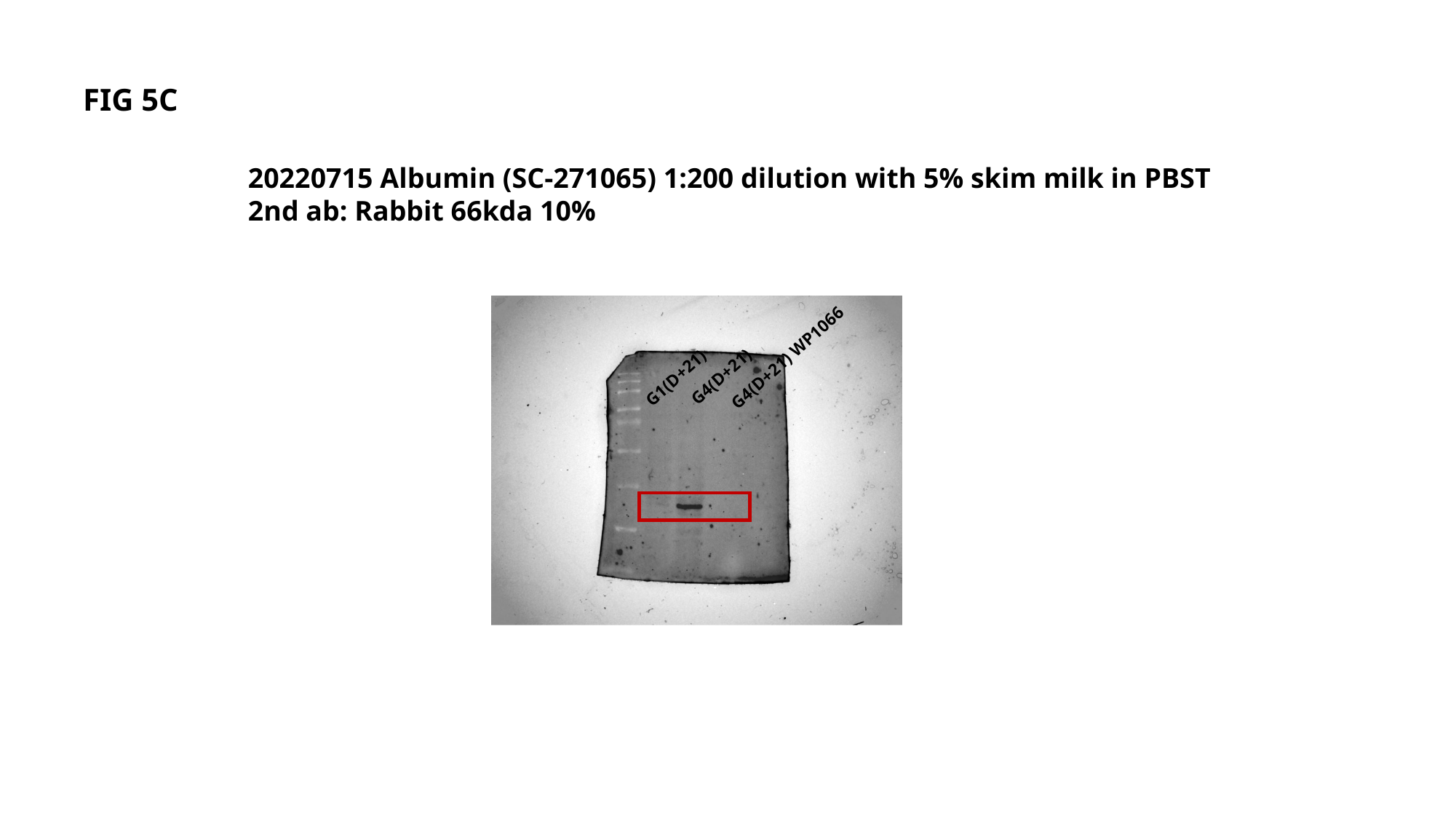

FIG 5C
20220715 Albumin (SC-271065) 1:200 dilution with 5% skim milk in PBST 2nd ab: Rabbit 66kda 10%
G4(D+21) WP1066
G4(D+21)
G1(D+21)

## Slide 27
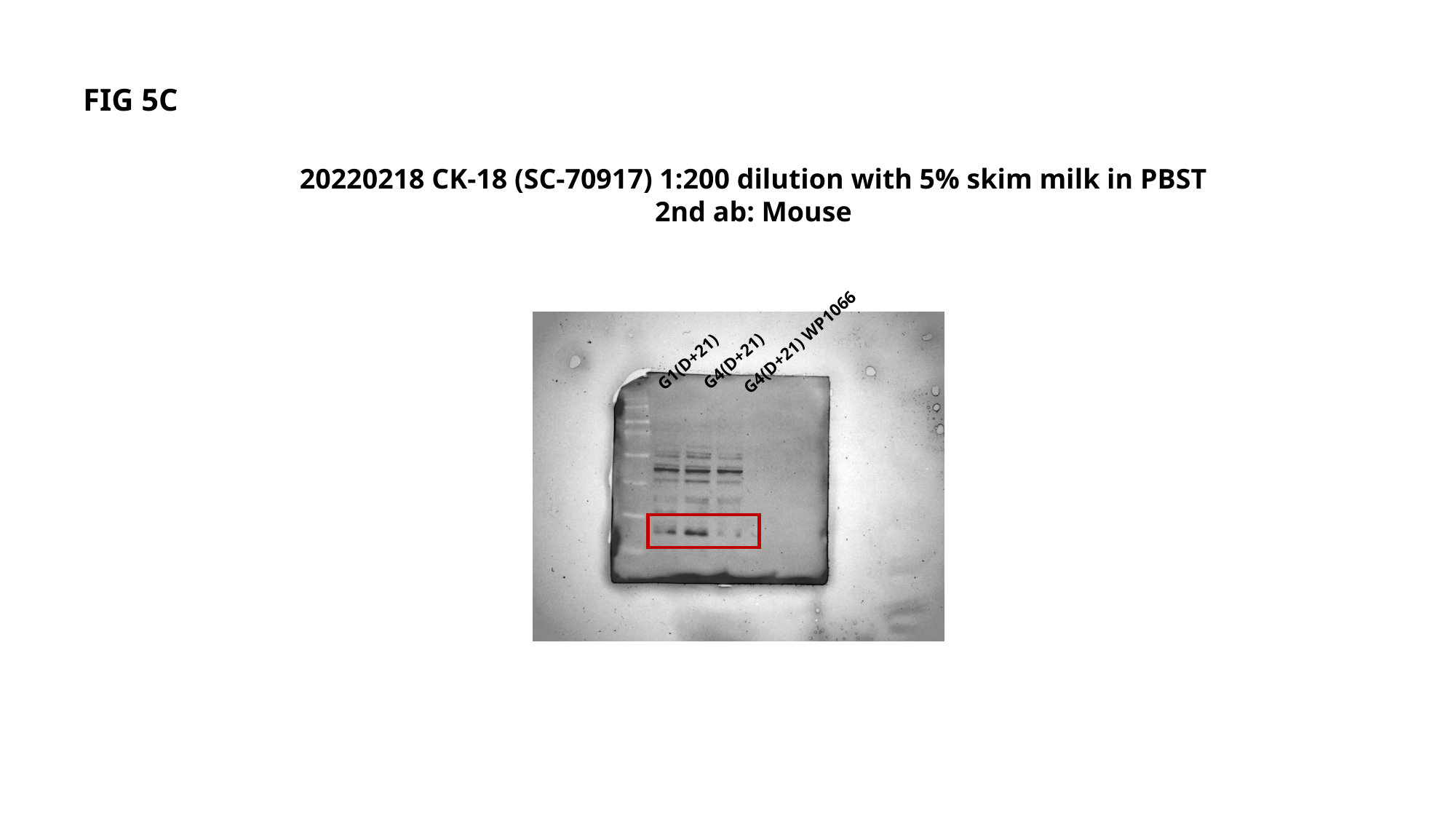

FIG 5C
20220218 CK-18 (SC-70917) 1:200 dilution with 5% skim milk in PBST
2nd ab: Mouse
G4(D+21) WP1066
G4(D+21)
G1(D+21)

## Slide 28
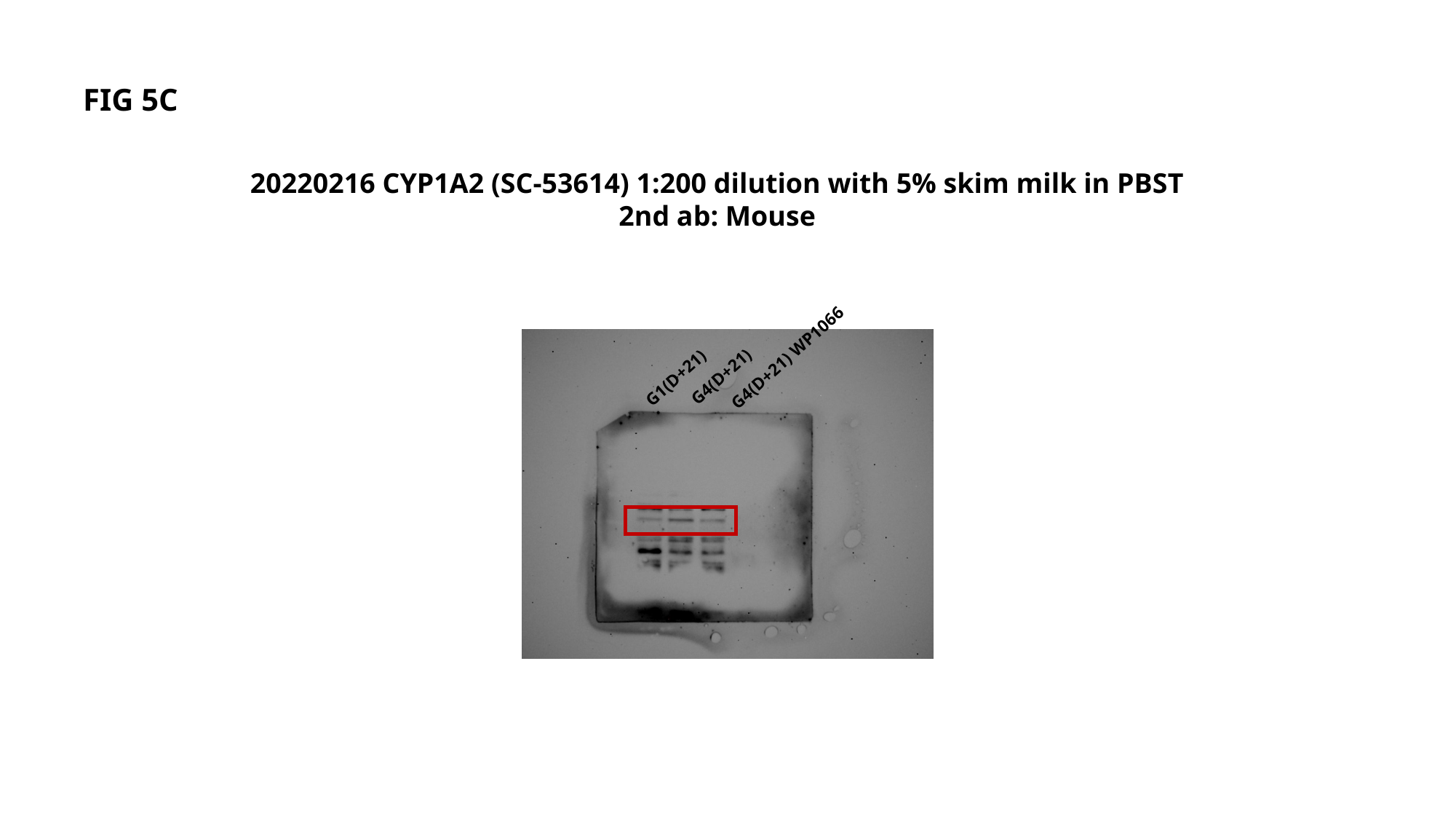

FIG 5C
20220216 CYP1A2 (SC-53614) 1:200 dilution with 5% skim milk in PBST 2nd ab: Mouse
G4(D+21) WP1066
G4(D+21)
G1(D+21)

## Slide 29
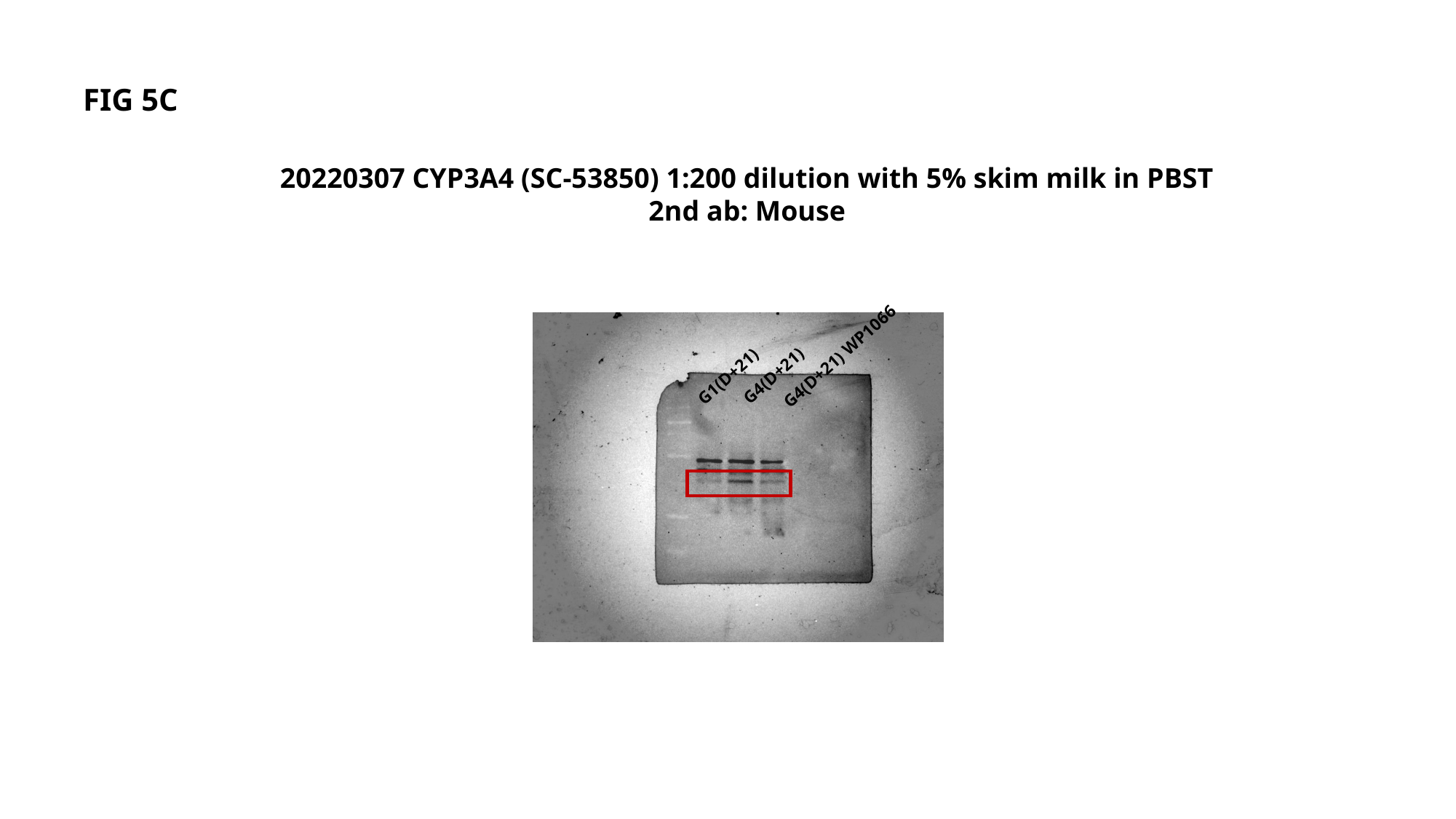

FIG 5C
20220307 CYP3A4 (SC-53850) 1:200 dilution with 5% skim milk in PBST 2nd ab: Mouse
G4(D+21) WP1066
G4(D+21)
G1(D+21)

## Slide 30
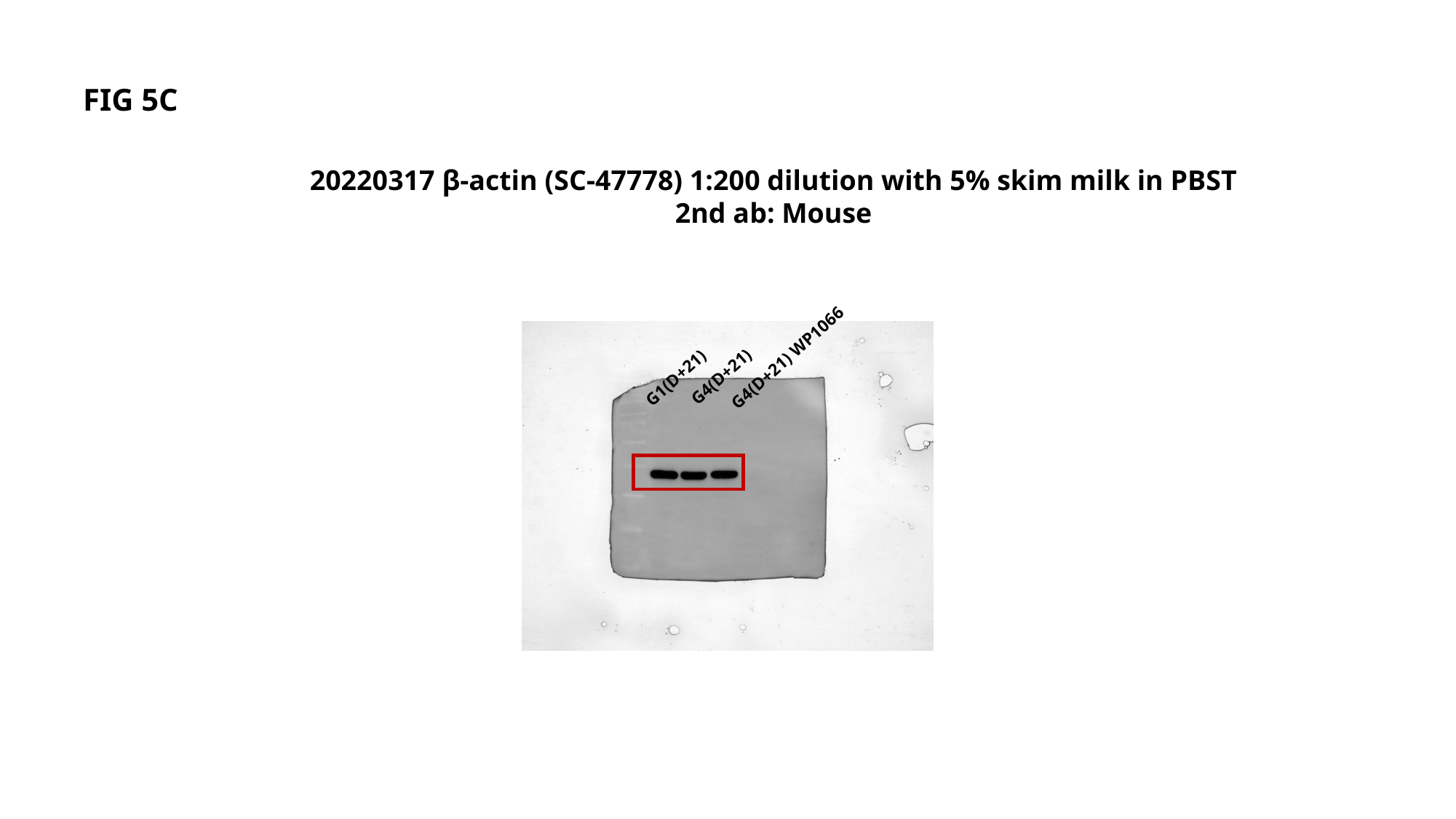

FIG 5C
20220317 β-actin (SC-47778) 1:200 dilution with 5% skim milk in PBST 2nd ab: Mouse
G4(D+21) WP1066
G4(D+21)
G1(D+21)

## Slide 31
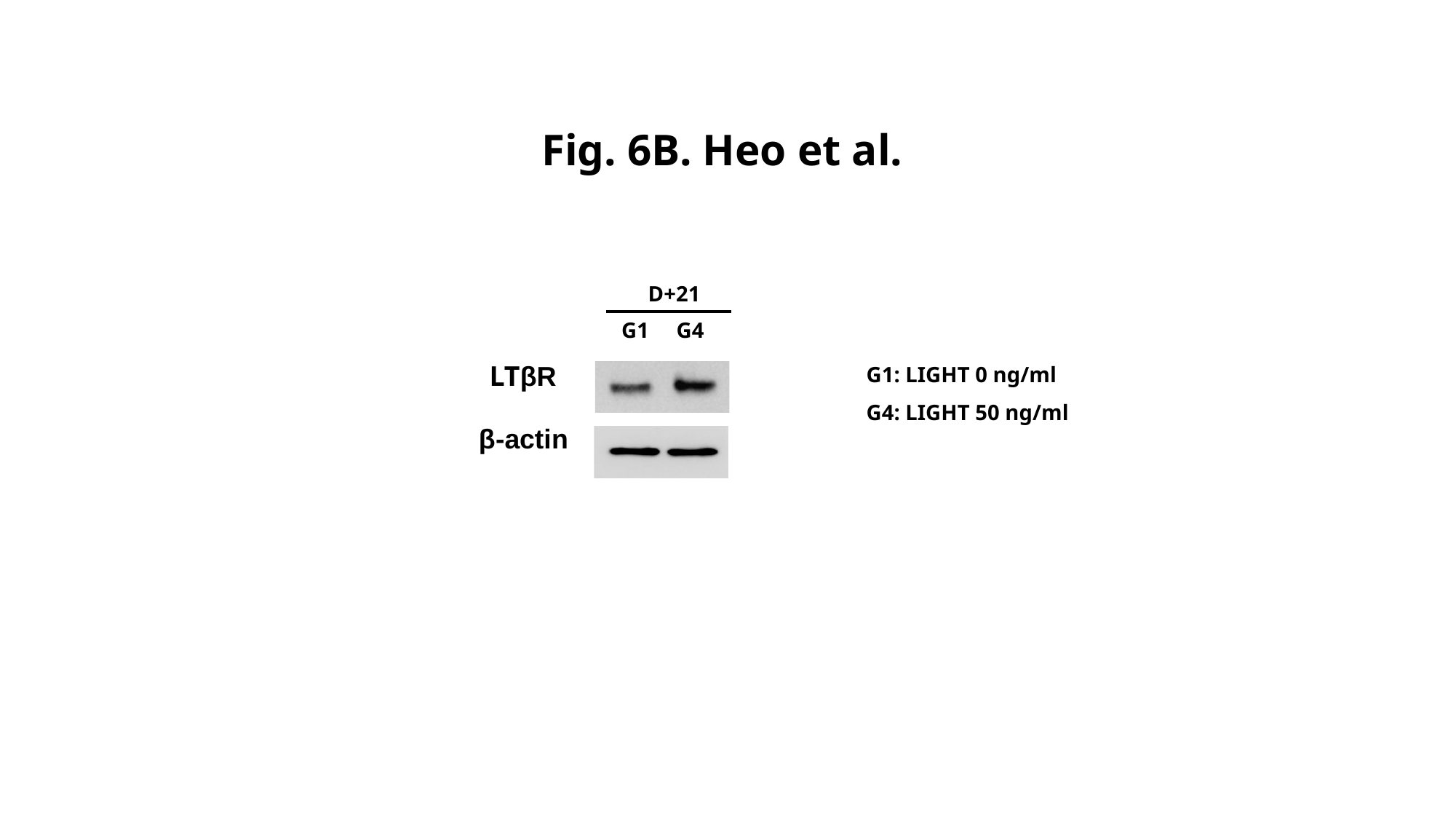

# Fig. 6B. Heo et al.
D+21
G1 G4
G1: LIGHT 0 ng/ml
G4: LIGHT 50 ng/ml
LTβR
β-actin

## Slide 32
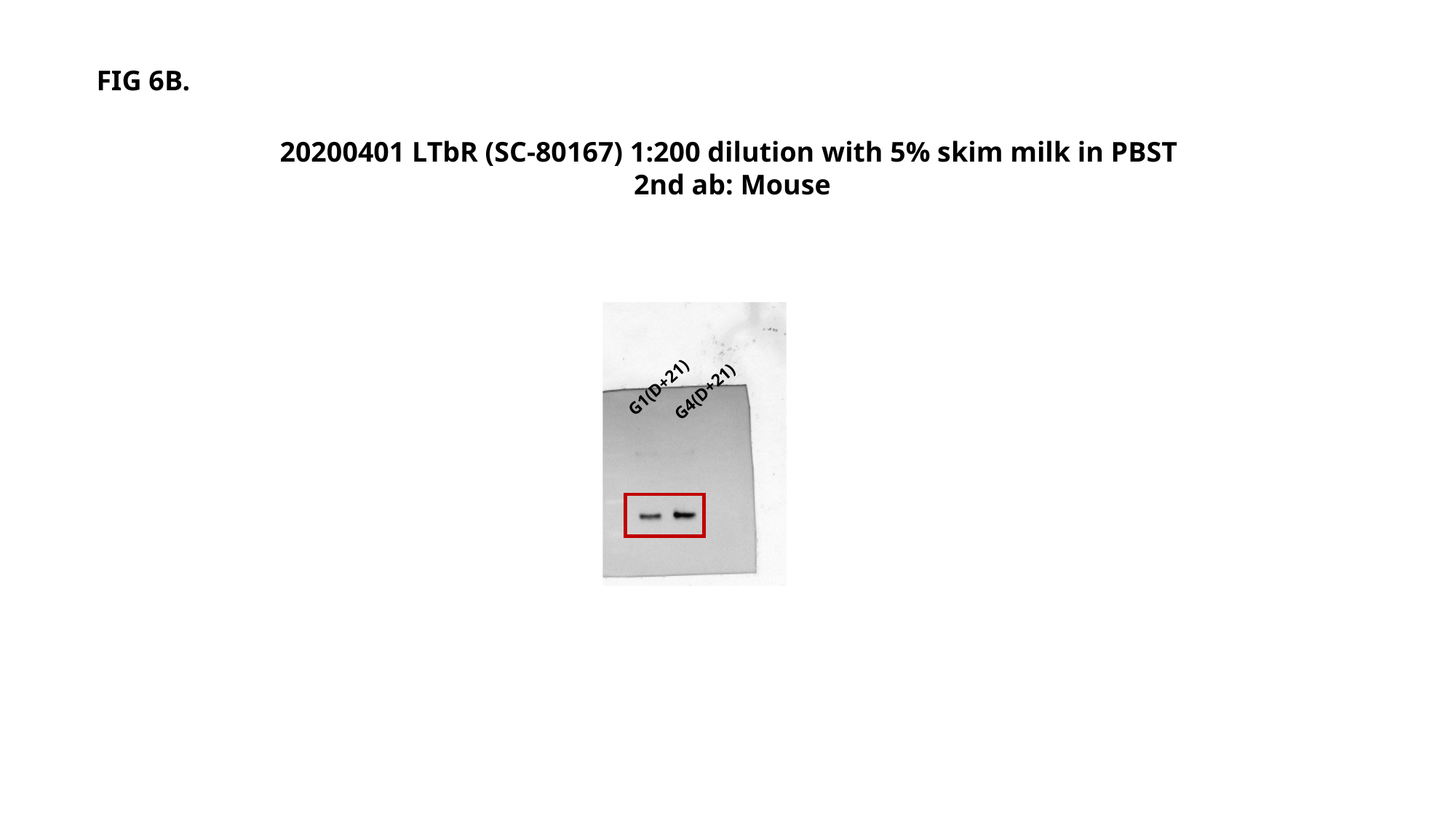

FIG 6B.
20200401 LTbR (SC-80167) 1:200 dilution with 5% skim milk in PBST
2nd ab: Mouse
G1(D+21)
G4(D+21)

## Slide 33
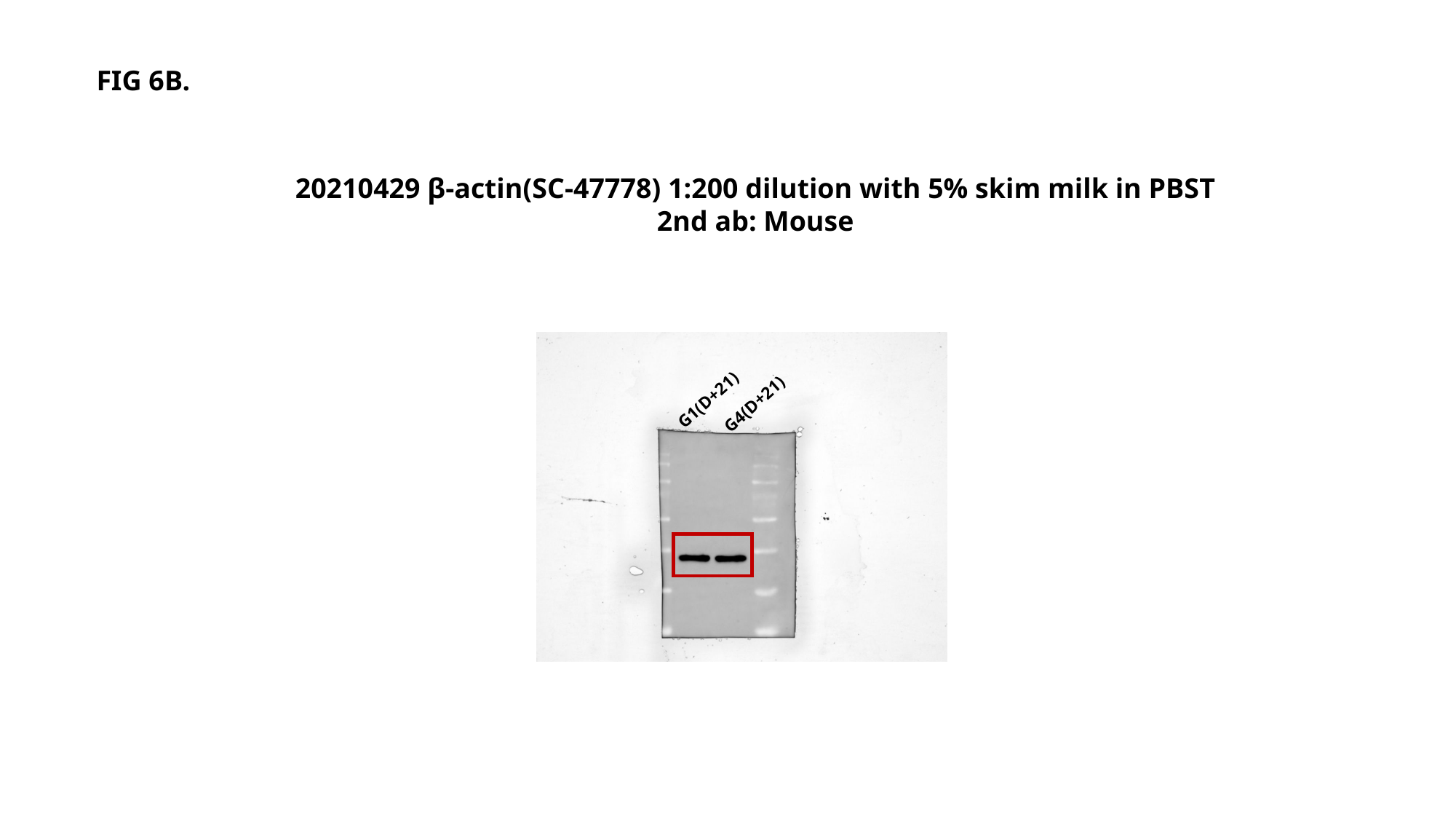

FIG 6B.
20210429 β-actin(SC-47778) 1:200 dilution with 5% skim milk in PBST 2nd ab: Mouse
G1(D+21)
G4(D+21)

## Slide 34
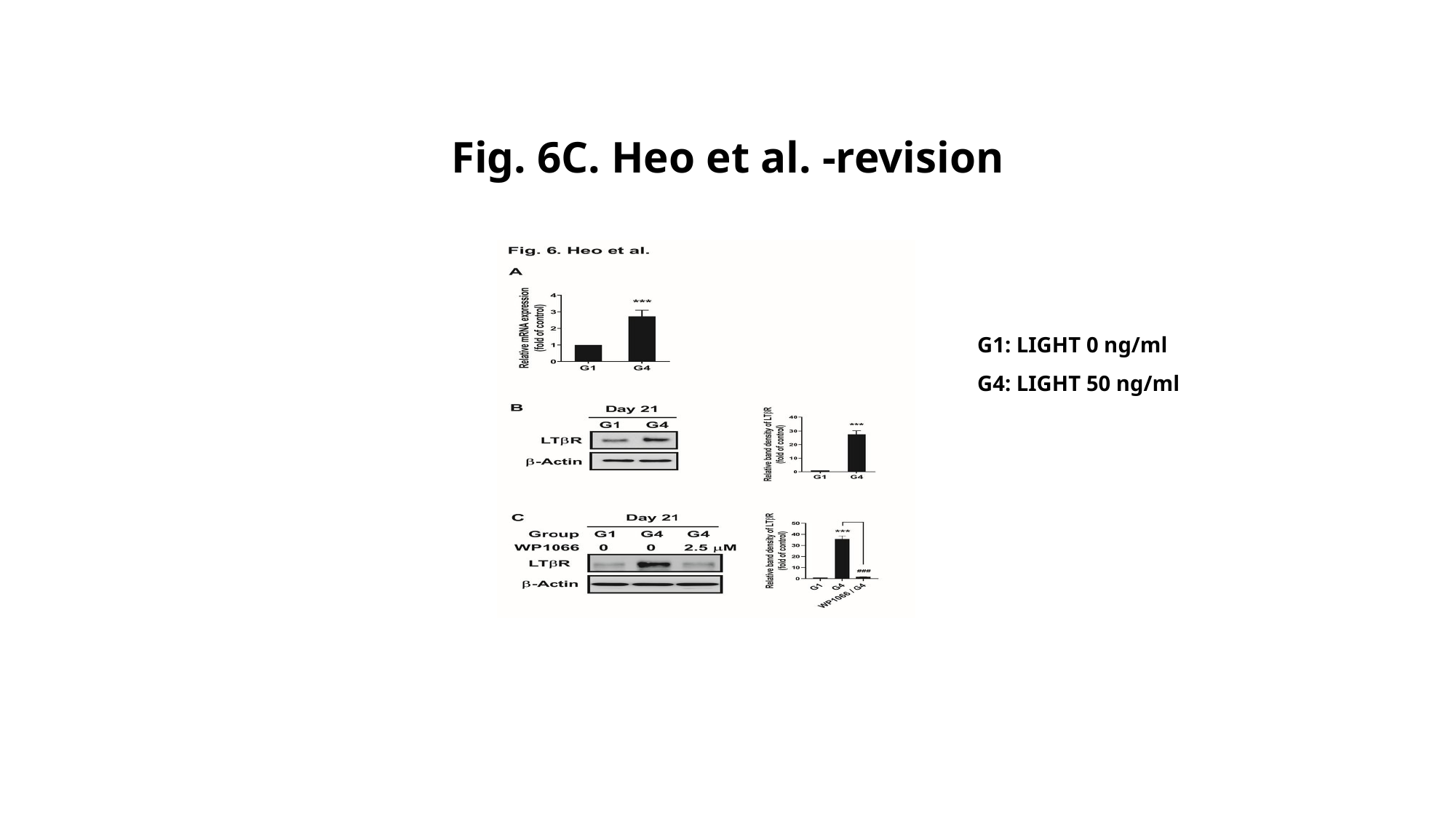

Fig. 6C. Heo et al. -revision
G1: LIGHT 0 ng/ml
G4: LIGHT 50 ng/ml

## Slide 35
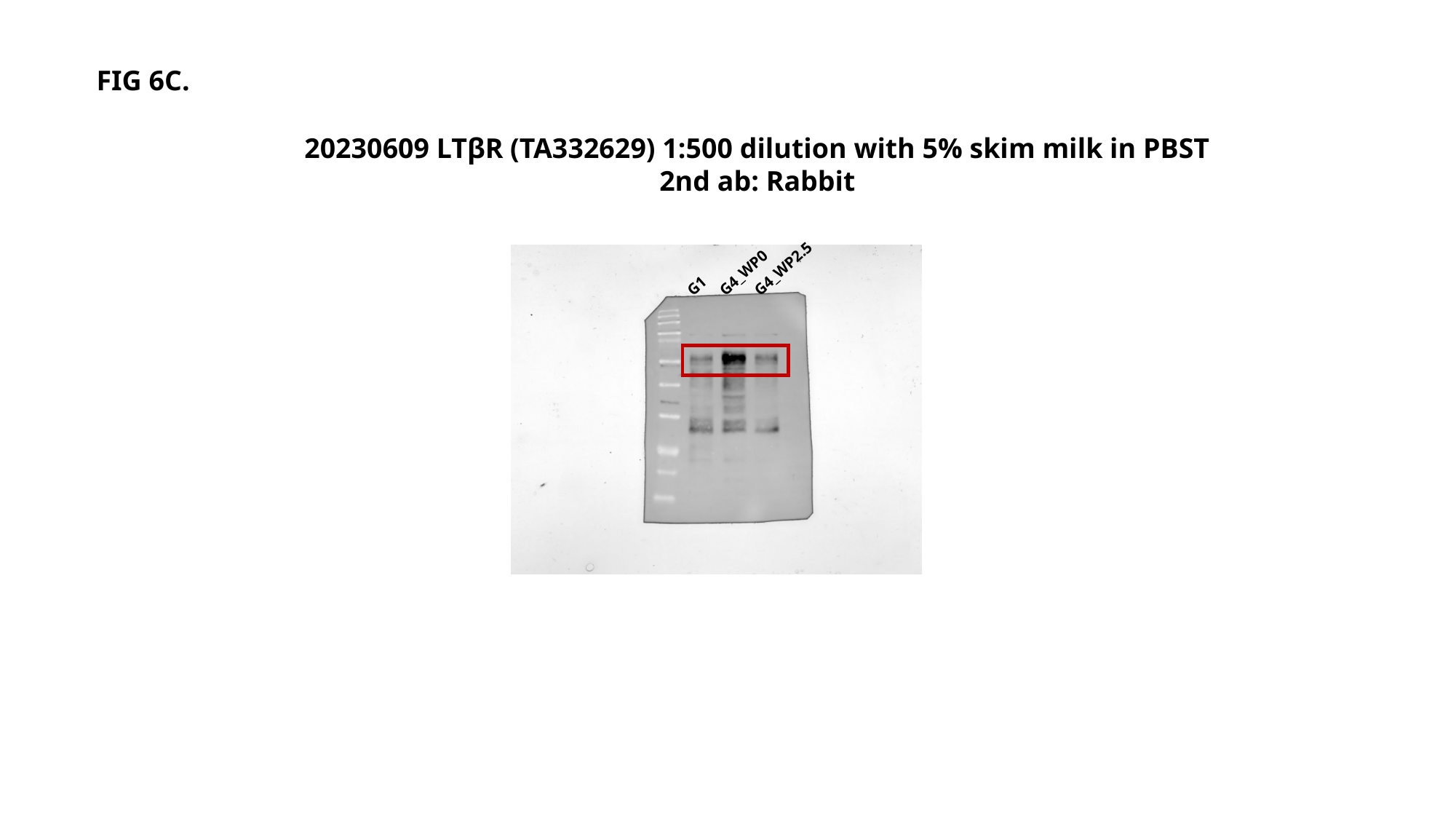

FIG 6C.
20230609 LTβR (TA332629) 1:500 dilution with 5% skim milk in PBST 2nd ab: Rabbit
G4_WP2.5
G4_WP0
G1

## Slide 36
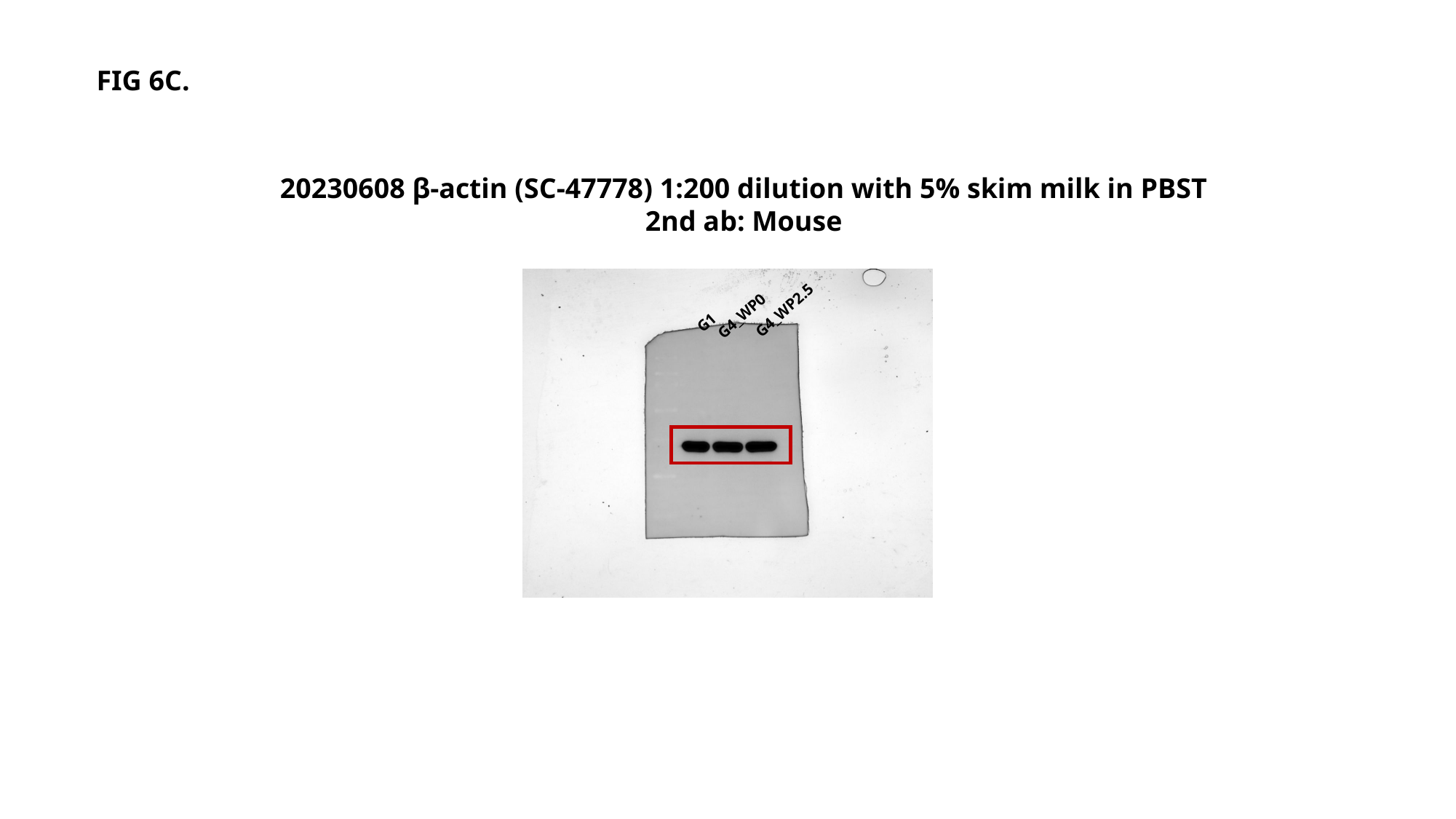

FIG 6C.
20230608 β-actin (SC-47778) 1:200 dilution with 5% skim milk in PBST
2nd ab: Mouse
G4_WP2.5
G4_WP0
G1
